# Field and classroom initiatives for portable sequence-based monitoring of dengue virus in Brazil

**DOI:** 10.1038/s41467-021-22607-0

**Published:** 2021-04-16

**Authors:** Talita Émile Ribeiro Adelino, Marta Giovanetti, Vagner Fonseca, Joilson Xavier, Álvaro Salgado de Abreu, Valdinete Alves do Nascimento, Luiz Henrique Ferraz Demarchi, Marluce Aparecida Assunção Oliveira, Vinícius Lemes da Silva, Arabela Leal e. Silva de Mello, Gabriel Muricy Cunha, Roselene Hans Santos, Elaine Cristina de Oliveira, Jorge Antônio Chamon Júnior, Felipe Campos de Melo Iani, Ana Maria Bispo de Filippis, André Luiz de Abreu, Ronaldo de Jesus, Carlos Frederico Campelo de Albuquerque, Jairo Mendez Rico, Rodrigo Fabiano do Carmo Said, Joscélio Aguiar Silva, Noely Fabiana Oliveira de Moura, Priscila Leite, Lívia Carla Vinhal Frutuoso, Simone Kashima Haddad, Alexander Martínez, Fernanda Khouri Barreto, Cynthia Carolina Vazquez, Rivaldo Venâncio da Cunha, Emerson Luiz Lima Araújo, Stephane Fraga de Oliveira Tosta, Allison de Araújo Fabri, Flávia Löwen Levy Chalhoub, Poliana da Silva Lemos, Fernanda de Bruycker-Nogueira, Gislene Garcia de Castro Lichs, Marina Castilhos Souza Umaki Zardin, Fátima María Cardozo Segovia, Crhistinne Cavalheiro Maymone Gonçalves, Zoraida Del Carmen Fernandez Grillo, Svetoslav Nanev Slavov, Luiz Augusto Pereira, Ana Flávia Mendonça, Felicidade Mota Pereira, Jurandy Júnior Ferraz de Magalhães, Agenor de Castro Moreira dos Santos Júnior, Maricélia Maia de Lima, Rita Maria Ribeiro Nogueira, Aristóteles Góes-Neto, Vasco Ariston de Carvalho Azevedo, Dario Brock Ramalho, Wanderson Kleber Oliveira, Eduardo Marques Macario, Arnaldo Correia de Medeiros, Victor Pimentel, Latin American Genomic Surveillance Arboviral Network, Erenilde Marques de Cerqueira, Tiago Graf, Walter Ramalho, Wildo Navegantes, Renato Barbosa Reis, Clara Guerra Duarte, Maira Alves Pereira, Paulo Eduardo de Souza da Silva, Raoni Almeida de Souza, Alex Pauvolid-Corrêa, Anne Aline Pereira de Paiva, Hegger Machado Fritsch, Maria Angélica Mares-Guia, Maria Celeste Torres, Maurício Teixeira Lima, Patrícia Sequeira, William de Almeida Marques, Jorlan Fernandes de Jesus, Felipe Gomes Naveca, Alessandra Lima Silva, Anne Cybelle Pinto, Arun Kumar Jaiswal, Élisson Nogueira Lopes, Francielly Morais Rodrigues da Costa, Gabriel Quintanilha-Peixoto, Gilson Carlos Soares, Paula Luize Camargos Fonseca, Renan Pedra de Souza, Rodrigo Bentes Kato, Rodrigo Profeta Silveira Santos, Sandeep Tiwari, Wylerson Guimarães Nogueira, Beatriz Senra Álvares da Silva Santos, Bruna Lopes Bueno, Isadora Cristina de Siqueira, Lourdes Farre Vallve, Melina Mosquera Navarro Borba, Alix Sandra Mazzetto, Francisco de Assis Araújo Aguiar, Irenio da Silva Gomes, Jayra Juliana Paiva Alves Abrantes, Luiz Takao Watanabe, Marta Ferreira da Silva Rego, Vanessa Brandão Nardy, Shirlei Ferreira de Aguiar, Fabiana Cristina Pereira dos Santos, Alice Louize Nunes Queiroz, Bruno Tardelli Diniz Nunes, Lívia Carício Martins, Márcio Roberto Teixeira Nunes, Flávia Cristina da Silva Salles, Ingra Morales Claro, Jaqueline Goes de Jesus, Darlan da Silva Cândido, Cintia Marcela Fabbri, Claudia González, Lisseth Saéz, María Chen-Germán, Jaime Lagos Barrera, José Ernesto Ramírez-González, Josefina Campos, Noelia Morel Faller, Marta Eugenia Víquez Villalobos, Roberto Kaslin, Silvia Paola Salgado Cisneros, Flávia Figueira Aburjaile, Carolina Dourado Amaral, Danielle Bandeira Costa de Sousa Freire, Laura Nogueira Cruz, Daniel Mattos, Leandro Ferreira Lopes Landeira, Mariane Talon de Menezes, Ieda Maria Orioli, Ariane Coelho Ferraz, Daiane Teixeira de Oliveira, Alexandre Barbosa Reis, Renata Guerra de Sá Cota, Rafael dos Santos Bezerra, Melissa Barreto Falcão, Rodrigo Dias de Oliveira Carvalho, Edward C. Holmes, Tulio de Oliveira, José Lourenço, Luiz Carlos Junior Alcantara

**Affiliations:** 1 Laboratório Central de Saúde Pública do Estado de Minas Gerais, Fundação Ezequiel Dias, Belo Horizonte, Minas Gerais, Brazil; 2 Laboratório de Flavivírus, Instituto Oswaldo Cruz, Fundação Oswaldo Cruz, Rio de Janeiro, Rio de Janeiro, Brazil; 3 Laboratório de Ecologia de Doenças Transmissíveis na Amazônia, Instituto Leônidas e Maria Deane, Fiocruz, Manaus, Amazonas Brazil; 4 Laboratório Central de Saúde Pública do Estado de Mato Grosso do Sul, Campo Grande, Mato Grosso do Sul Brazil; 5 Laboratório Central de Saúde Pública Dr. Giovanni Cysneiros, Goiânia, Goiás Brazil; 6 Laboratório Central de Saúde Pública Professor Gonçalo Moniz, Salvador, Bahia Brazil; 7 Secretaria de Saúde do Estado da Bahia, Salvador, Bahia Brazil; 8 Laboratório Central de Saúde Pública Dr. Milton Bezerra Sobral, Recife, Pernambuco Brazil; 9 Laboratório Central de Saúde Pública do Estado de Mato Grosso, Cuiabá, Mato Grosso Brazil; 10 Laboratório Central de Saúde Pública do Distrito Federal, Brasília, Distrito Federal Brazil; 11 Coordenação Geral dos Laboratórios de Saúde Pública, Secretaria de Vigilância em Saúde, Ministério da Saúde, Brasília, Distrito Federal Brazil; 12 Organização Pan-Americana da Saúde/Organização Mundial da Saúde, Brasília, Distrito Federal Brazil; 13 Coordenação Geral das Arboviroses, Secretaria de Vigilância em Saúde/Ministério da Saúde, Brasília, Distrito Federal Brazil; 14 Fundação Hemocentro de Ribeirão Preto, Ribeirão Preto, São Paulo Brazil; 15 Gorgas Memorial Institute for Health Studies, Panama, Panama; 16 Universidade Federal da Bahia, Vitória da Conquista, Bahia Brazil; 17 Laboratorio Central de Salud Pública, Asunción, Paraguay; 18 Fundação Oswaldo Cruz, Bio-Manguinhos, Rio de Janeiro, Rio de Janeiro Brazil; 19 Instituto Evandro Chagas, Belém, Pará Brazil; 20 Instituto de Investigaciones en Ciencias de la Salud, San Lorenzo, Paraguay; 21 Secretaria de Estado de Saúde de Mato Grosso do Sul, Campo Grande, Mato Grosso do Sul Brazil; 22 Fundação Oswaldo Cruz, Campo Grande, Mato Grosso do Sul Brazil; 23 Secretaria de Saúde de Feira de Santana, Feira de Santana, Bahia Brazil; 24 Instituto de Ciências Biológicas, Universidade Federal de Minas Gerais, Belo Horizonte, Minas Gerais Brazil; 25 Secretaria de Saúde do Estado de Minas Gerais, Belo Horizonte, Minas Gerais Brazil; 26 Hospital das Forças Armadas, Brasília, Distrito Federal Brazil; 27 Secretaria de Vigilância em Saúde/Ministério da Saúde, Brasília, Distrito Federal Brazil; 28 Instituto de Higiene e Medicina Tropical, Universidade Nova de Lisboa, Lisboa, Portugal; 29 Marie Bashir Institute for Infectious Diseases and Biosecurity, School of Life and Environmental Sciences and School of Medical Sciences, University of Sydney, Sydney, NSW Australia; 30 KwaZulu-Natal Research Innovation and Sequencing Platform (KRISP), College of Health Sciences, University of KwaZulu-Natal, Durban, South Africa; 31 Department of Zoology, Peter Medawar Building, University of Oxford, Oxford, UK; 32 Universidade Estadual de Feira de Santana, Salvador, Bahia Brazil; 33 Instituto Gonçalo Moniz, Fundação Oswaldo Cruz, Salvador, Bahia Brazil; 34 Universidade de Brasília, Brasília, Distrito Federal Brazil; 35 Universidade Salvador, Salvador, Bahia Brazil; 36 Fundação Ezequiel Dias, Belo Horizonte, Minas Gerais Brazil; 37 Laboratório de Hantaviroses e Rickettsioses, Instituto Oswaldo Cruz, Fundação Oswaldo Cruz, Rio de Janeiro, Rio de Janeiro Brazil; 38 Faculdade de Medicina Veterinária, Universidade Federal de Minas Gerais, Belo Horizonte, Minas Gerais Brazil; 39 Laboratório Central de Saúde Pública do Estado do Paraná, Curitiba, Paraná Brazil; 40 Laboratório Central de Saúde Pública do Estado de Rondônia, Porto Velho, Rondônia Brazil; 41 Laboratório Central de Saúde Pública do Estado do Amazonas, Manaus, Amazonas Brazil; 42 Laboratório Central de Saúde Pública do Estado do Rio Grande do Norte, Natal, Rio Grande do Norte Brazil; 43 Laboratório Central de Saúde Pública Noel Nutels, Rio de Janeiro, Rio de Janeiro Brazil; 44 Instituto Adolfo Lutz, São Paulo, São Paulo Brazil; 45 Instituto de Medicina Tropical, Universidade de São Paulo, São Paulo, São Paulo Brazil; 46 Instituto Nacional de Enfermedades Virales Humanas Dr. Julio Maiztegui, Pergamino, Argentina; 47 Instituto de Salud Pública de Chile, Santiago, Chile; 48 Instituto de Diagnóstico y Referencia Epidemiológicos Dr. Manuel Martínez Báez, Ciudad de México, México; 49 Instituto Nacional de Enfermedades Infecciosas Dr Carlos G Malbrán, Buenos Aires, Argentina; 50 Ministerio de Salud Pública de Uruguay, Montevideo, Uruguay; 51 Instituto Costarricense de Investigación y Enseñanza em Nutrición y Salud, Tres Ríos, Costa Rica; 52 Instituto Nacional de Investigacion en Salud Publica Dr Leopoldo Izquieta Pérez, Guayaquil, Ecuador; 53 Universidade Federal de Pernambuco, Recife, Pernambuco Brazil; 54 Universidade Federal do Rio de Janeiro, Rio de Janeiro, Rio de Janeiro Brazil; 55 Universidade Federal de Ouro Preto, Ouro Preto, Minas Gerais Brazil

**Keywords:** Phylogenetics, Dengue virus

## Abstract

Brazil experienced a large dengue virus (DENV) epidemic in 2019, highlighting a continuous struggle with effective control and public health preparedness. Using Oxford Nanopore sequencing, we led field and classroom initiatives for the monitoring of DENV in Brazil, generating 227 novel genome sequences of DENV1-2 from 85 municipalities (2015–2019). This equated to an over 50% increase in the number of DENV genomes from Brazil available in public databases. Using both phylogenetic and epidemiological models we retrospectively reconstructed the recent transmission history of DENV1-2. Phylogenetic analysis revealed complex patterns of transmission, with both lineage co-circulation and replacement. We identified two lineages within the DENV2 BR-4 clade, for which we estimated the effective reproduction number and pattern of seasonality. Overall, the surveillance outputs and training initiative described here serve as a proof-of-concept for the utility of real-time portable sequencing for research and local capacity building in the genomic surveillance of emerging viruses.

## Abstract (web summary)

Here, the authors present results of the ZiBRA-2 project (https://www.zibra2project.org) which is an arbovirus surveillance project, across the Midwest of Brazil using a mobile genomics laboratory, combined with a genomic surveillance training program that targeted post-graduate students, laboratory technicians, and health practitioners in universities and laboratories.

## Introduction

Dengue virus (DENV) has spread extensively over the past decade and now poses a threat to about one-third of the global human population, primarily inhabitants of tropical and subtropical regions where mosquito vectors of the genus *Aedes* are widely distributed^1^. Increases in human mobility and population growth, unplanned urbanization, globalization, climate change, and unsuccessful vector control programs have contributed to DENV’s expansion, making it a major public health threat at a global scale^2^. Infection with the virus causes a wide spectrum of clinical manifestations, including asymptomatic or mild self-limiting disease (denoted dengue with and without warning signs), or life-threatening disease characterized by vascular leakage and hemorrhagic symptoms and classified as severe dengue^3^. DENV is a single-stranded, positive-sense RNA virus with a genome of ~11,000 kb belonging to the family *Flaviviridae* (genus *Flavivirus*). Its genome encodes a polyprotein that is post-translationally processed into three structural (capsid, pre-membrane or membrane, and envelope) and seven non-structural (NS1, NS2a, NS2b, NS3, NS4a, NS4b, and NS5) proteins^4^.

DENV is classified into four antigenically distinct and genetically related serotypes (DENV1-4), each of which harbor several phylogenetically defined genotypes not exceeding 6% nucleotide divergence and often with differing spatio-temporal distributions^5^. To date, 19 DENV genotypes have been identified, five in DENV1 (1I-V), six in DENV2 (2I-VI), and four in DENV3 (3I, 3II, 3III, 3V) and DENV4 (4I-IV)^6^. As serotype heterotypic infections are the highest risk factor for severe clinical outcome, the surveillance of circulating serotype diversity is pivotal to public health. Recent findings suggest that immunity generated by different genotypes of the same serotype may be more complex than previously assumed^7^, such that the contribution of genotype heterotypic infections to clinical outcomes and public health remains largely unknown.

DENV was successfully eradicated in many regions of South America during the mid-twentieth century^8^, with resurgence first reported in Brazil in the early 1980s in the northern state of Roraima^9^. Subsequently, the southern state of Rio de Janeiro played a pivotal role in the sequential resurgence of each serotype: DENV1 was reported to have been introduced there in 1986^10^, DENV2 in 1990 simultaneously with the first epidemic of severe dengue disease^11^, DENV3 in 2000^12^, and finally DENV4 in 2010^13^. Brazil can now be considered hyperendemic for dengue, reporting the highest number of dengue cases worldwide, with approximately 16 million notified infections since the 1980s^14^.

Different serotypes have caused unexpectedly large epidemics in Brazil over the past 20 years, with particularly problematic outbreaks in 2002, 2008, 2010 and 2012–2013^15^. In the years following the Zika virus epidemic (2017–2018) dengue reporting was surprisingly low^15^. However, the re-emergence of DENV2 in 2019 followed the report of a staggering 1,544,987 probable cases and 782 confirmed deaths (until epidemiological week – EW52 2019)^16^. Even with a long history of DENV circulation and recent experience with large outbreaks of other arthropod-borne viruses (arboviruses; e.g., Zika, chikungunya, yellow fever), Brazil continues to struggle with effective mosquito control and public health preparedness^17^. In contrast, over the past five years, the country has become a world leader in real-time genomic surveillance of arboviruses, contributing greatly to the amount of genetic data available in public databases, as well as for understanding the molecular evolution, spread, and persistence of such viruses (e.g., https://www.zibraproject.org and https://www.zibra2project.org). Despite this significant progress in research capacity, expertise on the methodologies involved in real-time genomic surveillance is absent from higher-education programs and is largely inaccessible to the majority of local researchers and public health workers^18^.

Herein, we describe a research and educational initiative that included genomic surveillance in the field and in the classroom under a two-week training program in 2019, during the post-Zika resurgence of DENV in Brazil. This initiative was attended by a large number of participants from public health and higher-education institutions from across Latin America. During this initiative we generated and analyzed 227 novel complete genome sequences of DENV1 and DENV2. Informed by a mix of public and the newly generated data, climate and epidemiological time series, we used phylogenetic and epidemiological models to infer the recent transmission history of these serotypes in Brazil. From these data we describe a complex dynamic history that supports previously proposed events of viral movement, replacement, and co-circulation. By targeting public health and higher-education institutions, generating and analyzing most of the data in real-time within the training program, we provide a proof-of-concept of the unique opportunities that portable sequencing technologies offer for local capacity building.

## Results

Dengue serotypes present temporal dynamics with an oscillatory behavior characterized by recurrent peak prevalence every 8-11 years^19^. Due to limitations in local testing capacity, inferring the relative prevalence of DENV1-4 with high spatio-temporal resolution in Brazil is often difficult. Nonetheless, our limited data (Supplementary Table 1) suggests that between 2015 and 2016 DENV1 was the dominant serotype in the Midwest, Northeast, and Southeast regions (Supplementary Fig. 1). Replacement in dominance by DENV2 took place in the Midwest region during 2017 and in the Southeast in subsequent years. Throughout the study period, DENV1 was the dominant serotype in the Northeast region. Although data-limited, these observations were supported by reports from other studies^20–22^, highlighting that the large epidemic of 2019 was dominated by serotypes DENV1 and DENV2. As we were aware of the dominance of DENV1 and DENV2 at the time of planning for the genomic surveillance initiative in 2019, data collection and analysis were focused on those two serotypes.

A total of 248 RT-qPCR positive samples for DENV1 (*n* = 62) and DENV2 (*n* = 186) were screened. Of the samples tested, 227 (DENV1 = 57, DENV2 = 170) contained sufficient DNA ( ≥ 2 ng/µL) to proceed to library preparation. For those positive samples, PCR cycle threshold (Ct) values were on average 22 (range: 15 to 35) for DENV1 and 23 (range: 16 to 35) for DENV2. Epidemiological details of the samples processed are provided in Supplementary Table 2. We used a portable nanopore sequencing approach^23^ to generate the complete genome sequences from the 227 viral samples. The resulting average coverage was 89% for DENV1 and 88% for DENV2. Sequencing statistics are detailed in Supplementary Table 3.

**Fig. 1:**
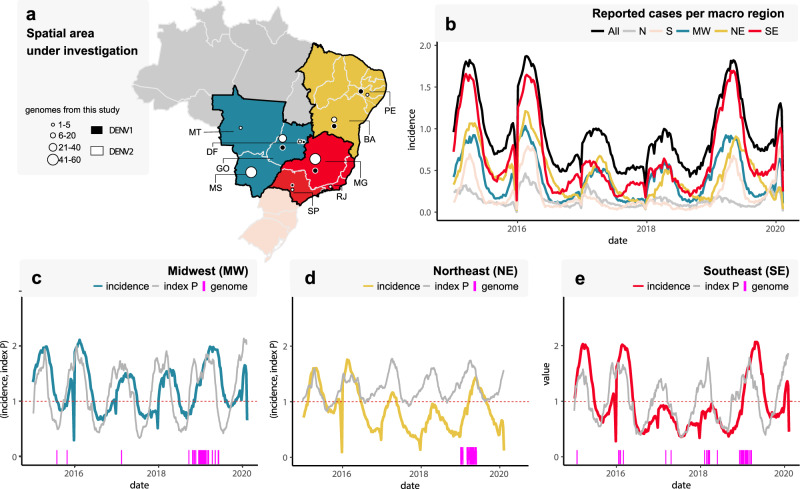
Spatial and temporal distribution of reported dengue cases in Brazil, 2015–2020. **a** Map of Brazil showing the number of DENV1 and DENV2 new sequences by region and state. DF = Federal District, BA = Bahia state, GO = Goiás state, MT = Mato Grosso state, MS = Mato Grosso do Sul state, MG = Minas Gerais state, PE = Pernambuco state, RJ = Rio de Janeiro state, SP = São Paulo state. The color and size of the circles indicates the number of new genomes generated in this study (black = DENV1, white = DENV2). **b** Weekly notified dengue cases normalized per 100 K individuals per region in 2015-2020 (until EW06). Epidemic curves are colored according to geographical macro region: SE = Southeast, NE = Northeast, MW = Midwest, N = North, S = South. **c**–**e** Time series of weekly reported cases normalized per 100 K individuals and daily mosquito-viral suitability measure (index P) in the three macro regions for which new sequences were generated: MW = Midwest (C), NE = Northeast (D), and SE = Southeast (E). The index P of each region is obtained using average climatic data for the three largest urban centers in each region. Purple bars highlight the dates of sample collection of the genomes generated here. **b**–**e** Incidence (cases per 100 K population) is presented in log10 for visual purposes. The initial map of Brazilian regions was obtained from the R package “get_brmap” (available at: https://rdrr.io/cran/brazilmaps/man/get_brmap.html). Source data are provided as a Source Data file.

DENV1 sequences covered the time period August 2015 to July 2019 in the Brazilian Federal District (*n* = 2) and five other states (Bahia = 19; Goiás = 10; Minas Gerais = 11; Pernambuco = 11; São Paulo = 4). For DENV2, samples were collected between January 2016 and August 2019 in the Federal District (*n* = 1) and eight other states (Bahia = 20; Goiás = 31; Minas Gerais = 47; Mato Grosso do Sul = 59; Mato Grosso = 2; Pernambuco = 3; Rio de Janeiro = 1; São Paulo = 3) (Fig. 1a). Three older DENV2 samples from the Goiás state, sampled during 2008, were also included. The median age of patients was 28 years (range: 4 to 78 years of age) for DENV1 and 37 years (range: 4 to 90 years of age) for DENV2. With respect to the clinical outcome, 77% of DENV1 (44/57) and 81% of DENV2 (138/170) samples were obtained from patients without alarming clinical signs, 44% of DENV1 (2/57) and 2% of DENV2 (4/170) corresponded to patients with severe dengue. Finally, 19% of DENV1 (11/57) and 17% of DENV2 (28/170) were obtained from fatal cases (Supplementary Table 2).

Weekly reported incidence (cases normalized per 100 K individuals) notified between 2015 and 2020 was aggregated into five Brazilian macro regions: North (N), South (S), Midwest (MW), Northeast (NE), and Southeast (SE) (Fig. 1b). Epidemic curves revealed three major outbreaks in all regions during early 2015, 2016, and mid-2019, with the Southeast region playing such a prominent role that its reported incidence was close to the overall incidence for Brazil (Fig. 1b, Supplementary Fig. 2). For the macro regions with new viral sequences (Midwest, Southeast and Northeast), we estimated transmission potential using a mosquito-viral suitability index. Since it is not possible to obtain representative temperature and humidity time series for the macro regions, we instead used climatic series from the three largest urban centers of each region. While the latter neglects climate variation within a macro region (Supplementary Fig. 3), the seasonal timing of reported cases was still well captured by the suitability index (Fig. 1c–e). The years 2015, 2016, and 2019 did not show particular increases in suitability, suggesting that the corresponding larger epidemics were not driven by particular climatic trends^20^, but rather by factors not accounted for by the index (e.g., sociodemographic, lack of herd immunity, change in circulating serotype or genotype, size of the mosquito population). There was also a clear decrease in reported cases across the regions during the aftermath of the Zika virus epidemic (2017–2018), a phenomenon also reported elsewhere^20,24,25^. In contrast, reporting of other arboviruses (e.g., chikungunya, Supplementary Fig. 4) saw increases in incidence during the same period. As such, we found no support for either climate driven reductions in transmission potential or arboviral reporting saturation that could explain the drop in DENV reporting between 2017 and 2018, suggesting that local herd immunity (serotype-specific or induced by Zika infection) may have played a role. Finally, apart from a few exceptions, the dates of the new sequences matched time periods of both high suitability and case reporting for all regions and were thus representative of epidemic periods (Fig. 1c–e).

Between 2015 and 2019, a total of 3,180 deaths attributed to DENV were reported in Brazil. We found a clear seasonal signal in weekly reported deaths that matched the seasonality of weekly reported cases and suitability for transmission (Supplementary Fig. 5). The year 2019 has been widely reported as experiencing a substantial increase in both the number of cases and deaths. Accordingly, we found the Midwest and Northeast regions had an increase in the absolute number of deaths in 2019 compared to previous years (Supplementary Fig. 5b and d). However, when the weekly (crude) case fatality rate (CFR = deaths/cases) was calculated there was no increase in the CFR during 2019 for any of the regions (Supplementary Fig. 5c, e, g). When aggregating the CFR between 2015 and 2019, we found the Midwest to have a higher CFR at 0.00084 (1.78 × 10^−05^–2.01 × 10^−03^ 95% range) compared to 0.0006 for the Northeast (0–0.0030) and 0.00037 for the Southeast regions (0-0.0010) (only the Midwest versus Southeast and Northeast comparisons were statistically different using a Wilcoxon test; p-values 5.76e-09 and 3.775e-05, respectively).

### DENV1 phylodynamics in Brazil, 2015 to 2019

**Fig. 2:**
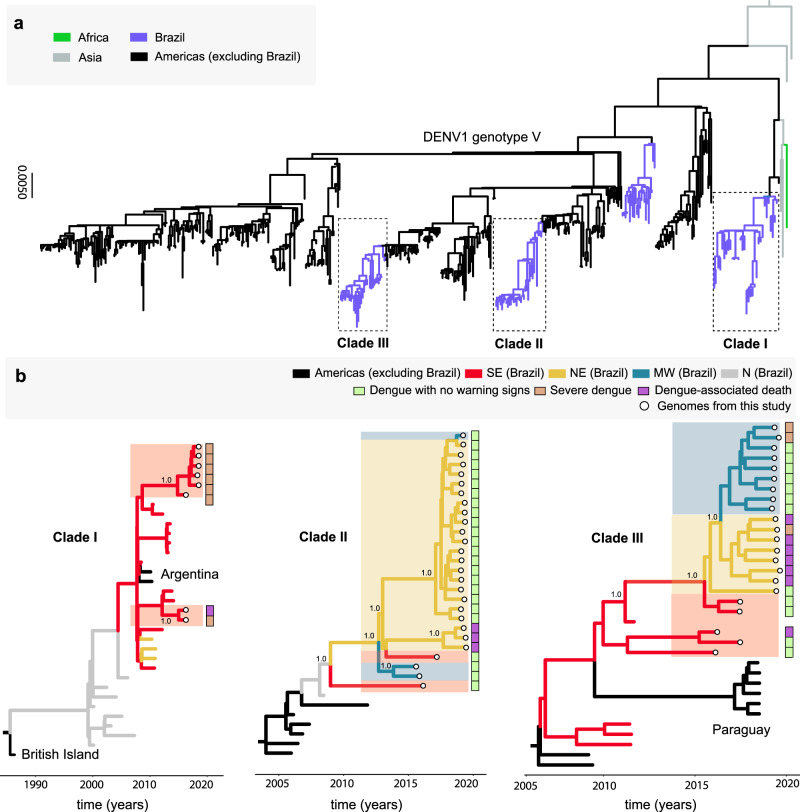
Phylogenetic analysis of DENV1-V in Brazil. **a** Maximum likelihood (ML) phylogenetic analysis of 57 complete genome sequences from DENV1 generated in this study plus 444 publicly available sequences from GenBank. The scale bar is in units of nucleotide substitutions per site (s/s) and the tree is mid-pointed rooted. Colors represent different sampling locations. **b** Time-scaled phylogeographic tree of Clade I (including eight new sequences plus 25 GenBank sequences), Clade II (including 27 new sequences plus seven GenBank sequences), and Clade III (including 22 new sequences plus 12 GenBank sequences). Colors represent different sampling locations (SE Brazil = Brazilian Southeast, NE Brazil = Brazilian Northeast, MW = Brazilian Midwest, N Brazil = Brazilian North). Tip circles represent the genome sequences generated in this study; colored sidebars represent the dengue clinical classification for each sequenced sample.

To explore the phylodynamics of DENV1, we combined our 57 newly generated sequences to those DENV1 genotype V (DENV1-V) genomes available on GenBank (*n* = 444). Phylogenetic analysis revealed that the novel isolates were organized into three distinct clades, named hereafter as clades I, II, III (Fig. 2a). Clade I appeared to have been replaced by clades II–III during 2019. The DENV resurgence in 2019 was characterized by the co-circulation of two viral lineages with no particular signatures suggesting clade-related advantages. In light of this, non-virological factors such as the level of susceptibility in the population remained a plausible driver of the expansion of both clades.

To investigate the evolution of clades I-III in more detail, we used smaller data sets derived from each clade individually, which only include sequences closely related to the newly Brazilian isolates (*n* = 33 for Clade I, *n* = 34 for Clade II and *n* = 34 for Clade III). An analysis of substitution rate constancy revealed a strong correlation between the sampling time and the root-to-tip divergence in all three data sets (Supplementary Fig. 6), allowing the use of molecular clock models to infer evolutionary parameters. Phylogeographic analyses of clade I (Fig. 2b) clustered the new sequences into a single well-supported monophyletic sub-clade (posterior probability support, PPS = 1.0), including isolates sampled between 2000 and 2018. New sequences in this clade were mainly from severe dengue cases registered in the SE region. The time to the most recent ancestor (tMRCA) of all Brazilian sequences was estimated to be between June 1998 to February 2000, and this common ancestor likely originated in the North region (PPS = 1.0), after a dispersion of a virus from the British Virgin Islands (PPS = 1.0). Viruses from this clade spread from the North region towards the Southeast and then the Northeast, as indicated by isolates from the Pernambuco (PE) state (represented by JX669461, JX669465, and JX669464). The tMRCA of all isolates from the Southeast and Northeast regions in this clade was estimated to be between May 2006 to February 2008.

Similarly, an analysis of clade II (Fig. 2b) revealed a single well-supported monophyletic group (PPS = 1.0), including isolates from the Southeast, Midwest, and Northeast regions sampled between 2015 and 2019. The majority of the new sequences were from mild dengue cases, although three isolates were recovered from fatal cases in the Northeast. The tMRCA of this group was dated to between August 2007 and May 2010, with a likely origin in the North region. However, as the PPS value was low (0.39) the place of origin remains uncertain. After its introduction into the North region, viruses from this lineage appear to have moved towards the Southeast, Midwest, and Northeast. Notably, the clade appears to have persisted locally after its introduction in the Northeast region between July 2011 to June 2014, and from there a second introduction into the Midwest region may have occurred, as suggested by a single isolate (OPAS134) sampled in 2019.

Finally, clade III (Fig. 2b) also formed a single supported monophyletic group (PPS = 0.82) that contained sequences from the Southeast, Northeast, and Midwest regions sampled between 2011 and 2019. Among our new isolates, six were from fatal cases reported in the Northeast. We estimated the age of this sub-clade to be between October 2009 to August 2011, with a most likely origin in the Southeast region (PPS = 0.99). Since its introduction, the clade has circulated in the Southeast, from where it has later dispersed to Paraguay. The Southeast region has also seemingly seeded outbreaks into the Northeast (PPS = 0.88) between February 2015 and September 2017, and subsequently towards the Midwest region between June 2016 to April 2018.

### DENV2 phylodynamics in Brazil, 2016 to 2019

**Fig. 3:**
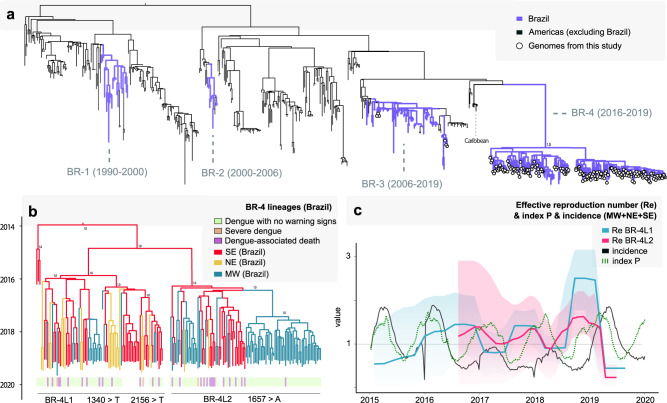
Phylogenetic analysis of DENV2-III in Brazil. **a** A maximum likelihood tree was inferred using 170 complete genome sequences from DENV2 generated in this study and 450 sequences publicly available, retrieved from GenBank. The scale bar is in units of nucleotide substitutions per site (s/s) and mid-point rooted. Tip circles represent the genome sequences generated in this study. **b** Time-scaled phylogeographic tree of DENV2 BR-4, including 164 new DENV2 sequences generated here and 17 publicly available data from the 2019 outbreak in Brazil^22^. Sequences are colored according to sampling location. Sidebars represent the dengue clinical classification for each sequenced sample. **c** Temporal fluctuation of the effective reproduction number (R_e_) of the for DENV2 BR-4L1 (blue) and BR-4L2 (magenta) estimated using the Bayesian birth-death approach. Black line is the total weekly incidence of dengue between 2015 and 2020 (until EW06), and the dotted green line is the index P (incidence is summed and index P is averaged over the three macro regions for which new sequences were generated: MW = Midwest, NE = Northeast, and SE = Southeast). Incidence (cases per 100 K population) is presented in log10 for visual purposes.

To explore the phylodynamics of DENV2 between 2016 and 2019, we performed a phylogenetic analysis of the 170 newly generated sequences plus 450 complete genome sequences of DENV2-III available on GenBank (Fig. 3). This analysis revealed four different clades (termed hereafter BR-1 to BR-4 clades)^22^. Notably, BR-1 contained Brazilian sequences sampled from 1990–2000, BR-2 from 2000–2006, and BR-3 from 2006–2019. The latter included six of our new isolates (34%, 6/170) collected during previous outbreaks in 2008 (Goiás = 3) and 2016 (São Paulo = 3). Finally, BR-4 contained the other 164 new sequences (97%, 164/170), sampled between 2016 and 2019. This phylogenetic pattern suggested that between 2016 and 2019, DENV2 circulated in these Brazilian regions with a succession of different viral clades. In particular, with BR-3 preceding BR-4, and with older clades (BR-1, BR-2) either not being sampled in the most recent time-points or having experienced local extinction. These results were consistent with a recent study highlighting the role of the Caribbean region in the spread of the BR-4 clade into Brazil^20,22^ (Fig. 3a).

Given the substantial number of novel sequences, we examined the BR-4 clade in more detail (Fig. 3b) (and a linear regression of root-to-tip genetic distance against sampling date revealed sufficient temporal signal, *r*^2^ = 0.60; Supplementary Fig. 7). The BR-4 clade (PPS = 1.0) included 181 isolates from the Southeast, Northeast, and Midwest regions, 92% (167/181) of which were sampled during the 2019 outbreak and 88% (14/181) sampled between 2016 and 2018. Of these, 133 were from dengue without warning signs, while three isolates were recovered from severe dengue, and 28 were from fatal dengue cases. We identified two distinct BR-4 lineages circulating between 2017 and 2019, which we termed BR-4L1 and BR-4L2 (Fig. 3b). Both lineages contained sequences from the Northeast, Southeast, and Midwest regions. The tMRCA of BR-4L1 was estimated to be between September 2014 and June 2016, while the tMRCA of BR-4L2 was dated between March 2015 to November 2016. BR-4L2 contained a monophyletic cluster of isolates from the Midwest sampled between 2018 and 2019. We also observed that the other isolates from the Northeast and Midwest regions were intermixed throughout both lineages, indicating multiple introductions of DENV2 over time. Similar to recent studies^22^, we found the tMRCA of BR-4 to be between November 2013 and May 2015, likely in the Southeast (PPS = 0.99), from where the virus dispersed towards the Northeast and Midwest regions. Minas Gerais state, located in the Southeast, seems to have played an important role as source location, since sequences from this region (from 2016) fell close to the root of the clade (Fig. 3b).

We identified 34 single nucleotide variants between the BR-4L1 and BR-4L2 lineages, only three of which resulted in amino acid substitutions. Isolates of the BR-4L1 acquired one unique amino acid substitution A447V (ENV protein), while only a few isolates of this lineage had a second amino acid substitution K719I (NS5 protein). All isolates of BR-4L2 acquired one unique amino acid substitution V553I (NS5 protein) (Supplementary Table 4).

We used a birth-death skyline (BDSKY) model to estimate the effective reproduction number (R_e_) of BR-4L1 and BR-4L2 (Fig. 3c). This provided evidence for three significant seasonal oscillations in R_e_ (although with wide credible intervals), consistent but generally preceding the time windows of reported outbreaks between 2016 and 2019 (Fig. 1c–e). Mosquito-viral suitability presented the same general patterns, but the timing of its oscillations was in between that of R_e_ and incidence (Fig. 3c). In general, our estimates of R_e_ for both lineages peaked at the end of each year, decreasing and remaining below 1 temporarily at the start of each following year (although again, with wide credible intervals). Notably, the time period with the largest R_e_ for both lineages at the end of 2018 and preceding the large epidemic of 2019 (in excess of 2.5 for BR-4L1 and 1.5 for BR-4L2) did not coincide with similar increases in suitability, once again suggesting that climate-related factors were not the drivers of epidemic success.

## Discussion

More than 16 million cases of dengue disease have been notified since the early 1980s in Brazil^9,14^. Previous studies have explored the evolution of DENV1-4 in the Americas, mainly focusing on a restricted range of countries using partial genome sequences^26–29^. To obtain a better understanding of DENV evolution in Brazil, in particular during its resurgence in 2019 when DENV1-2 dominated reported cases, we generated 227 new complete genome sequences of both serotypes using portable sequencing. Importantly, more than three quarters of the new sequences were processed and analyzed during a Nanopore-based genome sequencing training and surveillance program that took place in Belo Horizonte, Minas Gerais state, in 2019. The new 227 sequences generated corresponded to 55% (57/104) of DENV1-V, and 60% (170/285) of DENV2-III Brazilian complete genomes that are currently available in public databases (Supplementary Fig. 8). This highlights the large contribution of the training initiative, but also the current shortage of complete genome data for both serotypes. There is clearly a need for continued funding for genomic surveillance which, as shown here, can contribute to a better understanding of the introduction, spread, and persistence of dengue viruses in Brazil during epidemics with significant public health impact.

Time series of reported cases between 2015 and 2019 showed the typical yearly seasonal patterns of dengue transmission. Reporting was low in 2017 and 2018, coinciding with the post-epidemic period of the Zika virus in Brazil. Such trends have also been reported in other countries^20,24^ and are speculated to be driven by transient cross-protection from exposure to Zika, and/or temporary saturation or changes in surveillance^20,30^. When comparing to reported cases of chikungunya virus and estimated mosquito-viral suitability in the same period, we found no evidence of changes in capacity for arboviral surveillance or climate driven low transmission potential in favor of other mechanisms (e.g., Zika cross-immunity). In contrast to this period of low circulation, there were three particularly large DENV epidemics: in 2015 and 2016 when DENV1 was dominant across all regions, and in 2019 when DENV1 was dominant in the Northeast but DENV2 was dominant in the Midwest and Southeast regions. Due to the increased likelihood of secondary infections, serotype replacement is often associated with measurable changes in the clinical spectrum of reported cases, with increases in both disease severity and number of deaths^11,29,31^. While we describe an increase in absolute case and death numbers in 2019, the emergence of DENV2 in the Southeast and Midwest regions was not associated with a significant increase in the case fatality rate compared to previous years.

Our newly generated DENV1 sequences were classified as genotype V and formed three distinct clades (I–III). This supports previous reports suggesting that such clades were responsible for the latest DENV1 outbreaks in Brazil^27,28,31,32^. Within clade I, only eight of the new sequences were sampled between 2016–2018, with many isolates preceding 2015. The shortage of genomic data in the intermediate years severely hampered our capacity to draw further conclusions, such as the possibility of a temporary lineage replacement event. In contrast, most of the new sequences within clades II–III were sampled in 2019, supporting the co-circulation of two DENV1 lineages in recent epidemics - a phenomenon often described in DENV epidemics^27,28,31,32^. Viruses from the three clades have been identified in both the Southeast and Northeast regions, although the most recent ones only appeared in the Southeast, while viruses from clades II-III were present in the Northeast and Midwest regions. While this suggested some structure in spatio-temporal circulation, we could not ascertain if it was simply to biased sampling. Time estimates of the tMRCA of the new Brazilian isolates (2015–2019) were between May 2006 and February 2008 for clade I, between August 2007 and May 2010 for clade II, and between October 2009 to August 2011 for clade III. Such large ranges are likely reflecting the lack of genomic data during this period, reinforcing the need for a more effective surveillance in Brazil.

All new DENV2 complete genome sequences belong to genotype III, which has been found in previous epidemics in Brazil^22,33^. Our results are in line with reports of three different lineages causing outbreaks in Brazil since 1990^32,33^, and support a recent introduction of DENV2-III^20,22^. Viruses from this genotype grouped in four different clades (BR-1 to BR-4) with apparent replacement over time. Specifically, the oldest lineage BR-1, including isolates from 1990–2000, was replaced by BR-2 comprising sequences from 2000–2006, itself subsequently replaced by BR-3 containing isolates from 2006–2019. Finally, BR-3 was replaced by BR-4, containing sequences sampled between 2016–2019, some closely related to Caribbean isolates sampled in 2005. In a similar manner to DENV1, DENV2 BR-3 and BR-4 isolates from 2019 demonstrated the co-circulation of at least two different lineages in recent years^22^. The phylogenetic relationship to Caribbean sequences suggested a possible origin in this region, although there is a large temporal gap between the sampling of the Caribbean sequences from 2005 and the early Brazilian sequences from 2016, again highlighting the need for more genomic surveillance in Brazil. The tMRCA of BR-4L1 was estimated between September 2014 and June 2016 and between March 2015 and November 2016 for BR-4L2, coinciding with the emergence and spread of the Zika^34^ and Chikungunya^35^ viruses, and a high incidence of dengue in Brazil. Six years after introduction, the lineages continue to circulate in the Southeast region and were present in the most recent large epidemic of 2019. From the Southeast, dispersion was towards the Northeast and Midwest regions, with multiple independent introductions identified.

Analysis of the 181 isolates from three macro regions (Northeast, Midwest and Southeast) allowed us to estimate the emergence of DENV2 BR-4 in the Southeast between November 2013 and May 2015, supporting previous reports^20,22^. After its introduction, BR-4 circulated as two distinct lineages (BR-4L1 and BR-4L2). We observed three single nucleotide variants among the lineages that resulted in amino acid substitutions: A447V and K719I were identified in the BR-4L1, while V553I was identified in BR-4L2. A447V (ENV protein) and V553I (NS5 protein) appear to be conservative changes due to the interchangeable character for the respective amino acids^36^. In contrast, K719I in the NS5 protein has changed from a negatively charged to an aliphatic amino acid^36^. Further studies are required to elucidate the impact of these variants on structure and function of the associated proteins, and any potential role in both viral pathogenesis and fitness.

Our retrospective reconstruction of the recent transmission history of DENV1 and DENV2 revealed that the Southeast and North regions of Brazil were key to dispersal in Brazil. This is in line with studies that have highlighted both regions as important hubs for introduction and dispersion in the country, not only of DENV^9–12,27,31,33^, but also for yellow fever virus^37^. By combining genetic and epidemiological models we showed that the establishment and the co-circulation of the BR-4L1 and BR-4L2 lineages of DENV2 in several Brazilian regions occurred during a time window of sustained transmission potential measured by estimates of R_e_ and mosquito-viral suitability. These results are consistent with sufficient ecological suitability for the virus’s main vectors (*Aedes* spp.) and insufficient population-level herd-immunity, supporting the expectation of continuing endemic circulation of these dengue viruses in Brazil.

The new genomic data presented here were generated using portable sequencing tools in a field surveillance initiative (ZiBRA-2 project) and a genomic surveillance training program. We present a range of research outputs describing the recent history and genomic epidemiology of DENV1 and DENV2 in Brazil during the resurgence of this virus in 2019: this both corroborates previous studies and greatly increases the number of public viral genome sequences available for analysis. We also identified gaps in existing genomic data, that curtailed definite conclusions on key points of the recent history of DENV in Brazil. Importantly, epidemiological and genomic data was analyzed in real-time during the training program and subsequently during online sessions, and the participants attending the training program made a significant contribution to the research outputs generated. We call for continued funding of similar field and classroom genomic surveillance initiatives. These have the potential to build local capacity in the field of genomic surveillance and in doing so advance our understanding on the population biology of circulating arboviruses and other emerging pathogens.

## Methods

### Ethics statement

This project was reviewed and approved by the Pan American Health Organization Ethics Review Committee (PAHOERC) (Ref. No. PAHO-2016-08-0029) and the Oswaldo Cruz Foundation Ethics Committee (CAAE: 90249218.6.1001.5248). The availability of these samples for research purposes during outbreaks of national concern is allowed to the terms of the 510/2016 Resolution of the National Ethical Committee for Research – Brazilian Ministry of Health (CONEP - Comissão Nacional de Ética em Pesquisa, Ministério da Saúde), that authorize, without the necessity of an informed consent, the use of clinical samples collected in the Brazilian Central Public Health Laboratories to accelerate knowledge building and contribute to surveillance and outbreak response. The samples processed in this study were obtained anonymously from material exceeding the routine diagnosis of arboviruses in Brazilian public health laboratories that belong to the public network within BrMoH.

### Field genomic surveillance with a mobile laboratory

In May 2019 we implemented an arbovirus surveillance project that took place across the Midwest of Brazil using a mobile genomics laboratory (Supplementary Fig. 9). This Brazilian-driven initiative, known as the ZiBRA-2 project, was supported by the BrMoH (https://www.zibra2project.org).

### Classroom genomic surveillance in a training program

In August 2019 a genomic surveillance training program organized by PAHO and BrMoH took place in Belo Horizonte (Minas Gerais state) under the title “Nanopore-based genome sequencing technology for temporal investigation and epidemiology of dengue outbreak: training, research, surveillance, and scientific dissemination”. The syllabus included practical and theoretical courses on a variety of subjects related to arbovirus research and surveillance, including mobile sequencing technologies, bioinformatics, phylogenetics, epidemiological modeling, and field epidemiology and entomology. The course was taught by experienced researchers from national and international institutions, such as the University of Oxford (United Kingdom), University of KwaZulu-Natal (South Africa), University Nova de Lisboa (Portugal), Sechenov First Moscow State Medical University (Russia), Oswaldo Cruz Foundation (Brazil), Federal University of Minas Gerais (Brazil), Federal University of Rio de Janeiro (Brazil), Federal University of Pernambuco (Brazil), University of São Paulo (Brazil), University of Brasilia (Brazil), State University of Feira de Santana (Brazil), and University of Salvador (Brazil). The course had 62 students from 34 national and international institutions (age range of participants between 25–50). In addition to post-graduate students, course participants included laboratory technicians and health practitioners in universities and laboratories from several institutions responsible for laboratory-based surveillance of emerging and reemerging diseases, such as the Central Public Health Laboratories of the Brazilian states from the BrMoH’s network and public health laboratories from Paraguay, Argentina, Panama, Chile, Mexico, Uruguay, Costa Rica, and Ecuador. The event targeted post-graduate students, laboratory technicians, and health practitioners in universities and laboratories across the Americas and was based on the principles of Responsible Research and Innovation (RRI)^38^. Details on the program can be found in Supplementary Text File 1.

### Sample collection and molecular diagnostic assays

Clinical samples from patients with suspected DENV infection were obtained for routine diagnostic purposes at local health services in different Brazilian municipalities. These samples were sent for molecular diagnosis to the respective local Central Laboratory of Public Health (LACEN) from the Brazilian Federal District (DF) and from the states of Bahia (BA), Goiás (GO), Mato Grosso (MT), Mato Grosso do Sul (MS), Minas Gerais (MG), Pernambuco (PE), and Rio de Janeiro (RJ). These states had some of the highest registered burdens during the 2019 DENV resurgence according to the protocol established by the BrMoH. Samples processed from the state of São Paulo (SP) were collected by the Blood Center of Ribeirão Preto from volunteer blood donors eligible for blood donation and who reported adverse effects up to 14 days after donation.

Viral RNA was extracted from all clinical samples using the QIAmp Viral RNA Mini Kit (Qiagen) and tested by RT-qPCR for detection of DENV1-4. Selected samples with previous positive diagnostic results for DENV1-2 were processed in two steps: (1) 73 samples from the states of Goiás, Mato Grosso do Sul, and Mato Grosso were processed during the ZiBRA-2 project, (2) 175 samples from the Brazilian Federal District and Bahia, Goiás, Minas Gerais, Pernambuco, Rio de Janeiro, and São Paulo states were processed during the training program (both initiatives described in the section above). Samples from the 2019 outbreak, as well as available samples from previous epidemic waves in 2008 and between 2015-2018, were included for diagnostic screening.

### cDNA synthesis and whole genome sequencing using the MinION

Samples were selected for sequencing based on the Ct value (≤35) and availability of epidemiological metadata, such as date of symptom onset, date of sample collection, sex, age, municipality of residence, symptoms, and disease classification. For complementary DNA synthesis, the SuperScript IV Reverse Transcriptase kit (Invitrogen) was used following the manufacturer’s instructions. The cDNA generated was subjected to sequencing multiplex PCR (35-cycles) using Q5 High Fidelity Hot-Start DNA Polymerase (NEB) and a set of specific primers designed by the CADDE project (https://www.caddecentre.org/) for sequencing the complete genomes of DENV1 and DENV2^39^ (Supplementary Table 5).

Amplicons were purified using 1x AMPure XP Beads (Beckman Coulter) and quantified on a Qubit 3.0 fluorimeter (Thermofisher Scientific) using Qubit™ dsDNA HS Assay Kit (Thermofisher Scientific). Of the 248 samples, 227 contained sufficient DNA (≥2 ng/µL) to proceed to library preparation. DNA library preparation was performed using the Ligation Sequencing Kit (Oxford Nanopore Technologies) and the Native Barcoding Kit (NBD103, Oxford Nanopore Technologies)^23^. Sequencing libraries were generated from the barcoded products using the Genomic DNA Sequencing Kit SQK-MAP007/SQK-LSK208 (Oxford Nanopore Technologies) and loaded into a R9.4 flow cell (Oxford Nanopore Technologies). In each sequencing run we used negative controls to prevent and check for possible contamination with less than 2% mean coverage.

### Generation of consensus sequences

Raw files were basecalled using Guppy v3.4.5 and barcode demultiplexing was performed using qcat. Consensus sequences were generated by de novo assembling using Genome Detective (https://www.genomedetective.com/)^40^. Briefly, Genome Detective use DIAMOND to identify and classify candidate viral reads in broad taxonomic units, using the viral subset of the Swissprot UniRef90 protein database. Candidate reads are next assigned to candidate reference sequences using NCBI blastn and aligned using AGA (Annotated Genome Aligner) and MAFFT. Final contigs and consensus sequence are then available as FASTA file. More detail about Genome Detective can be found in^40^. The new sequences reported in this study (DENV1 *n* = 57 and DENV2 *n* = 170), were initially submitted to a genotyping analysis using the arbovirus phylogenetic subtyping tool, available at http://genomedetective.com/app/typingtool/dengue; this confirmed that the newly genomes belonged to the genotypes DENV1-V and DENV2-III, respectively.

### Phylogenetic analysis

DENV genotyping was performed using the Dengue Virus Typing Tool (https://www.genomedetective.com/app/typingtool/dengue/)^6^. To investigate the evolution and population dynamics of DENV1-2 in different Brazilian regions, the DENV1 (*n* = 57) and DENV2 (*n* = 170) complete genome sequences generated in this study were combined with globally sampled and publicly available complete genome sequences from DENV1 genotype V (DENV1-V = 444) and DENV2 genotype III (DENV2-III = 450) as these represent the dominant genotypes in the Americas. The latter were retrieved from NCBI up to November 2019. We also included 17 recently published of the outbreaks in the Brazilian Southeast region^25^. Sequences without sampling date and location were excluded, as were sequences covering less than 50% of the viral genome.

Sequence alignment was performed using MAFFT^41^ and manually curated to remove artifacts using Aliview^42^. Maximum Likelihood (ML) phylogenetic trees were estimated using IQ-TREE^43^ under the GTR nucleotide substitution model, which was inferred as the best-fit model by the ModelFinder application implemented in IQ-TREE^44^. The robustness of the tree topology was determined using 1,000 bootstrap replicates, and the presence of temporal signal was evaluated in TempEst^45^ through a regression of root-to-tip genetic distances against sampling time. Time-scaled phylogenetic trees were inferred using the BEAST package^46^. We employed a stringent model selection analysis using both path-sampling (PS) and stepping stone (SS) procedures to estimate the most appropriate molecular clock model for the Bayesian phylogenetic analysis^47^. The uncorrelated relaxed molecular clock model was chosen as indicated by estimating marginal likelihoods, also employing the codon based SRD06 model of nucleotide substitution and the non-parametric Bayesian Skyline coalescent model. A discrete phylogeographic model^48^ was used to reconstruct the virus spatial diffusion across the sampling locations. Discrete locations were initially defined as the country of sampling. However, a different resolution was applied according to sampling availability. Phylogeographic analyses were then performed by applying an asymmetric model of location transitioning coupled with the Bayesian Stochastic Search Variable Selection (BSSVS) procedure. Markov Chain Monte Carlo (MCMC) were run in duplicate for 100 million iterations to ensure stationarity and an adequate effective sample size (ESS) of >200. Maximum clade trees were summarized using TreeAnnotator after discarding 10% as burn-in and visualized using FigTree v1.4.4.

### Epidemiological data and integration with genomic data

Data of weekly notified and laboratory confirmed cases of infection by DENV in Brazil during 2015 to 2019, as well as monthly fatal cases with confirmed dengue infection, were supplied by the BrMoH. A mosquito-viral suitability measure (index P) was estimated using the MVSE R-package^49^. The index P measures the reproductive (transmission) potential of a single adult female mosquito in a completely susceptible host population and is informed by local temperature and humidity time trends. We used daily climatic data from the three largest cities of each macro region for which new sequences were generated (Midwest: Goiânia, Brasília, Campo Grande; Southeast: São Paulo, Rio de Janeiro, Belo Horizonte; Northeast: Salvador, Recife, Fortaleza), with data obtained from openweathermap.org.

### Estimating R_e_ from genetic sequences

We used birth-death models implemented in BEASTv2.4^50^ to estimate the R_e_. In this model, each infection may transmit at a rate *λ* and will become non-infectious at a rate *δ*. Upon becoming infected, each individual is sampled with a probability *s*^*s*^. The model enables the piecewise estimation of R_e_, *δ*, and *s* through time. We assumed sampling proportion *s* to be constant over time. Relaxing this assumption to allow the parameter *s* to be zero for the periods when no sequence data was available resulted in similar trends for R_e_, with wider Bayesian credible intervals. The rate *δ* was modeled using a lognormal prior with a mean of 14 days and a standard deviation of 0.5, which roughly corresponds to the sum of the intrinsic and extrinsic incubation period of dengue virus. The BDSKY analysis was run for an independent MCMC chains of >100 steps, with parameters and trees being sampled once every 10,000 steps. After removal of 10% burn in, sampled parameters were combined using LogCombiner.

### Reporting summary

Further information on research design is available in the Nature Research Reporting Summary linked to this article.

## Supplementary information

Supplementary Information

Reporting Summary

## Data Availability

Newly generated DENV1 and DENV2 sequences have been deposited in GenBank under accession numbers MT929528-MT929754. More detail about the sequences generated can be found at Supplementary Table 3. Source data are provided with this paper. Alignments, tree files and epidemiological data can be found at: 10.5281/zenodo.4604002. Source data are provided with this paper.

## Source data

Source Data
