# Supplementary material for: Field and classroom initiatives for portable sequence-based monitoring of dengue virus in Brazil
Source: Nat Commun. 2021 Apr 16;12:2296. doi: 10.1038/s41467-021-22607-0 (PMC8052316; doi:10.1038/s41467-021-22607-0)
Supplement: Supplementary file 1 — Supplementary Information [file 41467_2021_22607_MOESM1_ESM.docx]

**SUPPLEMENTARY INFORMATION**

**Field and classroom initiatives for portable sequence-based monitoring of dengue virus in Brazil**

Talita Émile Ribeiro Adelino^1^^, Marta Giovanetti^2^*^, Vagner Fonseca^2^^, Joilson Xavier^2^, Álvaro Salgado de Abreu^2^, Valdinete Alves do Nascimento^3^, Luiz Henrique Ferraz Demarchi^4^, Marluce Aparecida Assunção Oliveira^1^, Vinícius Lemes da Silva^5^, Arabela Leal e Silva de Mello^6^, Gabriel Muricy Cunha^7^, Roselene Hans Santos^8^, Elaine Cristina de Oliveira^9^, Jorge Antônio Chamon Júnior^10^, Felipe Campos de Melo Iani^1^, Ana Maria Bispo de Filippis^2^, André Luiz de Abreu^11^, Ronaldo de Jesus^11^, Carlos Frederico Campelo de Albuquerque^12^, Jairo Mendez Rico^12^, Rodrigo Fabiano do Carmo Said^12^, Joscélio Aguiar Silva^13^, Noely Fabiana Oliveira de Moura^13^, Priscila Leite^13^, Lívia Carla Vinhal Frutuoso^13^, Simone Kashima Haddad^14^, Alexander Martínez^15^, Fernanda Khouri Barreto^16^, Cynthia Carolina Vazquez^17^, Rivaldo Venâncio da Cunha^18^, Emerson Luiz Lima Araújo^11^, Stephane Fraga de Oliveira Tosta^2^, Allison de Araújo Fabri^2^, Flávia Löwen Levy Chalhoub^2^, Poliana da Silva Lemos^19^, Fernanda de Bruycker-Nogueira^2^, Gislene Garcia de Castro Lichs^4^, Marina Castilhos Souza Umaki Zardin^4^, Fátima María Cardozo Segovia^20^, Crhistinne Cavalheiro Maymone Gonçalves^21^, Zoraida Del Carmen Fernandez Grillo^22^, Svetoslav Nanev Slavov^14^, Luiz Augusto Pereira^5^, Ana Flávia Mendonça^5^, Felicidade Mota Pereira^6^, Jurandy Júnior Ferraz de Magalhães^8^, Agenor de Castro Moreira dos Santos Júnior^10^, Maricélia Maia de Lima^23^, Rita Maria Ribeiro Nogueira^2^, Aristóteles Góes-Neto^24^, Vasco Ariston de Carvalho Azevedo^24^, Dario Brock Ramalho^25^, Wanderson Kleber Oliveira^26^, Eduardo Marques Macario^27^, Arnaldo Correia de Medeiros^27^, Victor Pimentel^28^, Latin American Genomic Surveillance Arboviral Network^29^, Edward C Holmes^30^, Tulio de Oliveira^31^, José Lourenço^32*^, Luiz Carlos Junior Alcantara^2*^

^1^Laboratório Central de Saúde Pública do Estado de Minas Gerais, Fundação Ezequiel Dias, Belo Horizonte, Minas Gerais, Brazil; ^2^Laboratório de Flavivírus, Instituto Oswaldo Cruz, Fundação Oswaldo Cruz, Rio de Janeiro, Rio de Janeiro, Brazil; ^3^Laboratório de Ecologia de Doenças Transmissíveis na Amazônia, Instituto Leônidas e Maria Deane, Fiocruz, Manaus, Amazonas, Brazil; ^4^Laboratório Central de Saúde Pública do Estado de Mato Grosso do Sul, Campo Grande, Mato Grosso do Sul, Brazil; ^5^Laboratório Central de Saúde Pública Dr. Giovanni Cysneiros, Goiânia, Goiás, Brazil; ^6^Laboratório Central de Saúde Pública Professor Gonçalo Moniz, Salvador, Bahia, Brazil; ^7^Secretaria de Saúde do Estado da Bahia, Salvador, Bahia, Brazil; ^8^Laboratório Central de Saúde Pública Dr. Milton Bezerra Sobral, Recife, Pernambuco, Brazil; ^9^Laboratório Central de Saúde Pública do Estado de Mato Grosso, Cuiabá, Mato Grosso, Brazil; ^10^Laboratório Central de Saúde Pública do Distrito Federal, Brasília, Distrito Federal, Brazil; ^11^Coordenação Geral dos Laboratórios de Saúde Pública, Secretaria de Vigilância em Saúde, Ministério da Saúde, Brasília, Distrito Federal, Brazil; ^12^Organização Pan-Americana da Saúde/Organização Mundial da Saúde, Brasília, Distrito Federal, Brazil; ^13^Coordenação Geral das Arboviroses, Secretaria de Vigilância em Saúde/Ministério da Saúde, Brasília, Distrito Federal, Brazil; ^14^Fundação Hemocentro de Ribeirão Preto, Ribeirão Preto, São Paulo, Brazil; ^15^Gorgas Memorial Institute for Health Studies, Panama, Panama; ^16^Universidade Federal da Bahia, Vitória da Conquista, Bahia, Brazil; ^17^Laboratorio Central de Salud Pública, Asunción, Paraguay; ^18^Fundação Oswaldo Cruz, Bio-Manguinhos, Rio de Janeiro, Rio de Janeiro, Brazil; ^19^Instituto Evandro Chagas, Belém, Pará, Brazil; ^20^Instituto de Investigaciones en Ciencias de la Salud, San Lorenzo, Paraguay; ^21^Secretaria de Estado de Saúde de Mato Grosso do Sul, Campo Grande, Mato Grosso do Sul, Brazil; ^22^Fundação Oswaldo Cruz, Campo Grande, Mato Grosso do Sul, Brazil; ^23^Secretaria de Saúde de Feira de Santana, Feira de Santana, Bahia, Brazil; ^24^Instituto de Ciências Biológicas, Universidade Federal de Minas Gerais, Belo Horizonte, Minas Gerais, Brazil; ^25^Secretaria de Saúde do Estado de Minas Gerais, Belo Horizonte, Minas Gerais, Brazil; ^26^Hospital das Forças Armadas, Brasília, Distrito Federal, Brazil; ^27^Secretaria de Vigilância em Saúde/Ministério da Saúde, Brasília, Distrito Federal, Brazil; ^28^Instituto de Higiene e Medicina Tropical, Universidade Nova de Lisboa, Lisboa, Portugal; ^29^Latin American Genomic Surveillance Arboviral Network; ^30^Marie Bashir Institute for Infectious Diseases and Biosecurity, School of Life and Environmental Sciences and School of Medical Sciences, University of Sydney, Sydney, Australia; ^31^KwaZulu-Natal Research Innovation and Sequencing Platform (KRISP), College of Health Sciences, University of KwaZulu-Natal, Durban, South Africa; ^32^Department of Zoology, Peter Medawar Building, University of Oxford, Oxford, United Kingdom.

^These authors contributed equally: Marta Giovanetti, Vagner Fonseca

These authors jointly supervised this work: José Lourenço, Luiz Carlos Junior Alcantara

***Correspondence to:** giovanetti.marta@gmail.com; jose.lourenco@zoo.ox.ac.uk; luiz.alcantara@ioc.fiocruz.br

**Supplementary Figure 1**


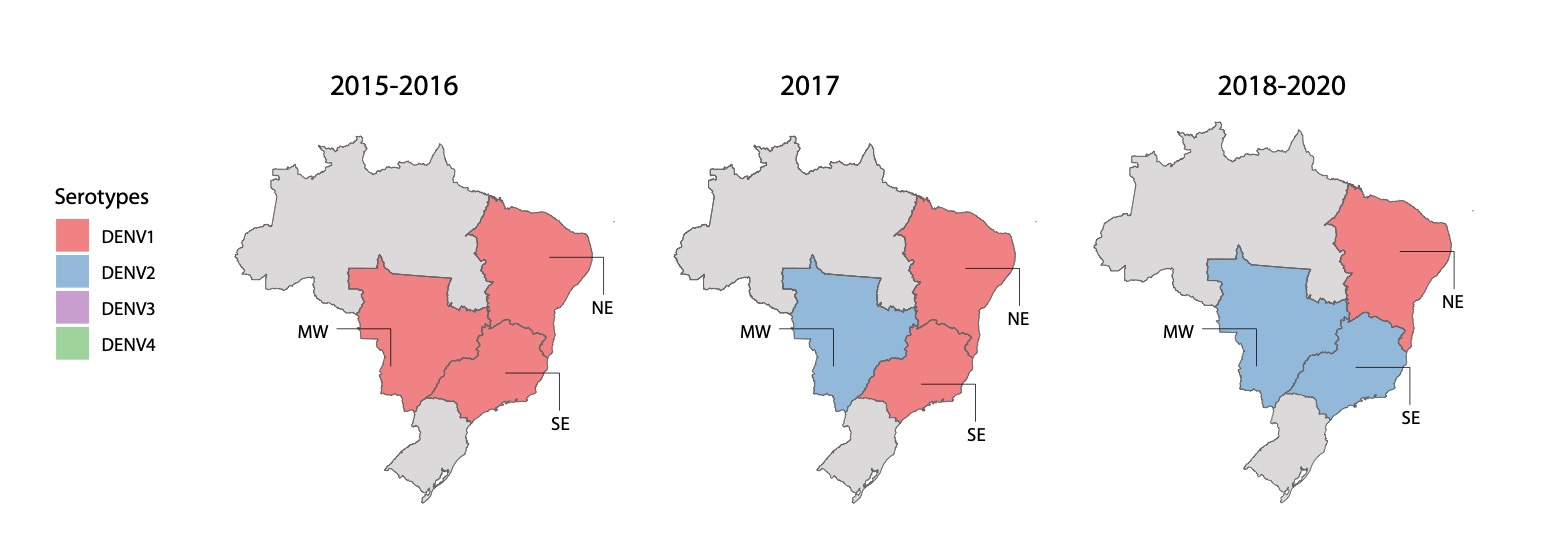


**Supplementary Figure 1. Patterns of serotype dominance 2015-2020.** Map of Brazil showing the progression of serotypes in the three geographic regions sampled in this study - Midwest (MW), Northeast (NE), and Southeast (SE) - between 2015 and 2020 (until EW06). Brazilian macro regions are coloured according to the dominant serotype. Grey represents northern and southern Brazilian macro regions. The initial map of Brazilian regions was obtained from the R package “get_brmap” (available at: <https://rdrr.io/cran/brazilmaps/man/get_brmap.html>). Source data are provided as a Source Data file.

**
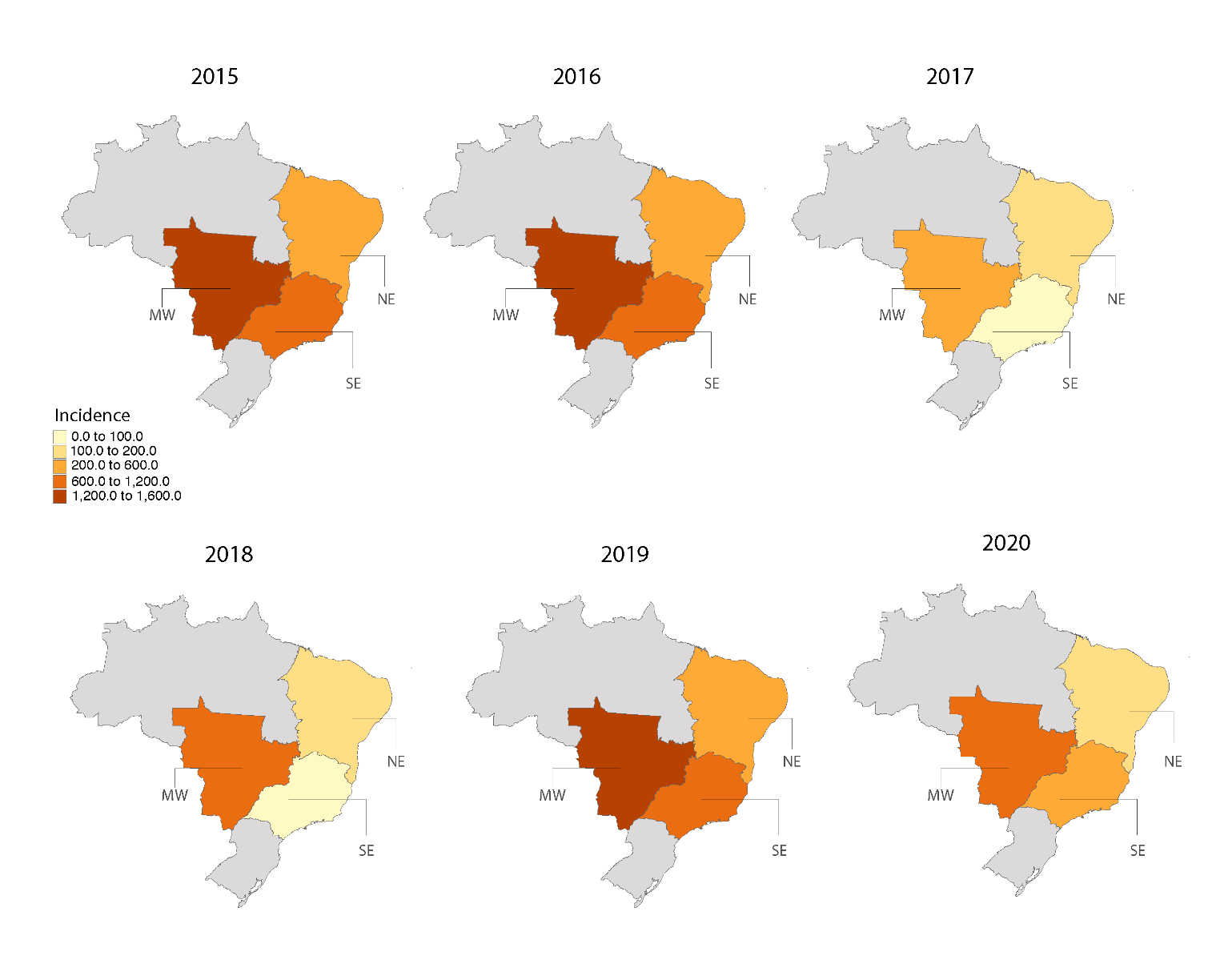
Supplementary Figure 2**

**Supplementary Figure 2. Maps of yearly incidence of dengue in Brazil, 2015-2020.** Maps of Brazil divided by regions (SE=Southeast, NE=Northeast, MW=Midwest) showing the yearly incidence of dengue between 2015 and 2020 (until EW06). Incidence = cases per 100,000 inhabitants per year. Grey represents northern and southern Brazilian macro regions. The initial map of Brazilian regions was obtained from the R package “get_brmap” (available at: <https://rdrr.io/cran/brazilmaps/man/get_brmap.html>).

**Supplementary Figure 3**

**Supplementary Figure 3.** **Mosquito-borne viral suitability measure (index P).** **a.** Daily mosquito-viral suitability measure (index P) for each of the macro regions from which new genomes were generated. The index P is obtained by averaging the climatic time series of the three largest urban centers in each region and then independently estimating index P. These series are presented in Figure 1 of the main text. **b.** Daily mosquito-viral suitability measure (index P) for the three cities per macro region used for panel A and for the main text. Cities are named in the color legend and tagged with their macro region. **(a-b)** The entomological and epidemiological priors used to estimate P are the (dengue) default for the MVSE R-package^49^ (see Supplementary File 1). Source data are provided as a Source Data file.

**Supplementary Figure 4**

**
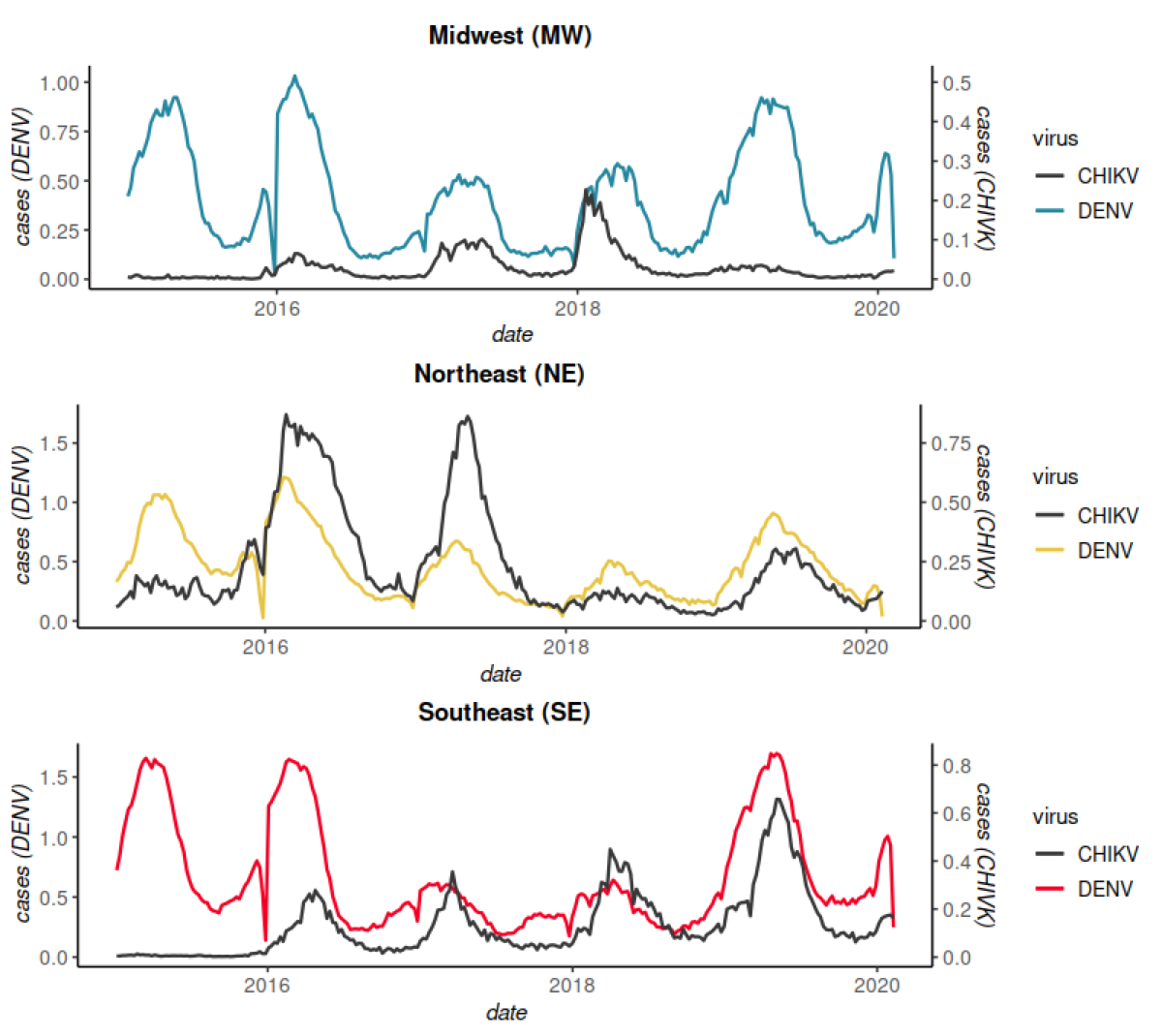
**

**Supplementary Figure 4. Weekly notified dengue cases normalized per 100K individuals per region in 2015-2020 (until EW06).** DENV epidemic curves are coloured according to geographical macro region: SE=Southeast, NE=Northeast, MW=Midwest. CHIKV epidemic curves are coloured in black for each of the macro regions. Incidence (cases per 100K population) is presented in log10 for visual purposes. Source data are provided as a Source Data file.

**Supplementary Figure 5**

**Supplementary Figure 5. DENV mortality in Brazil, 2015-2019. a.** Boxplot of the case fatality rate (deaths/cases) of dengue in the three Brazilian regions sampled in this study, 2015-2019. Boxplots are coloured according to Brazilian geographical region, Midwest (MW) n=8, Northeast (NE) n=11, and Southeast (SE) n=20. Boxplots show interquartile ranges, white lines are medians and box whiskers show the full range of posterior distribution. **b-c.** Time series of the incidence of deaths (number of fatal dengue /number of cases) reported weekly (c) and case fatality rate (number of fatal dengue /number of cases per 100K population) (d) in MW. **d-e.** Same as b and c but for NE. **f-g.** Same as b and c but for SE. Dates with no deaths reported were assumed to be reflective of no deaths, although it is possible that some instances of no deaths reflect a lack of reporting. Source data are provided as a Source Data file.**Supplementary Figure 6**


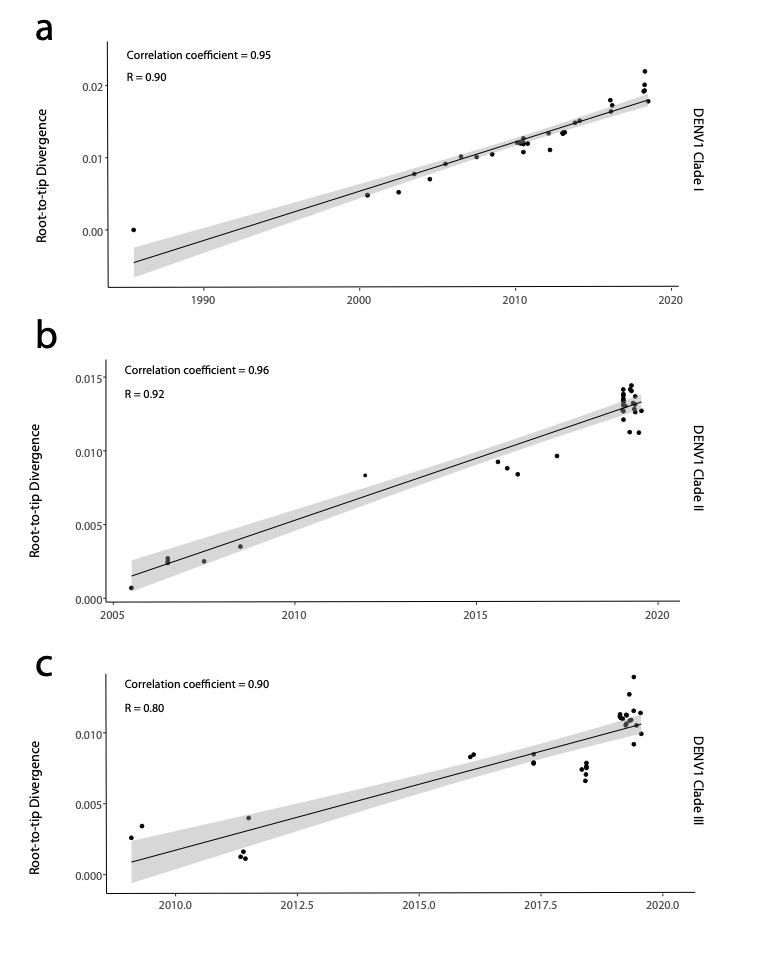


**Supplementary Figure 6. Analysis of temporal structure in DENV1 genotype V clades I-III**. **a.** Root-to-tip genetic divergence of Clade I against time of sampling (*n*=33) **b.** Root-to-tip genetic divergence for Clade II against time of sampling (*n*=34). **c.** Root-to-tip genetic divergence for Clade III against time of sampling (*n*=34). Black lines represent the medium values of the linear regression despite the grey ones represent the interval among the minimum and maximum values.

**Supplementary Figure 7**


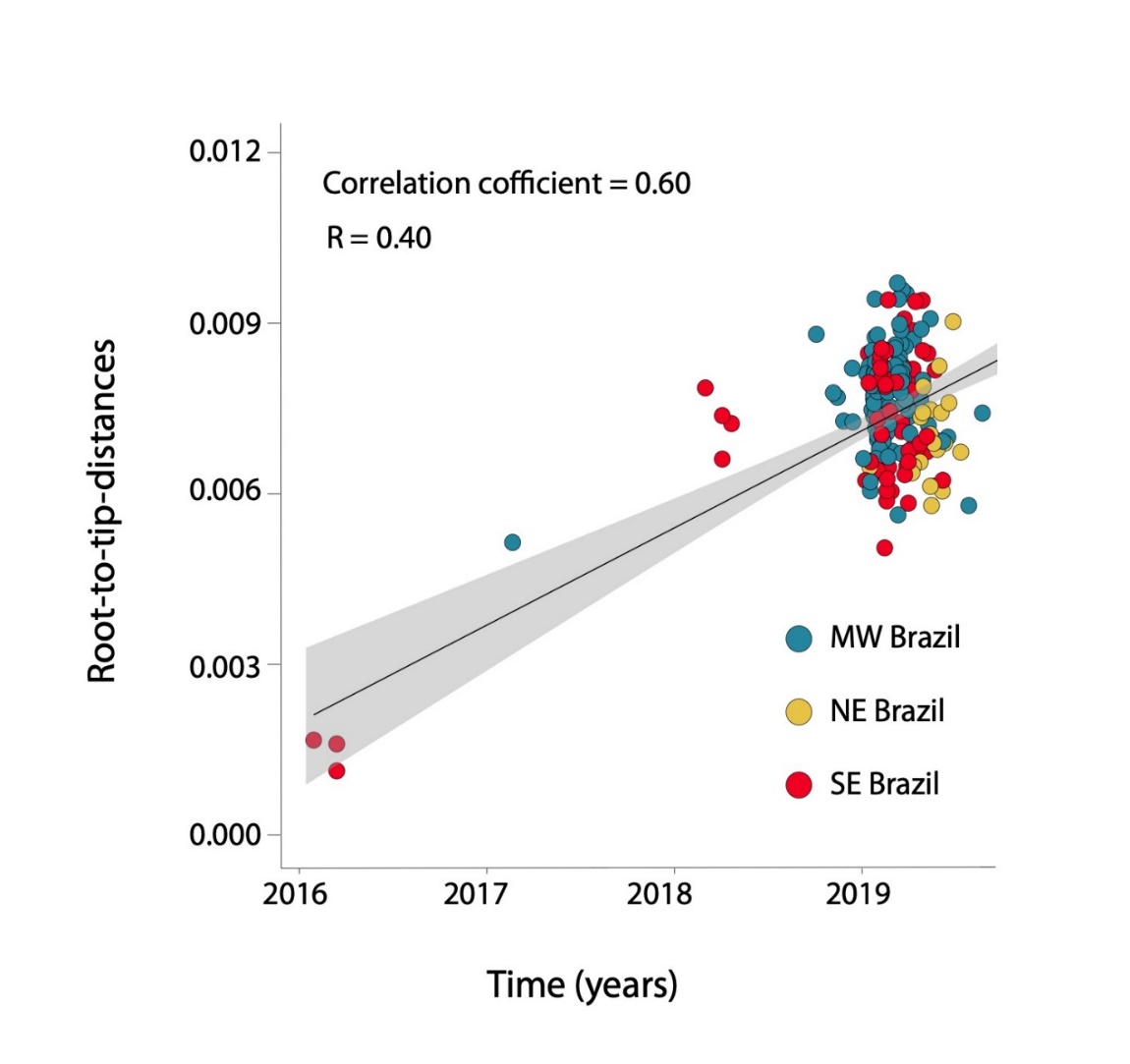


**Supplementary Figure 7. Genetic divergence regressed against date of sample collection for the BR-4 clade of DENV2 (*n*=181).** MW=Brazilian Midwest, NE Brazil=Brazilian Northeast, SE Brazil=Brazilian Southeast. Black lines represent the medium values of the linear regression despite the grey ones represent the interval among the minimum and maximum values.

**Supplementary Figure 8**

**Supplementary Figure 8. Number of complete DENV1 and DENV2 genomes sequences from Brazil, 1988-2020. a.** Comparative bar chart of the number of DENV1 genotype V complete genomes sequences generated in Brazil between 1988 and 2020 and available in public databases until May 2020. Gray bars represent the genomes from this study, while green bars indicate the genomes from previous studies. **b.** Comparative bar chart of the number of DENV2 genotype III complete genomes sequences generated in Brazil between 1990 and 2020 and available in public databases until May 2020. Red bars represent the genomes from this study, while green bars indicate the genomes from previous studies. Source data are provided as a Source Data file.

**Supplementary Figure 9**

**
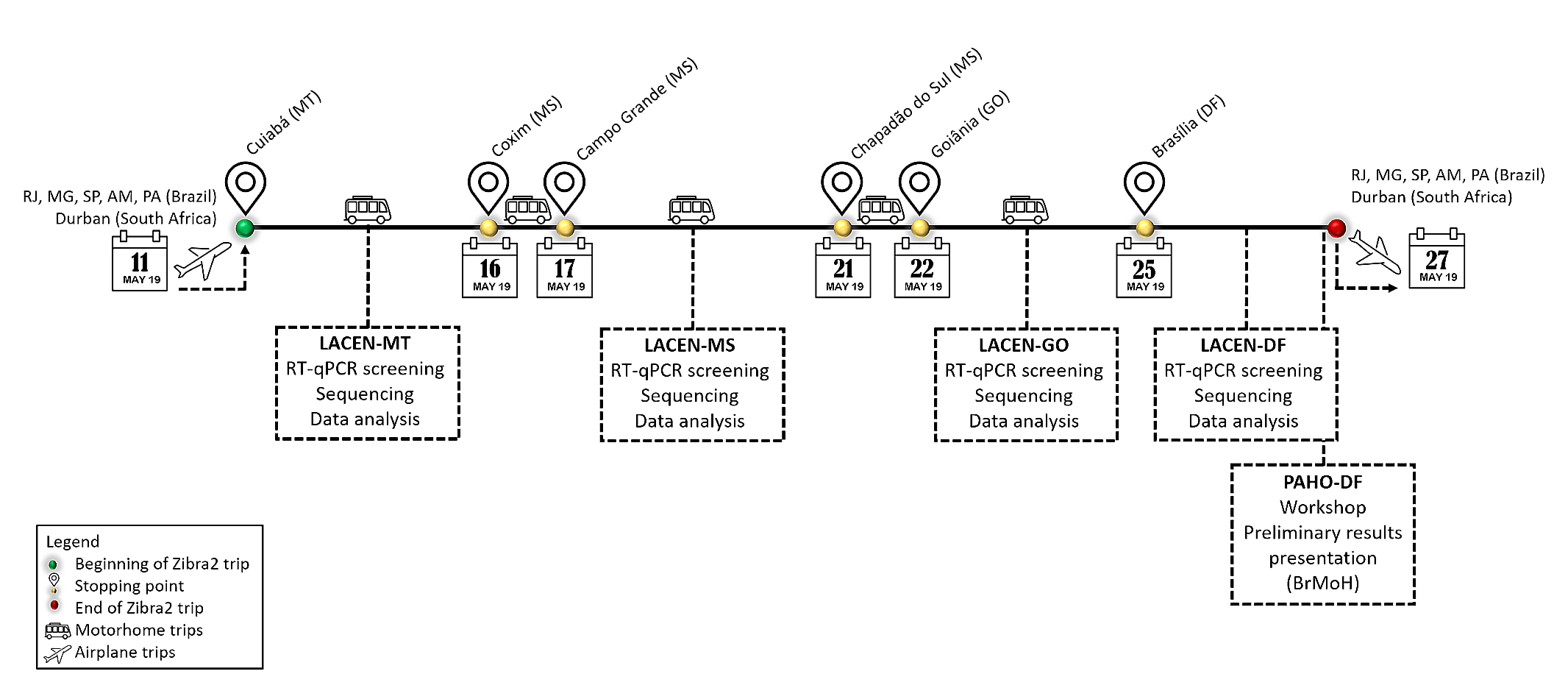
**

**Supplementary Figure 9.** **Timeline of the ZIBRA2 itinerant project across the Brazilian Midwest region.** Researchers from Brazil (RJ=Rio de Janeiro, MG=Minas Gerais, SP=São Paulo, AM=Amazonas, and PA=Pará) and from Durban (South Africa) visited the public health laboratories (LACEN) in the Brazilian Midwest region (showed above the line) on board a mobile laboratory (motorhome) to carry out the field genomic surveillance activities (showed below the line). The event ended in a workshop held at PAHO/WHO to present the preliminary results to the health authorities. MT=Mato Grosso, MS=Mato Grosso do Sul, GO=Goiás, DF=Brazilian Federal District. The Bus and Airplane icons were created by Mariagloria Posani and Kiddo, respectively, and are currently available at the Noun Project (<https://thenounproject.com/>).

**Supplementary Table 1. Number of molecularly confirmed DENV cases in the three Brazilian macro regions, 2015-2020.**

|  |  | **YEAR** | | | | | |
| --- | --- | --- | --- | --- | --- | --- | --- |
| **REGION** | **SEROTYPE** | **2015** | **2016** | **2017** | **2018** | **2019** | **2020^*^** |
| **MW** | DENV1 | 2467 | 970 | 55 | 16 | 27 | 40 |
|  | DENV2 | 28 | 19 | 231 | 520 | 2361 | 2184 |
|  | DENV3 | 2 | 2 | 1 | 0 | 0 | 20 |
|  | DENV4 | 219 | 64 | 23 | 1 | 2 | 0 |
| **NE** | DENV1 | 387 | 66 | 15 | 245 | 1398 | 68 |
|  | DENV2 | 15 | 7 | 3 | 38 | 456 | 36 |
|  | DENV3 | 37 | 17 | 0 | 0 | 0 | 0 |
|  | DENV4 | 44 | 0 | 0 | 1 | 0 | 0 |
| **SE** | DENV1 | 2145 | 1703 | 48 | 132 | 274 | 173 |
|  | DENV2 | 15 | 81 | 19 | 333 | 3068 | 1086 |
|  | DENV3 | 0 | 7 | 10 | 1 | 5 | 2 |
|  | DENV4 | 72 | 13 | 1 | 0 | 3 | 0 |

MW=Midwest region; NE=Northeast region; SE=Southeast region

^*^ Until EW06

**Supplementary Table 2. Information on the 227 sequenced samples of DENV1 and DENV2.**

| **ID** | **Serotype** | **Sample** | **Collection date** | **Date onset symptoms** | **Sex** | **Age** | **Municipality** | **State** | **Ct** | **DENV Classification** |
| --- | --- | --- | --- | --- | --- | --- | --- | --- | --- | --- |
| **OPAS 41** | DENV1 | Serum | 16/01/2019 | 16/01/2019 | Female | 77 | Feira de Santana | BA | 17 | Dengue with no warning signs |
| **OPAS 42** | DENV1 | Serum | 17/01/2019 | 17/01/2019 | Male | 25 | Feira de Santana | BA | 17 | Dengue with no warning signs |
| **OPAS 43** | DENV1 | Serum | 17/01/2019 | 17/01/2019 | Female | 59 | Feira de Santana | BA | 22 | Dengue with no warning signs |
| **OPAS 44** | DENV1 | Serum | 18/01/2019 | 18/01/2019 | Female | 30 | Feira de Santana | BA | 24 | Dengue with no warning signs |
| **OPAS 45** | DENV1 | Serum | 17/01/2019 | 17/01/2019 | Female | 4 | Feira de Santana | BA | 26 | Dengue with no warning signs |
| **OPAS 46** | DENV1 | Serum | 17/01/2019 | 17/01/2019 | Female | 8 | Feira de Santana | BA | 23 | Dengue with no warning signs |
| **OPAS 47** | DENV1 | Serum | 18/01/2019 | 17/01/2019 | Female | 13 | Feira de Santana | BA | 26 | Dengue with no warning signs |
| **OPAS 48** | DENV1 | Serum | 17/01/2019 | 17/01/2019 | Male | 20 | Feira de Santana | BA | 20 | Dengue with no warning signs |
| **OPAS 49** | DENV1 | Serum | 21/01/2019 | 21/01/2019 | Female | 14 | Feira de Santana | BA | 20 | Dengue with no warning signs |
| **OPAS 50** | DENV1 | Serum | 15/01/2019 | 15/01/2019 | Female | 21 | Feira de Santana | BA | 22 | Dengue with no warning signs |
| **OPAS 56** | DENV1 | Serum | 16/05/2019 | 16/05/2019 | Female | 11 | Coração de Maria | BA | 27 | Dengue with no warning signs |
| **OPAS 57** | DENV1 | Serum | 08/04/2019 | 07/04/2019 | Female | 19 | Barreiras | BA | 20 | Dengue with no warning signs |
| **OPAS 58** | DENV1 | Serum | 25/04/2019 | 20/04/2019 | Male | 15 | Feira de Santana | BA | 26 | Dengue with no warning signs |
| **OPAS 60** | DENV1 | Serum | 09/01/2019 | 07/01/2019 | Male | 31 | Feira de Santana | BA | 21 | Dengue with no warning signs |
| **OPAS 61** | DENV1 | Serum | 06/02/2019 | 06/02/2019 | Male | 35 | Coração de Maria | BA | 16 | Dengue with no warning signs |
| **OPAS 62** | DENV1 | Serum | 07/05/2019 | 04/05/2019 | Female | 28 | Coração de Maria | BA | 26 | Dengue with no warning signs |
| **OPAS 63** | DENV1 | Serum | 16/05/2019 | 12/05/2019 | Female | 27 | Coração de Maria | BA | 19 | Dengue with no warning signs |
| **OPAS 64** | DENV1 | Serum | 27/03/2019 | 21/03/2019 | Male | 50 | Coração de Maria | BA | 23 | Dengue with no warning signs |
| **OPAS 65** | DENV1 | Serum | 14/05/2019 | 10/05/2019 | Female | 13 | Coração de Maria | BA | 26 | Dengue with no warning signs |
| **OPAS 157** | DENV1 | Serum | 05/04/2019 | NA | Female | 34 | Brasília | DF | 16 | Severe dengue |
| **OPAS 158** | DENV1 | Serum | 18/07/2019 | NA | Female | 29 | Brasília | DF | 19 | Severe dengue |
| **GO03** | DENV1 | Serum | 06/11/2015 | 02/11/2015 | Female | 41 | Aparecida de Goiânia | GO | NA | Dengue with no warning signs |
| **GO04** | DENV1 | Serum | 05/08/2015 | 01/08/2015 | Female | 21 | Goiânia | GO | NA | Dengue with no warning signs |
| **OPAS 131** | DENV1 | Serum | 07/03/2019 | 06/03/2019 | Female | 25 | Valparaíso | GO | 17 | Dengue with no warning signs |
| **OPAS 132** | DENV1 | Serum | 03/04/2019 | 30/03/2019 | Female | 62 | Novo Gama | GO | 30 | Dengue with no warning signs |
| **OPAS 133** | DENV1 | Serum | 02/04/2019 | 29/03/2019 | Female | 65 | Valparaíso | GO | 24 | Dengue with no warning signs |
| **OPAS 134** | DENV1 | Serum | 09/04/2019 | 08/04/2019 | Female | 26 | Goiânia | GO | 20 | Dengue with no warning signs |
| **OPAS 135** | DENV1 | Serum | 13/02/2019 | 08/02/2019 | Female | 47 | Rio Verde | GO | 28 | Dengue with no warning signs |
| **OPAS 136** | DENV1 | Serum | 15/02/2019 | 11/02/2019 | Female | 49 | Caldas Novas | GO | 21 | Dengue with no warning signs |
| **OPAS 137** | DENV1 | Serum | 21/02/2019 | 16/02/2019 | Female | 14 | Rio Verde | GO | 22 | Dengue with no warning signs |
| **OPAS 138** | DENV1 | Serum | 29/03/2019 | 28/03/2019 | Male | 21 | Valparaíso | GO | 27 | Dengue with no warning signs |
| **OPAS 90** | DENV1 | Serum | 13/02/2016 | 11/02/2016 | Female | 49 | Conselheiro Lafaiete | MG | 16 | Death |
| **OPAS 92** | DENV1 | Serum | 14/03/2016 | 09/03/2016 | Male | 67 | Juiz de Fora | MG | 19 | Death |
| **OPAS 99** | DENV1 | Serum | 19/04/2018 | 16/04/2018 | Female | 33 | Montes Claros | MG | 19 | Dengue with no warning signs |
| **OPAS 100** | DENV1 | Serum | 12/04/2018 | 10/04/2018 | Female | 30 | Janaúba | MG | 20 | Dengue with no warning signs |
| **OPAS 101** | DENV1 | Serum | 12/04/2018 | 09/04/2018 | Female | 43 | Janaúba | MG | 23 | Dengue with no warning signs |
| **OPAS 102** | DENV1 | Serum | 22/03/2018 | 19/03/2018 | Female | 35 | Belo Horizonte | MG | 15 | Dengue with no warning signs |
| **OPAS 103** | DENV1 | Serum | 05/07/2018 | 02/07/2018 | Female | 32 | Coração de Jesus | MG | 18 | Dengue with no warning signs |
| **OPAS 165** | DENV1 | Serum | 21/03/2017 | 18/03/2017 | Male | 35 | Brasília de Minas | MG | 20 | Dengue with no warning signs |
| **OPAS 167** | DENV1 | Serum | 09/05/2017 | 07/05/2017 | Male | 19 | São Francisco | MG | 18 | Dengue with no warning signs |
| **OPAS 168** | DENV1 | Serum | 08/05/2017 | 05/05/2017 | Male | 53 | Varzelandia | MG | 18 | Dengue with no warning signs |
| **OPAS 169** | DENV1 | Serum | 08/05/2017 | 05/05/2017 | Female | 72 | Varzelandia | MG | 19 | Dengue with no warning signs |
| **OPAS 148** | DENV1 | Liver | 28/04/2019 | 24/04/2019 | Female | 12 | Recife | PE | 25 | Death |
| **OPAS 149** | DENV1 | Liver | 29/05/2019 | 12/05/2019 | Female | 30 | Cabo de Santo Agostino | PE | 29 | Death |
| **OPAS 150** | DENV1 | Liver | 28/05/2019 | 21/05/2019 | Female | 36 | Buíque | PE | 25 | Death |
| **OPAS 151** | DENV1 | Spleen | 18/06/2019 | 10/06/2019 | Male | 15 | Ipojuca | PE | 29 | Death |
| **OPAS 152** | DENV1 | Liver | 22/06/2019 | 19/06/2019 | Male | 78 | Sertânia | PE | 26 | Death |
| **OPAS 153** | DENV1 | Spleen | 18/07/2019 | 14/07/2019 | Male | 5 | Recife | PE | 24 | Death |
| **OPAS 154** | DENV1 | Liver | 24/07/2019 | 18/07/2019 | Male | 14 | Carpina | PE | 24 | Death |
| **OPAS 155** | DENV1 | Serum | 09/05/2019 | 09/05/2019 | Male | 14 | Custódia | PE | 21 | Dengue with no warning signs |
| **OPAS 156** | DENV1 | Serum | 29/05/2019 | 26/05/2019 | Female | 18 | Jaboatão dos Guararapes | PE | 17 | Dengue with no warning signs |
| **OPAS 162** | DENV1 | Spleen | 22/03/2019 | 16/03/2019 | Male | 29 | Bom Conselho | PE | 24 | Death |
| **OPAS 163** | DENV1 | Spleen | 25/04/2019 | 20/04/2019 | Male | 6 | Timbaúba | PE | 24 | Death |
| **OPAS 171** | DENV1 | Serum | 19/01/2016 | 25/01/2016 | Male | 29 | Ribeirão Preto | SP | 35 | Dengue with no warning signs |
| **OPAS 173** | DENV1 | Serum | 29/01/2016 | 01/02/2016 | Female | 42 | Ribeirão Preto | SP | 22 | Dengue with no warning signs |
| **OPAS 174** | DENV1 | Serum | 13/02/2016 | 17/02/2016 | Female | 23 | Ribeirão Preto | SP | 33 | Dengue with no warning signs |
| **OPAS 175** | DENV1 | Serum | 20/02/2016 | 23/02/2016 | Female | 28 | Ribeirão Preto | SP | 19 | Dengue with no warning signs |
| **OPAS 51** | DENV2 | Serum | 25/04/2019 | 21/04/2019 | Male | 54 | Barreiras | BA | 25 | Death |
| **OPAS 52** | DENV2 | Serum | 29/04/2019 | 28/04/2019 | Female | 16 | Barreiras | BA | 28 | Dengue with no warning signs |
| **OPAS 53** | DENV2 | Serum | 07/06/2019 | 05/06/2019 | Female | 36 | Feira de Santana | BA | 24 | Dengue with no warning signs |
| **OPAS 54** | DENV2 | Serum | 16/01/2019 | 12/01/2019 | Female | 11 | Irece | BA | 24 | Dengue with no warning signs |
| **OPAS 55** | DENV2 | Serum | 11/04/2019 | 08/04/2019 | Female | 38 | Barreiras | BA | 26 | Dengue with no warning signs |
| **OPAS 110** | DENV2 | Serum | 14/05/2019 | 13/05/2019 | Male | 20 | Barreiras | BA | 25 | Dengue with no warning signs |
| **OPAS 111** | DENV2 | Serum | 19/06/2019 | 16/06/2019 | Male | 35 | Juazeiro | BA | 21 | Severe dengue |
| **OPAS 112** | DENV2 | Serum | 11/06/2019 | 10/06/2019 | Female | 16 | Presidente Dutra | BA | 29 | Death |
| **OPAS 113** | DENV2 | Serum | 06/06/2019 | 03/06/2019 | Female | 6 | Feira de Santana | BA | 26 | Severe dengue |
| **OPAS 114** | DENV2 | Serum | 12/07/2019 | 12/07/2019 | Female | 61 | Salvador | BA | 27 | Dengue with no warning signs |
| **OPAS 115** | DENV2 | Serum | 04/06/2019 | 01/06/2019 | Female | 43 | Salvador | BA | 27 | Dengue with no warning signs |
| **OPAS 116** | DENV2 | Serum | 30/04/2019 | 26/04/2019 | Female | 15 | Barreiras | BA | 28 | Dengue with no warning signs |
| **OPAS 117** | DENV2 | Serum | 24/04/2019 | 20/04/2019 | Male | 34 | Barreiras | BA | 25 | Dengue with no warning signs |
| **OPAS 118** | DENV2 | Serum | 08/04/2019 | 06/04/2019 | Female | 39 | Barreiras | BA | 25 | Dengue with no warning signs |
| **OPAS 119** | DENV2 | Serum | 16/05/2019 | 14/05/2019 | Female | 20 | Barreiras | BA | 26 | Dengue with no warning signs |
| **OPAS 120** | DENV2 | Serum | 27/05/2019 | 24/05/2019 | Female | 63 | Barreiras | BA | 22 | Dengue with no warning signs |
| **OPAS 121** | DENV2 | Serum | 14/05/2019 | 12/05/2019 | Male | 24 | Barreiras | BA | 29 | Dengue with no warning signs |
| **OPAS 122** | DENV2 | Serum | 21/05/2019 | 19/05/2019 | Female | 12 | Barreiras | BA | 29 | Dengue with no warning signs |
| **OPAS 123** | DENV2 | Serum | 20/05/2019 | 18/05/2019 | Female | 21 | Barreiras | BA | 22 | Dengue with no warning signs |
| **OPAS 124** | DENV2 | Serum | 14/05/2019 | 10/05/2019 | Female | 24 | Barreiras | BA | 28 | Dengue with no warning signs |
| **OPAS 161** | DENV2 | Serum | 23/08/2019 | NA | Male | 44 | Brasília | DF | 21 | Severe dengue |
| **GO72** | DENV2 | Liver | 25/04/2019 | 12/04/2019 | Male | 53 | Goiania | GO | 25 | Death |
| **GO75** | DENV2 | Serum | 29/04/2019 | 28/04/2019 | Female | 28 | Goiania | GO | 20 | Dengue with no warning signs |
| **OPAS 66** | DENV2 | Serum | 17/01/2019 | 16/01/2019 | Female | 40 | Luziânia | GO | 19 | Dengue with no warning signs |
| **OPAS 67** | DENV2 | Serum | 17/01/2019 | 15/01/2019 | Male | 44 | Caldas Novas | GO | 16 | Dengue with no warning signs |
| **OPAS 68** | DENV2 | Serum | 18/01/2019 | 14/01/2019 | Male | 61 | Firminópolis | GO | 22 | Dengue with no warning signs |
| **OPAS 69** | DENV2 | Serum | 24/01/2019 | 22/01/2019 | Male | 29 | Caldas Novas | GO | 20 | Dengue with no warning signs |
| **OPAS 70** | DENV2 | Serum | 28/01/2019 | 25/01/2019 | Female | 39 | Caldas Novas | GO | 19 | Dengue with no warning signs |
| **OPAS 71** | DENV2 | Serum | 01/02/2019 | 29/01/2019 | Male | 24 | Goiânia | GO | 19 | Dengue with no warning signs |
| **OPAS 72** | DENV2 | Serum | 31/01/2019 | 28/01/2019 | Female | 79 | São Luís de Montes Belos | GO | 20 | Dengue with no warning signs |
| **OPAS 74** | DENV2 | Serum | 04/02/2019 | 02/02/2019 | Female | 23 | Caldas Novas | GO | 19 | Dengue with no warning signs |
| **OPAS 75** | DENV2 | Serum | 07/02/2019 | 06/02/2019 | Female | 20 | Goiânia | GO | 19 | Dengue with no warning signs |
| **OPAS 76** | DENV2 | Serum | 11/02/2019 | 08/02/2019 | Male | 64 | Firminópolis | GO | 18 | Dengue with no warning signs |
| **OPAS 77** | DENV2 | Serum | 11/02/2019 | 09/02/2019 | Male | 35 | Turvânia | GO | 16 | Dengue with no warning signs |
| **OPAS 78** | DENV2 | Serum | 11/02/2019 | 08/02/2019 | Male | 60 | Rio Verde | GO | 17 | Dengue with no warning signs |
| **OPAS 79** | DENV2 | Serum | 21/02/2019 | 18/02/2019 | Male | 84 | Goiânia | GO | 18 | Dengue with no warning signs |
| **OPAS 80** | DENV2 | Serum | 22/02/2019 | 20/02/2019 | Female | 18 | Corumbá | GO | 16 | Dengue with no warning signs |
| **OPAS 81** | DENV2 | Serum | 18/02/2019 | 16/02/2019 | Female | 41 | Rio Verde | GO | 18 | Dengue with no warning signs |
| **OPAS 82** | DENV2 | Serum | 01/03/2019 | 27/02/2019 | Female | 29 | Goiânia | GO | 17 | Dengue with no warning signs |
| **OPAS 83** | DENV2 | Serum | 12/03/2019 | 11/03/2019 | Male | 42 | Caldas Novas | GO | 19 | Dengue with no warning signs |
| **OPAS 84** | DENV2 | Serum | 18/03/2019 | 16/03/2019 | Female | 36 | Paraúna | GO | 18 | Dengue with no warning signs |
| **OPAS 85** | DENV2 | Serum | 26/03/2019 | 22/03/2019 | Female | 79 | Goiatuba | GO | 18 | Dengue with no warning signs |
| **OPAS 86** | DENV2 | Serum | 04/04/2019 | 31/03/2019 | Male | 22 | Goiânia | GO | 24 | Dengue with no warning signs |
| **OPAS 87** | DENV2 | Serum | 27/03/2019 | 26/03/2019 | Male | 30 | Israelândia | GO | 21 | Dengue with no warning signs |
| **OPAS 89** | DENV2 | Serum | 27/03/2019 | 24/03/2019 | Male | 40 | Israelândia | GO | 21 | Dengue with no warning signs |
| **OPAS 125** | DENV2 | Liver | 20/02/2017 | 16/02/2017 | Male | 47 | Goiânia | GO | 23 | Death |
| **OPAS 126** | DENV2 | Spleen | 06/11/2018 | 01/11/2018 | Female | 47 | Rio Verde | GO | 22 | Death |
| **OPAS 127** | DENV2 | Liver | 06/02/2019 | 03/02/2019 | Female | 63 | Turvânia | GO | 25 | Death |
| **OPAS 128** | DENV2 | Liver | 23/02/2019 | 19/02/2019 | Male | 36 | Anápolis | GO | 22 | Death |
| **OPAS 129** | DENV2 | Serum | 10/05/2019 | 04/05/2019 | Female | 76 | Goiânia | GO | 20 | Death |
| **OPAS 130** | DENV2 | Spleen | 17/06/2019 | 13/06/2019 | Male | 25 | Goianira | GO | 19 | Death |
| **OPAS 139** | DENV2 | Serum | 28/02/2008 | 25/02/2008 | Female | 34 | Goiânia | GO | 20 | Dengue with no warning signs |
| **OPAS 140** | DENV2 | Serum | 04/03/2008 | 02/03/2008 | Female | 38 | Aparecida de Goiânia | GO | 26 | Dengue with no warning signs |
| **OPAS 142** | DENV2 | Serum | 11/03/2008 | 09/03/2008 | Male | 23 | Aparecida de Goiânia | GO | 16 | Dengue with no warning signs |
| **OPAS 159** | DENV2 | Serum | 14/05/2019 | NA | Male | 43 | Valparaíso | GO | 25 | Severe dengue |
| **OPAS 01** | DENV2 | Serum | 07/02/2019 | 07/02/2019 | Male | 77 | Betim | MG | 28 | Death |
| **OPAS 02** | DENV2 | Serum | 18/03/2019 | 15/03/2019 | Male | 80 | Uberaba | MG | 35 | Death |
| **OPAS 03** | DENV2 | Serum | 27/03/2019 | 25/03/2019 | Male | 90 | Uberlandia | MG | 25 | Death |
| **OPAS 04** | DENV2 | Serum | 14/01/2019 | 12/01/2019 | Female | 53 | Uberaba | MG | 20 | Dengue with no warning signs |
| **OPAS 05** | DENV2 | Serum | 10/04/2019 | 07/04/2019 | Male | 49 | Jaboticatubas | MG | 23 | Death |
| **OPAS 06** | DENV2 | Serum | 31/01/2019 | 30/01/2019 | Male | 46 | Unai | MG | 23 | Dengue with no warning signs |
| **OPAS 07** | DENV2 | Serum | 31/01/2019 | 29/01/2019 | Male | 59 | Unai | MG | 23 | Dengue with no warning signs |
| **OPAS 08** | DENV2 | Serum | 09/05/2019 | 08/05/2019 | Female | 54 | Ribeirao das Neves | MG | 22 | Death |
| **OPAS 09** | DENV2 | Serum | 19/02/2019 | 18/02/2019 | Male | 39 | Uberaba | MG | 21 | Dengue with no warning signs |
| **OPAS 10** | DENV2 | Serum | 01/02/2019 | 29/01/2019 | Male | 55 | Patrocinio | MG | 26 | Death |
| **OPAS 11** | DENV2 | Serum | 25/05/2019 | 21/05/2019 | Male | 35 | Ribeirao das Neves | MG | 25 | Death |
| **OPAS 12** | DENV2 | Serum | 23/05/2019 | 23/05/2019 | Male | 6 | Patos de Minas | MG | 27 | Death |
| **OPAS 13** | DENV2 | Serum | 12/02/2019 | 09/02/2019 | Female | 39 | Arinos | MG | 21 | Dengue with no warning signs |
| **OPAS 14** | DENV2 | Serum | 18/02/2019 | 16/02/2019 | Male | 61 | Prata | MG | 20 | Dengue with no warning signs |
| **OPAS 15** | DENV2 | Serum | 18/02/2019 | 17/02/2019 | Female | 24 | Prata | MG | 20 | Dengue with no warning signs |
| **OPAS 16** | DENV2 | Serum | 05/02/2019 | 03/02/2019 | Female | 54 | Capinopolis | MG | 20 | Dengue with no warning signs |
| **OPAS 17** | DENV2 | Serum | 14/02/2019 | 12/02/2019 | Male | 79 | Ipiacu | MG | 21 | Dengue with no warning signs |
| **OPAS 18** | DENV2 | Serum | 24/02/2019 | 22/02/2019 | Male | 19 | Curvelo | MG | 35 | Death |
| **OPAS 19** | DENV2 | Serum | 26/02/2019 | 23/02/2019 | Male | 50 | Uberaba | MG | 16 | Dengue with no warning signs |
| **OPAS 20** | DENV2 | Serum | 27/02/2019 | 24/02/2019 | Female | 52 | Uberaba | MG | 19 | Dengue with no warning signs |
| **OPAS 21** | DENV2 | Serum | 22/02/2019 | 20/02/2019 | Female | 44 | Januaria | MG | 21 | Dengue with no warning signs |
| **OPAS 22** | DENV2 | Serum | 19/03/2019 | 18/03/2019 | Female | 16 | Uberlandia | MG | 20 | Dengue with no warning signs |
| **OPAS 23** | DENV2 | Serum | 19/03/2019 | 18/03/2019 | Female | 20 | Uberlandia | MG | 19 | Dengue with no warning signs |
| **OPAS 24** | DENV2 | Spleen | 28/03/2019 | 28/03/2019 | Female | 47 | Ibia | MG | 34 | Death |
| **OPAS 25** | DENV2 | Serum | 25/03/2019 | 23/03/2019 | Male | 43 | Uberaba | MG | 21 | Dengue with no warning signs |
| **OPAS 26** | DENV2 | Serum | 21/03/2019 | 20/03/2019 | Female | 20 | Uberaba | MG | 20 | Dengue with no warning signs |
| **OPAS 27** | DENV2 | Serum | 01/04/2019 | 31/03/2019 | Female | 6 | Belo Horizonte | MG | 32 | Death |
| **OPAS 28** | DENV2 | Serum | 29/03/2019 | 26/03/2019 | Female | 60 | Patrocinio | MG | 19 | Dengue with no warning signs |
| **OPAS 29** | DENV2 | Serum | 02/04/2019 | 30/03/2019 | Male | 46 | Buritis | MG | 18 | Dengue with no warning signs |
| **OPAS 30** | DENV2 | Serum | 01/04/2019 | 31/03/2019 | Female | 38 | Natalandia | MG | 21 | Dengue with no warning signs |
| **OPAS 31** | DENV2 | Serum | 08/03/2019 | 03/04/2019 | Male | 67 | Passos | MG | 34 | Death |
| **OPAS 32** | DENV2 | Serum | 12/04/2019 | 11/14/2019 | Male | 60 | Ituiutaba | MG | 17 | Dengue with no warning signs |
| **OPAS 33** | DENV2 | Serum | 12/04/2019 | 09/04/2019 | Female | 68 | Araguari | MG | 20 | Dengue with no warning signs |
| **OPAS 34** | DENV2 | Serum | 12/04/2019 | 10/04/2019 | Male | 77 | Ituiutaba | MG | 17 | Death |
| **OPAS 35** | DENV2 | Serum | 24/04/2019 | 20/04/2019 | Female | 65 | Campos Gerais | MG | 35 | Death |
| **OPAS 36** | DENV2 | Plasma | 07/05/2019 | 04/05/2019 | Female | 55 | Contagem | MG | 34 | Death |
| **OPAS 37** | DENV2 | Serum | 07/05/2019 | 05/05/2019 | Male | 26 | Ituiutaba | MG | 22 | Dengue with no warning signs |
| **OPAS 38** | DENV2 | Serum | 07/06/2019 | 04/06/2019 | Male | 26 | Montes Claros | MG | 25 | Death |
| **OPAS 39** | DENV2 | Serum | 06/04/2019 | 03/04/2019 | Female | 59 | Belo Horizonte | MG | 25 | Death |
| **OPAS 40** | DENV2 | Serum | 17/04/2019 | 12/04/2019 | Male | 11 | Belo Horizonte | MG | 35 | Death |
| **OPAS 93** | DENV2 | Serum | 29/01/2016 | 28/01/2016 | Female | 31 | Uberaba | MG | 25 | Dengue with no warning signs |
| **OPAS 95** | DENV2 | Serum | 14/03/2016 | 12/03/2016 | Female | 35 | Uberaba | MG | 23 | Dengue with no warning signs |
| **OPAS 96** | DENV2 | Serum | 14/03/2016 | 12/03/2016 | Male | 59 | Uberaba | MG | 26 | Dengue with no warning signs |
| **OPAS 105** | DENV2 | Serum | 22/04/2018 | 16/04/2018 | Female | 54 | Ituiutaba | MG | 21 | Dengue with no warning signs |
| **OPAS 106** | DENV2 | Serum | 04/04/2018 | 02/04/2018 | Female | 24 | Capinópolis | MG | 25 | Dengue with no warning signs |
| **OPAS 107** | DENV2 | Serum | 04/04/2018 | 03/04/2018 | Female | 39 | Capinópolis | MG | 19 | Dengue with no warning signs |
| **OPAS 108** | DENV2 | Serum | 02/03/2018 | 27/02/2018 | Female | 31 | Ituiutaba | MG | 25 | Dengue with no warning signs |
| **CG02** | DENV2 | Serum | 21/01/2019 | 20/01/2019 | Male | 40 | Campo Grande | MS | 24 | Dengue with no warning signs |
| **CG06** | DENV2 | Serum | 22/03/2019 | 20/03/2019 | Female | 46 | Campo Grande | MS | 21 | Dengue with no warning signs |
| **CG07** | DENV2 | Serum | 22/03/2019 | 21/03/2019 | Male | 12 | Campo Grande | MS | 20 | Dengue with no warning signs |
| **CG08** | DENV2 | Serum | 26/02/2019 | 23/02/2019 | Female | 31 | Jaraguari | MS | 31 | Dengue with no warning signs |
| **CG09** | DENV2 | Serum | 20/02/2019 | 18/02/2019 | Female | 4 | Camapuã | MS | 26 | Dengue with no warning signs |
| **CG10** | DENV2 | Serum | 07/03/2019 | 03/03/2019 | Female | 24 | Jaraguari | MS | 27 | Dengue with no warning signs |
| **CG11** | DENV2 | Serum | 14/12/2018 | 12/12/2018 | Male | 38 | Campo Grande | MS | 24 | Dengue with no warning signs |
| **CG12** | DENV2 | Serum | 04/10/2018 | 01/10/2018 | Male | 39 | Campo Grande | MS | 26 | Dengue with no warning signs |
| **CG13** | DENV2 | Serum | 14/11/2018 | 13/11/2018 | Female | 26 | Campo Grande | MS | 22 | Dengue with no warning signs |
| **CG14** | DENV2 | Serum | 26/11/2018 | 23/11/2018 | Female | 27 | Campo Grande | MS | 21 | Dengue with no warning signs |
| **CG15** | DENV2 | Serum | 13/12/2018 | 11/12/2018 | Female | 31 | Campo Grande | MS | 25 | Dengue with no warning signs |
| **CG16** | DENV2 | Serum | 26/01/2019 | 25/01/2019 | Male | 41 | Campo Grande | MS | 18 | Dengue with no warning signs |
| **CG17** | DENV2 | Serum | 24/01/2019 | 22/01/2019 | Male | 23 | Campo Grande | MS | 19 | Dengue with no warning signs |
| **CG18** | DENV2 | Serum | 22/03/2019 | 21/03/2019 | Female | 20 | Corumbá | MS | 26 | Dengue with no warning signs |
| **CG19** | DENV2 | Serum | 07/03/2019 | 06/03/2019 | Female | 13 | Dois Irmãos do Buriti | MS | 26 | Dengue with no warning signs |
| **CG20** | DENV2 | Serum | 13/03/2019 | 11/03/2019 | Female | 9 | Douradina | MS | 32 | Dengue with no warning signs |
| **CG21** | DENV2 | Serum | 14/03/2019 | 14/03/2019 | Female | 63 | Nioaque | MS | 22 | Dengue with no warning signs |
| **CG22** | DENV2 | Serum | 17/03/2019 | 15/03/2019 | Male | 24 | Mundo Novo | MS | 30 | Dengue with no warning signs |
| **CG23** | DENV2 | Serum | 31/01/2019 | 29/01/2019 | Male | 56 | Campo Grande | MS | 17 | Dengue with no warning signs |
| **CG24** | DENV2 | Serum | 19/03/2019 | 16/03/2019 | Male | 69 | Aparecida do Taboado | MS | 25 | Dengue with no warning signs |
| **CG68** | DENV2 | Serum | 25/01/2019 | 22/01/2019 | Female | 19 | Campo Grande | MS | 26 | Dengue with no warning signs |
| **CG69** | DENV2 | Serum | 23/01/2019 | 21/01/2019 | Female | 46 | Campo Grande | MS | 24 | Dengue with no warning signs |
| **CG70** | DENV2 | Serum | 26/01/2019 | 24/01/2019 | Male | 49 | Campo Grande | MS | 27 | Dengue with no warning signs |
| **CG71** | DENV2 | Serum | 02/02/2019 | 31/01/2019 | Male | 42 | Campo Grande | MS | 17 | Dengue with no warning signs |
| **CG81** | DENV2 | Serum | 31/01/2019 | 24/01/2019 | Female | 21 | Campo Grande | MS | 22 | Dengue with no warning signs |
| **CG86** | DENV2 | Serum | 31/01/2019 | 30/01/2019 | Male | 11 | Campo Grande | MS | 17 | Dengue with no warning signs |
| **CG87** | DENV2 | Serum | 25/01/2019 | 23/01/2019 | Male | 14 | Campo Grande | MS | 22 | Dengue with no warning signs |
| **CG90** | DENV2 | Serum | 23/01/2019 | 23/01/2019 | Male | 15 | Campo Grande | MS | 16 | Dengue with no warning signs |
| **CG94** | DENV2 | Serum | 21/01/2019 | 17/01/2019 | Female | 54 | Campo Grande | MS | 29 | Dengue with no warning signs |
| **CG96** | DENV2 | Serum | 20/01/2019 | 18/01/2019 | Female | 41 | Campo Grande | MS | 16 | Dengue with no warning signs |
| **CG99** | DENV2 | Serum | 06/03/2019 | 05/03/2019 | Male | 37 | Campo Grande | MS | 29 | Dengue with no warning signs |
| **CG100** | DENV2 | Serum | 17/03/2019 | 16/03/2019 | Female | 19 | Chapadão Do Sul | MS | 20 | Dengue with no warning signs |
| **CG102** | DENV2 | Serum | 27/01/2019 | 26/01/2019 | Male | 28 | Campo Grande | MS | 20 | Dengue with no warning signs |
| **CG103** | DENV2 | Serum | 26/01/2019 | 25/01/2019 | Male | 32 | Campo Grande | MS | 22 | Dengue with no warning signs |
| **CG104** | DENV2 | Serum | 23/01/2019 | 21/01/2019 | Female | 53 | Campo Grande | MS | 19 | Dengue with no warning signs |
| **CG105** | DENV2 | Serum | 27/01/2019 | 24/01/2019 | Female | 31 | Campo Grande | MS | 19 | Dengue with no warning signs |
| **CG106** | DENV2 | Serum | 24/01/2019 | 21/01/2019 | Female | 16 | Campo Grande | MS | 17 | Dengue with no warning signs |
| **CG107** | DENV2 | Serum | 31/01/2019 | 29/01/2019 | Male | 18 | Campo Grande | MS | 22 | Dengue with no warning signs |
| **CG108** | DENV2 | Serum | 26/03/2019 | 24/03/2019 | Male | 58 | Maracajú | MS | 20 | Dengue with no warning signs |
| **CG109** | DENV2 | Serum | 26/01/2019 | 23/01/2019 | Female | 52 | Campo Grande | MS | 18 | Dengue with no warning signs |
| **CG110** | DENV2 | Serum | 04/03/2019 | 01/03/2019 | Female | 25 | Campo Grande | MS | 26 | Dengue with no warning signs |
| **CG111** | DENV2 | Serum | 26/03/2019 | 25/03/2019 | Male | 13 | Corumbá | MS | 25 | Dengue with no warning signs |
| **CG112** | DENV2 | Limph node | 11/04/2019 | 08/04/2019 | Female | 7 | Campo Grande | MS | 24 | Death |
| **CG116** | DENV2 | Serum | 15/01/2019 | 13/01/2019 | Male | 13 | Campo Grande | MS | 26 | Dengue with no warning signs |
| **CG117** | DENV2 | Serum | 31/01/2019 | 30/01/2019 | Male | 70 | Campo Grande | MS | 21 | Dengue with no warning signs |
| **CG119** | DENV2 | Serum | 19/01/2019 | 17/01/2019 | Male | 37 | Campo Grande | MS | 16 | Dengue with no warning signs |
| **CG120** | DENV2 | Serum | 26/01/2019 | 22/01/2019 | Male | 13 | Campo Grande | MS | 30 | Dengue with no warning signs |
| **CG121** | DENV2 | Serum | 18/03/2019 | 15/03/2019 | Female | 7 | Pedro Gomes | MS | 24 | Dengue with no warning signs |
| **CG122** | DENV2 | Serum | 15/03/2019 | 13/03/2019 | Male | 35 | Nioaque | MS | 34 | Dengue with no warning signs |
| **CG123** | DENV2 | Serum | 19/03/2019 | 19/03/2019 | Female | 58 | Chapadão do Sul | MS | 25 | Dengue with no warning signs |
| **CG124** | DENV2 | Serum | 18/03/2019 | 16/03/2019 | Male | 53 | Nioaque | MS | 26 | Dengue with no warning signs |
| **CG125** | DENV2 | Serum | 15/03/2019 | 14/03/2019 | Female | 16 | Dois Irmãos do Buriti | MS | 24 | Dengue with no warning signs |
| **CG126** | DENV2 | Serum | 20/03/2019 | 16/03/2019 | Male | 24 | Ivinhema | MS | 24 | Dengue with no warning signs |
| **CG128** | DENV2 | Serum | 13/03/2019 | 11/03/2019 | Female | 65 | Maracajú | MS | 23 | Dengue with no warning signs |
| **CG129** | DENV2 | Serum | 11/02/2019 | 10/02/2019 | Female | 14 | Água Clara | MS | 25 | Dengue with no warning signs |
| **CG130** | DENV2 | Serum | 15/03/2019 | 14/03/2019 | Male | 21 | Nioaque | MS | 17 | Dengue with no warning signs |
| **CG132** | DENV2 | Serum | 26/04/2019 | 23/04/2019 | Male | 38 | Campo Grande | MS | 25 | Dengue with no warning signs |
| **CG133** | DENV2 | Serum | 19/03/2019 | 17/03/2019 | Female | 39 | Itaquiraí | MS | 29 | Dengue with no warning signs |
| **CG134** | DENV2 | Serum | 10/01/2019 | 09/01/2019 | Female | 23 | Campo Grande | MS | 26 | Dengue with no warning signs |
| **CB02** | DENV2 | Serum | 18/02/2019 | 15/02/2019 | Male | 36 | Nova Xavantina | MT | 23 | Dengue with no warning signs |
| **CB03** | DENV2 | Serum | 02/03/2019 | 27/02/2019 | Male | 21 | Cuiaba | MT | 31 | Dengue with no warning signs |
| **OPAS 145** | DENV2 | Serum | 09/05/2019 | 09/05/2019 | Male | 45 | Custódia | PE | 28 | Dengue with no warning signs |
| **OPAS 147** | DENV2 | Serum | 31/05/2019 | 27/05/2019 | Female | 34 | Santa Maria da Boa Vista | PE | 27 | Dengue with no warning signs |
| **OPAS 164** | DENV2 | Serum | 27/06/2019 | 23/06/2019 | Female | 8 | Jaboatão dos Guararapes | PE | 29 | Dengue with no warning signs |
| **OPAS 172** | DENV2 | Serum | 25/01/2016 | 28/01/2016 | Male | 25 | Ribeirão Preto | SP | 27 | Dengue with no warning signs |
| **OPAS 176** | DENV2 | Serum | 19/02/2016 | 26/02/2016 | Male | 25 | Ribeirão Preto | SP | 35 | Dengue with no warning signs |
| **OPAS 177** | DENV2 | Serum | 01/03/2016 | 08/03/2016 | Male | 54 | Ribeirão Preto | SP | 28 | Dengue with no warning signs |
| **OPAS 178** | DENV2 | Serum | 14/01/2019 | 11/01/2019 | Female | 38 | Vassouras | RJ | 27 | Dengue with no warning signs |

ID=study identifier; Collection date=Sample collection date; Municipality=Municipality of residence; State=BA-Bahia; DF-Distrito Federal; GO-Goiás; MG-Minas Gerais; MS-Mato Grosso do Sul; MT-Mato Grosso do Sul; PE-Pernambuco; SP-São Paulo; RJ-Rio de Janeiro; Ct=RT-qPCR quantification cycle threshold value; NA=Not Applicable.

**Supplementary Table 3. Sequencing statistics for the 227 DENV1 and DENV2 sequences generated in this study.**

| **ID** | **Serotype** | **Accession Number** | **Reads** | **Coverage (%)** | **GenBank link** |
| --- | --- | --- | --- | --- | --- |
| **OPAS 41** | DENV1 | MT929530 | 1148 | 67.9 | <https://www.ncbi.nlm.nih.gov/nuccore/MT929530> |
| **OPAS 42** | DENV1 | MT929531 | 38409 | 89.5 | <https://www.ncbi.nlm.nih.gov/nuccore/MT929531> |
| **OPAS 43** | DENV1 | MT929532 | 47835 | 89.3 | <https://www.ncbi.nlm.nih.gov/nuccore/MT929532> |
| **OPAS 44** | DENV1 | MT929533 | 32466 | 89.3 | <https://www.ncbi.nlm.nih.gov/nuccore/MT929533> |
| **OPAS 45** | DENV1 | MT929534 | 34796 | 89.3 | <https://www.ncbi.nlm.nih.gov/nuccore/MT929534> |
| **OPAS 46** | DENV1 | MT929535 | 60684 | 89.3 | <https://www.ncbi.nlm.nih.gov/nuccore/MT929535> |
| **OPAS 47** | DENV1 | MT929536 | 60872 | 89.3 | <https://www.ncbi.nlm.nih.gov/nuccore/MT929536> |
| **OPAS 48** | DENV1 | MT929537 | 44516 | 89.3 | <https://www.ncbi.nlm.nih.gov/nuccore/MT929537> |
| **OPAS 49** | DENV1 | MT929538 | 25874 | 85.3 | <https://www.ncbi.nlm.nih.gov/nuccore/MT929538> |
| **OPAS 50** | DENV1 | MT929539 | 17230 | 89.4 | <https://www.ncbi.nlm.nih.gov/nuccore/MT929539> |
| **OPAS 56** | DENV1 | MT929540 | 36579 | 85.3 | <https://www.ncbi.nlm.nih.gov/nuccore/MT929540> |
| **OPAS 57** | DENV1 | MT929541 | 34262 | 89.3 | <https://www.ncbi.nlm.nih.gov/nuccore/MT929541> |
| **OPAS 58** | DENV1 | MT929542 | 13742 | 85.4 | <https://www.ncbi.nlm.nih.gov/nuccore/MT929542> |
| **OPAS 60** | DENV1 | MT929543 | 25105 | 89.3 | <https://www.ncbi.nlm.nih.gov/nuccore/MT929543> |
| **OPAS 61** | DENV1 | MT929544 | 23023 | 89.3 | <https://www.ncbi.nlm.nih.gov/nuccore/MT929544> |
| **OPAS 62** | DENV1 | MT929545 | 33004 | 85.3 | <https://www.ncbi.nlm.nih.gov/nuccore/MT929545> |
| **OPAS 63** | DENV1 | MT929546 | 25770 | 89.3 | <https://www.ncbi.nlm.nih.gov/nuccore/MT929546> |
| **OPAS 64** | DENV1 | MT929547 | 34030 | 89.3 | <https://www.ncbi.nlm.nih.gov/nuccore/MT929547> |
| **OPAS 65** | DENV1 | MT929548 | 30509 | 89.3 | <https://www.ncbi.nlm.nih.gov/nuccore/MT929548> |
| **OPAS 157** | DENV1 | MT929573 | 42518 | 93.6 | <https://www.ncbi.nlm.nih.gov/nuccore/MT929573> |
| **OPAS 158** | DENV1 | MT929574 | 36165 | 93.6 | <https://www.ncbi.nlm.nih.gov/nuccore/MT929574> |
| **GO03** | DENV1 | MT929528 | 22569 | 93.6 | <https://www.ncbi.nlm.nih.gov/nuccore/MT929528> |
| **GO04** | DENV1 | MT929529 | 28354 | 89.6 | <https://www.ncbi.nlm.nih.gov/nuccore/MT929529> |
| **OPAS 131** | DENV1 | MT929556 | 67644 | 93.6 | <https://www.ncbi.nlm.nih.gov/nuccore/MT929556> |
| **OPAS 132** | DENV1 | MT929557 | 86925 | 85.3 | <https://www.ncbi.nlm.nih.gov/nuccore/MT929557> |
| **OPAS 133** | DENV1 | MT929558 | 80673 | 89.6 | <https://www.ncbi.nlm.nih.gov/nuccore/MT929558> |
| **OPAS 134** | DENV1 | MT929559 | 54060 | 89.3 | <https://www.ncbi.nlm.nih.gov/nuccore/MT929559> |
| **OPAS 135** | DENV1 | MT929560 | 81799 | 85.3 | <https://www.ncbi.nlm.nih.gov/nuccore/MT929560> |
| **OPAS 136** | DENV1 | MT929561 | 66262 | 93.6 | <https://www.ncbi.nlm.nih.gov/nuccore/MT929561> |
| **OPAS 137** | DENV1 | MT929562 | 60855 | 85.3 | <https://www.ncbi.nlm.nih.gov/nuccore/MT929562> |
| **OPAS 138** | DENV1 | MT929563 | 70760 | 85.3 | <https://www.ncbi.nlm.nih.gov/nuccore/MT929563> |
| **OPAS 90** | DENV1 | MT929549 | 58872 | 93.6 | <https://www.ncbi.nlm.nih.gov/nuccore/MT929549> |
| **OPAS 92** | DENV1 | MT929550 | 60850 | 94.8 | <https://www.ncbi.nlm.nih.gov/nuccore/MT929550> |
| **OPAS 99** | DENV1 | MT929551 | 74959 | 78.2 | <https://www.ncbi.nlm.nih.gov/nuccore/MT929551> |
| **OPAS 100** | DENV1 | MT929552 | 66474 | 86.0 | <https://www.ncbi.nlm.nih.gov/nuccore/MT929552> |
| **OPAS 101** | DENV1 | MT929553 | 82037 | 80.8 | <https://www.ncbi.nlm.nih.gov/nuccore/MT929553> |
| **OPAS 102** | DENV1 | MT929554 | 81787 | 94.1 | <https://www.ncbi.nlm.nih.gov/nuccore/MT929554> |
| **OPAS 103** | DENV1 | MT929555 | 73129 | 89.3 | <https://www.ncbi.nlm.nih.gov/nuccore/MT929555> |
| **OPAS 165** | DENV1 | MT929577 | 38272 | 93.6 | <https://www.ncbi.nlm.nih.gov/nuccore/MT929577> |
| **OPAS 167** | DENV1 | MT929578 | 44365 | 93.7 | <https://www.ncbi.nlm.nih.gov/nuccore/MT929578> |
| **OPAS 168** | DENV1 | MT929579 | 41920 | 93.6 | <https://www.ncbi.nlm.nih.gov/nuccore/MT929579> |
| **OPAS 169** | DENV1 | MT929580 | 37728 | 93.6 | <https://www.ncbi.nlm.nih.gov/nuccore/MT929580> |
| **OPAS 148** | DENV1 | MT929564 | 73395 | 89.6 | <https://www.ncbi.nlm.nih.gov/nuccore/MT929564> |
| **OPAS 149** | DENV1 | MT929565 | 74685 | 70.2 | <https://www.ncbi.nlm.nih.gov/nuccore/MT929565> |
| **OPAS 150** | DENV1 | MT929566 | 80594 | 75.1 | <https://www.ncbi.nlm.nih.gov/nuccore/MT929566> |
| **OPAS 151** | DENV1 | MT929567 | 29271 | 82.0 | <https://www.ncbi.nlm.nih.gov/nuccore/MT929567> |
| **OPAS 152** | DENV1 | MT929568 | 79935 | 85.3 | <https://www.ncbi.nlm.nih.gov/nuccore/MT929568> |
| **OPAS 153** | DENV1 | MT929569 | 45364 | 82.6 | <https://www.ncbi.nlm.nih.gov/nuccore/MT929569> |
| **OPAS 154** | DENV1 | MT929570 | 75476 | 85.3 | <https://www.ncbi.nlm.nih.gov/nuccore/MT929570> |
| **OPAS 155** | DENV1 | MT929571 | 30379 | 91.9 | <https://www.ncbi.nlm.nih.gov/nuccore/MT929571> |
| **OPAS 156** | DENV1 | MT929572 | 126739 | 85.3 | <https://www.ncbi.nlm.nih.gov/nuccore/MT929572> |
| **OPAS 162** | DENV1 | MT929575 | 26821 | 85.3 | <https://www.ncbi.nlm.nih.gov/nuccore/MT929575> |
| **OPAS 163** | DENV1 | MT929576 | 236906 | 77.5 | <https://www.ncbi.nlm.nih.gov/nuccore/MT929576> |
| **OPAS 171** | DENV1 | MT929581 | 31506 | 78.5 | <https://www.ncbi.nlm.nih.gov/nuccore/MT929581> |
| **OPAS 173** | DENV1 | MT929582 | 9180 | 74.3 | <https://www.ncbi.nlm.nih.gov/nuccore/MT929582> |
| **OPAS 174** | DENV1 | MT929583 | 34410 | 78.5 | <https://www.ncbi.nlm.nih.gov/nuccore/MT929583> |
| **OPAS 175** | DENV1 | MT929584 | 23241 | 94.0 | <https://www.ncbi.nlm.nih.gov/nuccore/MT929584> |
| **OPAS 51** | DENV2 | MT929688 | 22430 | 93.5 | <https://www.ncbi.nlm.nih.gov/nuccore/MT929688> |
| **OPAS 52** | DENV2 | MT929689 | 26105 | 84.6 | <https://www.ncbi.nlm.nih.gov/nuccore/MT929689> |
| **OPAS 53** | DENV2 | MT929690 | 24082 | 93.5 | <https://www.ncbi.nlm.nih.gov/nuccore/MT929690> |
| **OPAS 54** | DENV2 | MT929691 | 20538 | 88.2 | <https://www.ncbi.nlm.nih.gov/nuccore/MT929691> |
| **OPAS 55** | DENV2 | MT929692 | 25194 | 88.2 | <https://www.ncbi.nlm.nih.gov/nuccore/MT929692> |
| **OPAS 110** | DENV2 | MT929722 | 78479 | 93.5 | <https://www.ncbi.nlm.nih.gov/nuccore/MT929722> |
| **OPAS 111** | DENV2 | MT929723 | 127120 | 93.5 | <https://www.ncbi.nlm.nih.gov/nuccore/MT929723> |
| **OPAS 112** | DENV2 | MT929724 | 75160 | 77.7 | <https://www.ncbi.nlm.nih.gov/nuccore/MT929724> |
| **OPAS 113** | DENV2 | MT929725 | 89452 | 93.5 | <https://www.ncbi.nlm.nih.gov/nuccore/MT929725> |
| **OPAS 114** | DENV2 | MT929726 | 80250 | 85.2 | <https://www.ncbi.nlm.nih.gov/nuccore/MT929726> |
| **OPAS 115** | DENV2 | MT929727 | 96331 | 73.5 | <https://www.ncbi.nlm.nih.gov/nuccore/MT929727> |
| **OPAS 116** | DENV2 | MT929728 | 76730 | 93.5 | <https://www.ncbi.nlm.nih.gov/nuccore/MT929728> |
| **OPAS 117** | DENV2 | MT929729 | 74632 | 93.5 | <https://www.ncbi.nlm.nih.gov/nuccore/MT929729> |
| **OPAS 118** | DENV2 | MT929730 | 74663 | 88.1 | <https://www.ncbi.nlm.nih.gov/nuccore/MT929730> |
| **OPAS 119** | DENV2 | MT929731 | 71196 | 93.5 | <https://www.ncbi.nlm.nih.gov/nuccore/MT929731> |
| **OPAS 120** | DENV2 | MT929732 | 69618 | 93.5 | <https://www.ncbi.nlm.nih.gov/nuccore/MT929732> |
| **OPAS 121** | DENV2 | MT929733 | 73302 | 88.1 | <https://www.ncbi.nlm.nih.gov/nuccore/MT929733> |
| **OPAS 122** | DENV2 | MT929734 | 76102 | 88.1 | <https://www.ncbi.nlm.nih.gov/nuccore/MT929734> |
| **OPAS 123** | DENV2 | MT929735 | 62619 | 93.5 | <https://www.ncbi.nlm.nih.gov/nuccore/MT929735> |
| **OPAS 124** | DENV2 | MT929736 | 78826 | 80.9 | <https://www.ncbi.nlm.nih.gov/nuccore/MT929736> |
| **OPAS 161** | DENV2 | MT929749 | 11924 | 69.5 | <https://www.ncbi.nlm.nih.gov/nuccore/MT929749> |
| **GO72** | DENV2 | MT929646 | 33490 | 88.1 | <https://www.ncbi.nlm.nih.gov/nuccore/MT929646> |
| **GO75** | DENV2 | MT929647 | 29201 | 92.1 | <https://www.ncbi.nlm.nih.gov/nuccore/MT929647> |
| **OPAS 66** | DENV2 | MT929693 | 25861 | 93.5 | <https://www.ncbi.nlm.nih.gov/nuccore/MT929693> |
| **OPAS 67** | DENV2 | MT929694 | 53737 | 93.7 | <https://www.ncbi.nlm.nih.gov/nuccore/MT929694> |
| **OPAS 68** | DENV2 | MT929695 | 76127 | 93.5 | <https://www.ncbi.nlm.nih.gov/nuccore/MT929695> |
| **OPAS 69** | DENV2 | MT929696 | 77345 | 93.5 | <https://www.ncbi.nlm.nih.gov/nuccore/MT929696> |
| **OPAS 70** | DENV2 | MT929697 | 53087 | 93.5 | <https://www.ncbi.nlm.nih.gov/nuccore/MT929697> |
| **OPAS 71** | DENV2 | MT929698 | 68171 | 93.5 | <https://www.ncbi.nlm.nih.gov/nuccore/MT929698> |
| **OPAS 72** | DENV2 | MT929699 | 62398 | 76.9 | <https://www.ncbi.nlm.nih.gov/nuccore/MT929699> |
| **OPAS 74** | DENV2 | MT929700 | 69451 | 93.0 | <https://www.ncbi.nlm.nih.gov/nuccore/MT929700> |
| **OPAS 75** | DENV2 | MT929701 | 59345 | 93.5 | <https://www.ncbi.nlm.nih.gov/nuccore/MT929701> |
| **OPAS 76** | DENV2 | MT929702 | 61310 | 93.5 | <https://www.ncbi.nlm.nih.gov/nuccore/MT929702> |
| **OPAS 77** | DENV2 | MT929703 | 52796 | 93.5 | <https://www.ncbi.nlm.nih.gov/nuccore/MT929703> |
| **OPAS 78** | DENV2 | MT929704 | 53603 | 93.5 | <https://www.ncbi.nlm.nih.gov/nuccore/MT929704> |
| **OPAS 79** | DENV2 | MT929705 | 72112 | 93.5 | <https://www.ncbi.nlm.nih.gov/nuccore/MT929705> |
| **OPAS 80** | DENV2 | MT929706 | 58137 | 93.0 | <https://www.ncbi.nlm.nih.gov/nuccore/MT929706> |
| **OPAS 81** | DENV2 | MT929707 | 55335 | 93.5 | <https://www.ncbi.nlm.nih.gov/nuccore/MT929707> |
| **OPAS 82** | DENV2 | MT929708 | 66133 | 93.5 | <https://www.ncbi.nlm.nih.gov/nuccore/MT929708> |
| **OPAS 83** | DENV2 | MT929709 | 63357 | 93.5 | <https://www.ncbi.nlm.nih.gov/nuccore/MT929709> |
| **OPAS 84** | DENV2 | MT929710 | 79443 | 93.5 | <https://www.ncbi.nlm.nih.gov/nuccore/MT929710> |
| **OPAS 85** | DENV2 | MT929711 | 59880 | 93.5 | <https://www.ncbi.nlm.nih.gov/nuccore/MT929711> |
| **OPAS 86** | DENV2 | MT929712 | 82765 | 93.5 | <https://www.ncbi.nlm.nih.gov/nuccore/MT929712> |
| **OPAS 87** | DENV2 | MT929713 | 73573 | 93.5 | <https://www.ncbi.nlm.nih.gov/nuccore/MT929713> |
| **OPAS 89** | DENV2 | MT929714 | 91026 | 93.5 | <https://www.ncbi.nlm.nih.gov/nuccore/MT929714> |
| **OPAS 125** | DENV2 | MT929737 | 67578 | 88.2 | <https://www.ncbi.nlm.nih.gov/nuccore/MT929737> |
| **OPAS 126** | DENV2 | MT929738 | 20234 | 69.8 | <https://www.ncbi.nlm.nih.gov/nuccore/MT929738> |
| **OPAS 127** | DENV2 | MT929739 | 14215 | 79.2 | <https://www.ncbi.nlm.nih.gov/nuccore/MT929739> |
| **OPAS 128** | DENV2 | MT929740 | 24790 | 82.4 | <https://www.ncbi.nlm.nih.gov/nuccore/MT929740> |
| **OPAS 129** | DENV2 | MT929741 | 35067 | 93.5 | <https://www.ncbi.nlm.nih.gov/nuccore/MT929741> |
| **OPAS 130** | DENV2 | MT929742 | 33004 | 88.1 | <https://www.ncbi.nlm.nih.gov/nuccore/MT929742> |
| **OPAS 139** | DENV2 | MT929743 | 36363 | 93.0 | <https://www.ncbi.nlm.nih.gov/nuccore/MT929743> |
| **OPAS 140** | DENV2 | MT929744 | 13891 | 70.7 | <https://www.ncbi.nlm.nih.gov/nuccore/MT929744> |
| **OPAS 142** | DENV2 | MT929745 | 9452 | 83.0 | <https://www.ncbi.nlm.nih.gov/nuccore/MT929745> |
| **OPAS 159** | DENV2 | MT929748 | 16501 | 75.3 | <https://www.ncbi.nlm.nih.gov/nuccore/MT929748> |
| **OPAS 01** | DENV2 | MT929648 | 67212 | 86.1 | <https://www.ncbi.nlm.nih.gov/nuccore/MT929648> |
| **OPAS 02** | DENV2 | MT929649 | 79407 | 88.0 | <https://www.ncbi.nlm.nih.gov/nuccore/MT929649> |
| **OPAS 03** | DENV2 | MT929650 | 64014 | 87.6 | <https://www.ncbi.nlm.nih.gov/nuccore/MT929650> |
| **OPAS 04** | DENV2 | MT929651 | 64749 | 93.5 | <https://www.ncbi.nlm.nih.gov/nuccore/MT929651> |
| **OPAS 05** | DENV2 | MT929652 | 54742 | 88.2 | <https://www.ncbi.nlm.nih.gov/nuccore/MT929652> |
| **OPAS 06** | DENV2 | MT929653 | 55680 | 93.5 | <https://www.ncbi.nlm.nih.gov/nuccore/MT929653> |
| **OPAS 07** | DENV2 | MT929654 | 66199 | 91.4 | <https://www.ncbi.nlm.nih.gov/nuccore/MT929654> |
| **OPAS 08** | DENV2 | MT929655 | 79984 | 86.6 | <https://www.ncbi.nlm.nih.gov/nuccore/MT929655> |
| **OPAS 09** | DENV2 | MT929656 | 51403 | 93.5 | <https://www.ncbi.nlm.nih.gov/nuccore/MT929656> |
| **OPAS 10** | DENV2 | MT929657 | 64173 | 88.2 | <https://www.ncbi.nlm.nih.gov/nuccore/MT929657> |
| **OPAS 11** | DENV2 | MT929658 | 40468 | 93.5 | <https://www.ncbi.nlm.nih.gov/nuccore/MT929658> |
| **OPAS 12** | DENV2 | MT929659 | 63797 | 93.5 | <https://www.ncbi.nlm.nih.gov/nuccore/MT929659> |
| **OPAS 13** | DENV2 | MT929660 | 61680 | 93.5 | <https://www.ncbi.nlm.nih.gov/nuccore/MT929660> |
| **OPAS 14** | DENV2 | MT929661 | 55153 | 93.5 | <https://www.ncbi.nlm.nih.gov/nuccore/MT929661> |
| **OPAS 15** | DENV2 | MT929662 | 49508 | 90.1 | <https://www.ncbi.nlm.nih.gov/nuccore/MT929662> |
| **OPAS 16** | DENV2 | MT929663 | 61994 | 93.5 | <https://www.ncbi.nlm.nih.gov/nuccore/MT929663> |
| **OPAS 17** | DENV2 | MT929664 | 37366 | 93.5 | <https://www.ncbi.nlm.nih.gov/nuccore/MT929664> |
| **OPAS 18** | DENV2 | MT929665 | 34881 | 66.3 | <https://www.ncbi.nlm.nih.gov/nuccore/MT929665> |
| **OPAS 19** | DENV2 | MT929666 | 46152 | 93.5 | <https://www.ncbi.nlm.nih.gov/nuccore/MT929666> |
| **OPAS 20** | DENV2 | MT929667 | 64056 | 93.5 | <https://www.ncbi.nlm.nih.gov/nuccore/MT929667> |
| **OPAS 21** | DENV2 | MT929668 | 42803 | 93.5 | <https://www.ncbi.nlm.nih.gov/nuccore/MT929668> |
| **OPAS 22** | DENV2 | MT929669 | 51459 | 93.5 | <https://www.ncbi.nlm.nih.gov/nuccore/MT929669> |
| **OPAS 23** | DENV2 | MT929670 | 49658 | 92.9 | <https://www.ncbi.nlm.nih.gov/nuccore/MT929670> |
| **OPAS 24** | DENV2 | MT929671 | 13142 | 60.2 | <https://www.ncbi.nlm.nih.gov/nuccore/MT929671> |
| **OPAS 25** | DENV2 | MT929672 | 9737 | 93.5 | <https://www.ncbi.nlm.nih.gov/nuccore/MT929672> |
| **OPAS 26** | DENV2 | MT929673 | 10315 | 93.5 | <https://www.ncbi.nlm.nih.gov/nuccore/MT929673> |
| **OPAS 27** | DENV2 | MT929674 | 13660 | 73.5 | <https://www.ncbi.nlm.nih.gov/nuccore/MT929674> |
| **OPAS 28** | DENV2 | MT929675 | 11857 | 89.1 | <https://www.ncbi.nlm.nih.gov/nuccore/MT929675> |
| **OPAS 29** | DENV2 | MT929676 | 9867 | 93.5 | <https://www.ncbi.nlm.nih.gov/nuccore/MT929676> |
| **OPAS 30** | DENV2 | MT929677 | 11290 | 93.5 | <https://www.ncbi.nlm.nih.gov/nuccore/MT929677> |
| **OPAS 31** | DENV2 | MT929678 | 12548 | 73.5 | <https://www.ncbi.nlm.nih.gov/nuccore/MT929678> |
| **OPAS 32** | DENV2 | MT929679 | 7967 | 88.1 | <https://www.ncbi.nlm.nih.gov/nuccore/MT929679> |
| **OPAS 33** | DENV2 | MT929680 | 11544 | 88.2 | <https://www.ncbi.nlm.nih.gov/nuccore/MT929680> |
| **OPAS 34** | DENV2 | MT929681 | 13239 | 93.0 | <https://www.ncbi.nlm.nih.gov/nuccore/MT929681> |
| **OPAS 35** | DENV2 | MT929682 | 11617 | 89.9 | <https://www.ncbi.nlm.nih.gov/nuccore/MT929682> |
| **OPAS 36** | DENV2 | MT929683 | 19614 | 73.5 | <https://www.ncbi.nlm.nih.gov/nuccore/MT929683> |
| **OPAS 37** | DENV2 | MT929684 | 16382 | 88.8 | <https://www.ncbi.nlm.nih.gov/nuccore/MT929684> |
| **OPAS 38** | DENV2 | MT929685 | 14810 | 93.5 | <https://www.ncbi.nlm.nih.gov/nuccore/MT929685> |
| **OPAS 39** | DENV2 | MT929686 | 18506 | 83.8 | <https://www.ncbi.nlm.nih.gov/nuccore/MT929686> |
| **OPAS 40** | DENV2 | MT929687 | 16077 | 66.8 | <https://www.ncbi.nlm.nih.gov/nuccore/MT929687> |
| **OPAS 93** | DENV2 | MT929715 | 67656 | 88.3 | <https://www.ncbi.nlm.nih.gov/nuccore/MT929715> |
| **OPAS 95** | DENV2 | MT929716 | 81051 | 93.5 | <https://www.ncbi.nlm.nih.gov/nuccore/MT929716> |
| **OPAS 96** | DENV2 | MT929717 | 86395 | 88.2 | <https://www.ncbi.nlm.nih.gov/nuccore/MT929717> |
| **OPAS 105** | DENV2 | MT929718 | 85629 | 86.9 | <https://www.ncbi.nlm.nih.gov/nuccore/MT929718> |
| **OPAS 106** | DENV2 | MT929719 | 71717 | 93.5 | <https://www.ncbi.nlm.nih.gov/nuccore/MT929719> |
| **OPAS 107** | DENV2 | MT929720 | 72564 | 93.5 | <https://www.ncbi.nlm.nih.gov/nuccore/MT929720> |
| **OPAS 108** | DENV2 | MT929721 | 84260 | 88.1 | <https://www.ncbi.nlm.nih.gov/nuccore/MT929721> |
| **CG02** | DENV2 | MT929587 | 58997 | 93.6 | <https://www.ncbi.nlm.nih.gov/nuccore/MT929587> |
| **CG06** | DENV2 | MT929588 | 70195 | 93.6 | <https://www.ncbi.nlm.nih.gov/nuccore/MT929588> |
| **CG07** | DENV2 | MT929589 | 41807 | 93.6 | <https://www.ncbi.nlm.nih.gov/nuccore/MT929589> |
| **CG08** | DENV2 | MT929590 | 65721 | 67.3 | <https://www.ncbi.nlm.nih.gov/nuccore/MT929590> |
| **CG09** | DENV2 | MT929591 | 47818 | 93.6 | <https://www.ncbi.nlm.nih.gov/nuccore/MT929591> |
| **CG10** | DENV2 | MT929592 | 49514 | 73.8 | <https://www.ncbi.nlm.nih.gov/nuccore/MT929592> |
| **CG11** | DENV2 | MT929593 | 42961 | 93.5 | <https://www.ncbi.nlm.nih.gov/nuccore/MT929593> |
| **CG12** | DENV2 | MT929594 | 21079 | 73.7 | <https://www.ncbi.nlm.nih.gov/nuccore/MT929594> |
| **CG13** | DENV2 | MT929595 | 34494 | 93.6 | <https://www.ncbi.nlm.nih.gov/nuccore/MT929595> |
| **CG14** | DENV2 | MT929596 | 54053 | 93.6 | <https://www.ncbi.nlm.nih.gov/nuccore/MT929596> |
| **CG15** | DENV2 | MT929597 | 53455 | 79.1 | <https://www.ncbi.nlm.nih.gov/nuccore/MT929597> |
| **CG16** | DENV2 | MT929598 | 27224 | 93.6 | <https://www.ncbi.nlm.nih.gov/nuccore/MT929598> |
| **CG17** | DENV2 | MT929599 | 49155 | 93.6 | <https://www.ncbi.nlm.nih.gov/nuccore/MT929599> |
| **CG18** | DENV2 | MT929600 | 43608 | 73.9 | <https://www.ncbi.nlm.nih.gov/nuccore/MT929600> |
| **CG19** | DENV2 | MT929601 | 27042 | 82.0 | <https://www.ncbi.nlm.nih.gov/nuccore/MT929601> |
| **CG20** | DENV2 | MT929602 | 43832 | 73.7 | <https://www.ncbi.nlm.nih.gov/nuccore/MT929602> |
| **CG21** | DENV2 | MT929603 | 22422 | 93.6 | <https://www.ncbi.nlm.nih.gov/nuccore/MT929603> |
| **CG22** | DENV2 | MT929604 | 21798 | 73.6 | <https://www.ncbi.nlm.nih.gov/nuccore/MT929604> |
| **CG23** | DENV2 | MT929605 | 42692 | 93.6 | <https://www.ncbi.nlm.nih.gov/nuccore/MT929605> |
| **CG24** | DENV2 | MT929606 | 37019 | 93.6 | <https://www.ncbi.nlm.nih.gov/nuccore/MT929606> |
| **CG68** | DENV2 | MT929607 | 101708 | 83.1 | <https://www.ncbi.nlm.nih.gov/nuccore/MT929607> |
| **CG69** | DENV2 | MT929608 | 72620 | 88.1 | <https://www.ncbi.nlm.nih.gov/nuccore/MT929608> |
| **CG70** | DENV2 | MT929609 | 116881 | 84.6 | <https://www.ncbi.nlm.nih.gov/nuccore/MT929609> |
| **CG71** | DENV2 | MT929610 | 5307 | 88.1 | <https://www.ncbi.nlm.nih.gov/nuccore/MT929610> |
| **CG81** | DENV2 | MT929611 | 166110 | 88.2 | <https://www.ncbi.nlm.nih.gov/nuccore/MT929611> |
| **CG86** | DENV2 | MT929612 | 114276 | 93.5 | <https://www.ncbi.nlm.nih.gov/nuccore/MT929612> |
| **CG87** | DENV2 | MT929613 | 4710 | 88.1 | <https://www.ncbi.nlm.nih.gov/nuccore/MT929613> |
| **CG90** | DENV2 | MT929614 | 103971 | 93.5 | <https://www.ncbi.nlm.nih.gov/nuccore/MT929614> |
| **CG94** | DENV2 | MT929615 | 134892 | 80.9 | <https://www.ncbi.nlm.nih.gov/nuccore/MT929615> |
| **CG96** | DENV2 | MT929616 | 148786 | 93.5 | <https://www.ncbi.nlm.nih.gov/nuccore/MT929616> |
| **CG99** | DENV2 | MT929617 | 120232 | 93.5 | <https://www.ncbi.nlm.nih.gov/nuccore/MT929617> |
| **CG100** | DENV2 | MT929618 | 116370 | 93.5 | <https://www.ncbi.nlm.nih.gov/nuccore/MT929618> |
| **CG102** | DENV2 | MT929619 | 55098 | 93.5 | <https://www.ncbi.nlm.nih.gov/nuccore/MT929619> |
| **CG103** | DENV2 | MT929620 | 50051 | 88.2 | <https://www.ncbi.nlm.nih.gov/nuccore/MT929620> |
| **CG104** | DENV2 | MT929621 | 5237 | 88.1 | <https://www.ncbi.nlm.nih.gov/nuccore/MT929621> |
| **CG105** | DENV2 | MT929622 | 30959 | 93.5 | <https://www.ncbi.nlm.nih.gov/nuccore/MT929622> |
| **CG106** | DENV2 | MT929623 | 110736 | 93.5 | <https://www.ncbi.nlm.nih.gov/nuccore/MT929623> |
| **CG107** | DENV2 | MT929624 | 1001632 | 73.5 | <https://www.ncbi.nlm.nih.gov/nuccore/MT929624> |
| **CG108** | DENV2 | MT929625 | 88602 | 88.4 | <https://www.ncbi.nlm.nih.gov/nuccore/MT929625> |
| **CG109** | DENV2 | MT929626 | 57723 | 93.5 | <https://www.ncbi.nlm.nih.gov/nuccore/MT929626> |
| **CG110** | DENV2 | MT929627 | 82777 | 88.2 | <https://www.ncbi.nlm.nih.gov/nuccore/MT929627> |
| **CG111** | DENV2 | MT929628 | 72903 | 88.3 | <https://www.ncbi.nlm.nih.gov/nuccore/MT929628> |
| **CG112** | DENV2 | MT929629 | 59051 | 88.2 | <https://www.ncbi.nlm.nih.gov/nuccore/MT929629> |
| **CG116** | DENV2 | MT929630 | 48670 | 87.8 | <https://www.ncbi.nlm.nih.gov/nuccore/MT929630> |
| **CG117** | DENV2 | MT929631 | 43758 | 88.1 | <https://www.ncbi.nlm.nih.gov/nuccore/MT929631> |
| **CG119** | DENV2 | MT929632 | 66890 | 93.5 | <https://www.ncbi.nlm.nih.gov/nuccore/MT929632> |
| **CG120** | DENV2 | MT929633 | 50837 | 88.3 | <https://www.ncbi.nlm.nih.gov/nuccore/MT929633> |
| **CG121** | DENV2 | MT929634 | 123428 | 88.2 | <https://www.ncbi.nlm.nih.gov/nuccore/MT929634> |
| **CG122** | DENV2 | MT929635 | 150786 | 88.1 | <https://www.ncbi.nlm.nih.gov/nuccore/MT929635> |
| **CG123** | DENV2 | MT929636 | 98764 | 88.1 | <https://www.ncbi.nlm.nih.gov/nuccore/MT929636> |
| **CG124** | DENV2 | MT929637 | 19138 | 88.2 | <https://www.ncbi.nlm.nih.gov/nuccore/MT929637> |
| **CG125** | DENV2 | MT929638 | 44351 | 85.8 | <https://www.ncbi.nlm.nih.gov/nuccore/MT929638> |
| **CG126** | DENV2 | MT929639 | 26287 | 73.7 | <https://www.ncbi.nlm.nih.gov/nuccore/MT929639> |
| **CG128** | DENV2 | MT929640 | 38678 | 87.9 | <https://www.ncbi.nlm.nih.gov/nuccore/MT929640> |
| **CG129** | DENV2 | MT929641 | 45603 | 88.1 | <https://www.ncbi.nlm.nih.gov/nuccore/MT929641> |
| **CG130** | DENV2 | MT929642 | 39445 | 93.5 | <https://www.ncbi.nlm.nih.gov/nuccore/MT929642> |
| **CG132** | DENV2 | MT929643 | 39587 | 87.0 | <https://www.ncbi.nlm.nih.gov/nuccore/MT929643> |
| **CG133** | DENV2 | MT929644 | 83438 | 88.2 | <https://www.ncbi.nlm.nih.gov/nuccore/MT929644> |
| **CG134** | DENV2 | MT929645 | 45255 | 88.1 | <https://www.ncbi.nlm.nih.gov/nuccore/MT929645> |
| **CB02** | DENV2 | MT929585 | 36474 | 51.3 | <https://www.ncbi.nlm.nih.gov/nuccore/MT929585> |
| **CB03** | DENV2 | MT929586 | 32227 | 47.8 | <https://www.ncbi.nlm.nih.gov/nuccore/MT929586> |
| **OPAS 145** | DENV2 | MT929746 | 85028 | 74.3 | <https://www.ncbi.nlm.nih.gov/nuccore/MT929746> |
| **OPAS 147** | DENV2 | MT929747 | 62082 | 73.5 | <https://www.ncbi.nlm.nih.gov/nuccore/MT929747> |
| **OPAS 164** | DENV2 | MT929750 | 16602 | 79.5 | <https://www.ncbi.nlm.nih.gov/nuccore/MT929750> |
| **OPAS 172** | DENV2 | MT929751 | 21448 | 93.7 | <https://www.ncbi.nlm.nih.gov/nuccore/MT929751> |
| **OPAS 176** | DENV2 | MT929752 | 3243 | 74.4 | <https://www.ncbi.nlm.nih.gov/nuccore/MT929752> |
| **OPAS 177** | DENV2 | MT929753 | 154641 | 94.1 | <https://www.ncbi.nlm.nih.gov/nuccore/MT929753> |
| **OPAS 178** | DENV2 | MT929754 | 23384 | 89.1 | <https://www.ncbi.nlm.nih.gov/nuccore/MT929754> |

ID=study identifier; Accession Number=NCBI accession number

**Supplementary Table 4. Genetic signatures for BR-4 lineages I and II.**

| **SNV** | **nn position** | **Protein** | **nn position** | **aa position** | **aa**  **LinI** | **aa**  **LinII** | **codon LinI** | **codon LinII** |
| --- | --- | --- | --- | --- | --- | --- | --- | --- |
| **1** | 450 | membrane glycoprotein precursor prM | 12 | 4 | T | T | acc | act |
| **2** | 954 | envelope protein E | 18 | 6 | I | I | ata | atc |
| **3** | 1986 | envelope protein E | 1050 | 350 | R | R | cgc | cgc |
| **4** | 2223 | envelope protein E | 1287 | 429 | F | F | ttt | ttc |
| **5** | 2276 | envelope protein E | 1340 | 447 | V | A | gtt | gct |
| **6** | 2319 | envelope protein E | 1383 | 461 | V | V | gtc | gta |
| **7** | 2448 | nonstructural protein NS1 | 27 | 9 | K | K | aaa | aag |
| **8** | 3330 | nonstructural protein NS1 | 909 | 303 | A | A | gcc | gct |
| **9** | 4341 | nonstructural protein NS2B | 210 | 70 | S | S | agt | agc |
| **10** | 4482 | nonstructural protein NS2B | 351 | 117 | T | T | acg | aca |
| **11** | 4974 | nonstructural protein NS3 | 453 | 151 | G | G | ggt | ggc |
| **12** | 5190 | nonstructural protein NS3 | 669 | 223 | P | P | ccc | cct |
| **13** | 5304 | nonstructural protein NS3 | 783 | 261 | C | C | tgt | tgc |
| **14** | 5730 | nonstructural protein NS3 | 1209 | 403 | D | D | gat | gac |
| **15** | 5835 | nonstructural protein NS3 | 1314 | 438 | E | E | gaa | gag |
| **16** | 6966 | nonstructural protein NS4B | 141 | 47 | F | F | ttt | ttt |
| **17** | 7038 | nonstructural protein NS4B | 213 | 71 | Q | Q | cag | caa |
| **18** | 7059 | nonstructural protein NS4B | 234 | 78 | L | L | ctt | ctc |
| **19** | 7137 | nonstructural protein NS4B | 312 | 104 | P | P | cct | ccc |
| **20** | 7656 | RNA-dependent RNA polymerase NS5 | 87 | 29 | K | K | aag | aag |
| **21** | 7758 | RNA-dependent RNA polymerase NS5 | 189 | 63 | R | R | agg | aga |
| **22** | 7999 | RNA-dependent RNA polymerase NS5 | 430 | 144 | L | L | ctg | ttg |
| **23** | 8298 | RNA-dependent RNA polymerase NS5 | 729 | 243 | F | F | ttc | ttt |
| **24** | 8658 | RNA-dependent RNA polymerase NS5 | 1089 | 363 | T | T | act | acc |
| **25** | 9208 | RNA-dependent RNA polymerase NS5 | 1639 | 547 | L | L | tta | cta |
| **26** | 9226 | RNA-dependent RNA polymerase NS5 | 1657 | 553 | V | I | gta | ata |
| **27** | 9303 | RNA-dependent RNA polymerase NS5 | 1734 | 578 | V | V | gtg | gtg |
| **28** | 9399 | RNA-dependent RNA polymerase NS5 | 1830 | 610 | N | N | aat | aac |
| **29** | 9424 | RNA-dependent RNA polymerase NS5 | 1855 | 619 | L | L | tta | cta |
| **30** | 9576 | RNA-dependent RNA polymerase NS5 | 2007 | 669 | P | P | cct | ccc |
| **31** | 9660 | RNA-dependent RNA polymerase NS5 | 2091 | 697 | S | S | tct | tca |
| **32** | 9725 | RNA-dependent RNA polymerase NS5 | 2156 | 719 | I | K | ata | aaa |
| **33** | 9795 | RNA-dependent RNA polymerase NS5 | 2226 | 742 | Q | Q | cag | caa |
| **34** | 9942 | RNA-dependent RNA polymerase NS5 | 2373 | 791 | S | S | agc | agt |

SNV=single nucleotide variant; nn=nucleotide; aa=amino acid; LinI=BR-4 lineage I; LinII=BR-4 lineage II

**Supplementary Table 5. Primers for sequencing the complete genomes of DENV1 and DENV2**

| **Primer Name** | **Sequence (5'-3')^*^** |
| --- | --- |
| DENV1_1_LEFT | AATATGCTGAAACGCGCGAGAA |
| DENV1_1_RIGHT | CCGTCTTCAAGAGTTCAATGTCCA |
| DENV1_2_LEFT | ACCCAGGATTCACGGTGATAGC |
| DENV1_2_RIGHT | ACCAGCAAATCTTGTCTGTTCCA |
| DENV1_3_LEFT | GGAAATACAGCTGACCGACTACG |
| DENV1_3_RIGHT | ACTGCAATGCACGTCATCGAAA |
| DENV1_4_LEFT | CAAGAAAGGAAGCAGCATAGGGA |
| DENV1_4_RIGHT | TTGATGGCAGCTGACATTAGCC |
| DENV1_5_LEFT | TGGAACATTTGGGAAGTTGAGGAC |
| DENV1_5_RIGHT | ACTTCTCTGGATGTTAGTCTGCG |
| DENV1_6_LEFT | TGGATGAACATTGTGGAAATCGAGG |
| DENV1_6_RIGHT | GCATGCCTCCAGCTATTAGTGG |
| DENV1_7_LEFT | AGTTGGCCCCTCAATGAAGGAA |
| DENV1_7_RIGHT | GCACTGACGTAGGTTCCACTTG |
| DENV1_8_LEFT | TCTCATATGGAGGAGGTTGGAGG |
| DENV1_8_RIGHT | AGCCTGAGTTCCATGATCTCTCA |
| DENV1_9_LEFT | ATAGCGGCCAGAGGGTACATCT |
| DENV1_9_RIGHT | TGTTCTCCTCCAACACCTGGTT |
| DENV1_10_LEFT | AAAGAGTGCAGCAATAGACGGG |
| DENV1_10_RIGHT | ATAGAGGGTCCAGGCTGAAGCT |
| DENV1_11_LEFT | TGTGGTGATAGGTTTGTTATTCATGATACT |
| DENV1_11_RIGHT | CTTTGGCTTCGGATCTGTCCAC |
| DENV1_12_LEFT | GGGAAACACTGGGAGAGAAATGG |
| DENV1_12_RIGHT | TGATCCTGATGGCTTGACCTCA |
| DENV1_13_LEFT | TGGAGCAAATGCAAAGAAAACATGG |
| DENV1_13_RIGHT | TGCACGACTTCCTTTTGCCTTT |
| DENV1_14_LEFT | ACTCAGCAAAAGAAGCAGTGGA |
| DENV1_14_RIGHT | GCATGGCACCACTATTTCCCTC |
| DENV1_15_LEFT | AGACGTGACCAGAGAGGAAGTG |
| DENV1_15_RIGHT | TCACTTGGTTTATGGCCACTTGT |
| DENV2_1_LEFT | AGCAGATCTCTGATGAATAACCAACG |
| DENV2_1_RIGHT | TTTTTGCCATCGTCGTCACACA |
| DENV2_2_LEFT | TCGCTCCTTCAATGACAATGCG |
| DENV2_2_RIGHT | CCATTCTCAGCCTGCACTTGAG |
| DENV2_3_LEFT | ACATTGGTCACTTTCAAAAATCCCC |
| DENV2_3_RIGHT | TGAAGGGGATTCTGGTTGGAACT |
| DENV2_4_LEFT | ATAGTGGTTGCGTTGTGAGCTG |
| DENV2_4_RIGHT | CGGCAGCACCATTCTGTTATGA |
| DENV2_5_LEFT | TCATGCAGGCAGGAAAACGATC |
| DENV2_5_RIGHT | TCTCAAGAGTAGTCCAGCTGCA |
| DENV2_6_LEFT | TGGAAATCAGACCATTGAAAGAGAAAGA |
| DENV2_6_RIGHT | TGGTCAGTGTTTGTTCTTCCTCTT |
| DENV2_7_LEFT | CCAATCCTGTCAATAACAATATCAGAAGAT |
| DENV2_7_RIGHT | TGATGGCTGGGGTTTGGTATCT |
| DENV2_8_LEFT | AGATCGAAGATGACATTTTCCGAAAGA |
| DENV2_8_RIGHT | CCCATGTATATGTACTGGTCATTTTCATT |
| DENV2_9_LEFT | ATGCCAGTGACCCACTCTAGTG |
| DENV2_9_RIGHT | CCACCACTGTGAGGATGGCTAT |
| DENV2_10_LEFT | ACCAGAAAAACAGAGAACACCCC |
| DENV2_10_RIGHT | CCACTTCCTGGATTCCACTTTTCT |
| DENV2_11_LEFT | GGAGCTGGACTTCTCTTTTCCAT |
| DENV2_11_RIGHT | GACGTCCCAAGGTTTTGTCAGC |
| DENV2_12_LEFT | AGAGCATGAAACATCATGGCACT |
| DENV2_12_RIGHT | GTGCCTCTTGGTGTTGGTCTTT |
| DENV2_13_LEFT | TGGGACACAAGAATCACACTAGAAG |
| DENV2_13_RIGHT | CCGCACCATTGGTCTTCTCTTT |

**^*^**Primers designed by the CADDE project (https://www.caddecentre.org/).

**Supplementary Table 6. Members of Latin American Genomic Surveillance Arboviral Network.**

| **Complete name** | **Affiliation** |
| --- | --- |
| Erenilde Marques de Cerqueira | Universidade Estadual de Feira de Santana, Salvador, Bahia, Brazil |
| Tiago Graf | Instituto Gonçalo Moniz, Fundação Oswaldo Cruz, Salvador, Bahia, Brazil |
| Walter Ramalho | Universidade de Brasília, Brasília, Distrito Federal, Brazil |
| Wildo Navegantes | Universidade de Brasília, Brasília, Distrito Federal, Brazil |
| Renato Barbosa Reis | Universidade Salvador, Salvador, Bahia, Brazil |
| Clara Guerra Duarte | Fundação Ezequiel Dias, Belo Horizonte, Minas Gerais, Brazil |
| Maira Alves Pereira | Fundação Ezequiel Dias, Belo Horizonte, Minas Gerais, Brazil |
| Paulo Eduardo de Souza da Silva | Fundação Ezequiel Dias, Belo Horizonte, Minas Gerais, Brazil |
| Raoni Almeida de Souza | Fundação Ezequiel Dias, Belo Horizonte, Minas Gerais, Brazil |
| Alex Pauvolid-Corrêa | Laboratório de Flavivírus, Instituto Oswaldo Cruz, Fundação Oswaldo Cruz, Rio de Janeiro, Rio de Janeiro, Brazil |
| Anne Aline Pereira de Paiva | Laboratório de Flavivírus, Instituto Oswaldo Cruz, Fundação Oswaldo Cruz, Rio de Janeiro, Rio de Janeiro, Brazil |
| Hegger Machado Fritsch | Laboratório de Flavivírus, Instituto Oswaldo Cruz, Fundação Oswaldo Cruz, Rio de Janeiro, Rio de Janeiro, Brazil |
| Maria Angélica Mares-Guia | Laboratório de Flavivírus, Instituto Oswaldo Cruz, Fundação Oswaldo Cruz, Rio de Janeiro, Rio de Janeiro, Brazil |
| Maria Celeste Torres | Laboratório de Flavivírus, Instituto Oswaldo Cruz, Fundação Oswaldo Cruz, Rio de Janeiro, Rio de Janeiro, Brazil |
| Maurício Teixeira Lima | Laboratório de Flavivírus, Instituto Oswaldo Cruz, Fundação Oswaldo Cruz, Rio de Janeiro, Rio de Janeiro, Brazil |
| Patrícia Sequeira | Laboratório de Flavivírus, Instituto Oswaldo Cruz, Fundação Oswaldo Cruz, Rio de Janeiro, Rio de Janeiro, Brazil |
| William de Almeida Marques | Laboratório de Flavivírus, Instituto Oswaldo Cruz, Fundação Oswaldo Cruz, Rio de Janeiro, Rio de Janeiro, Brazil |
| Jorlan Fernandes de Jesus | Laboratório de Hantaviroses e Rickettsioses, Instituto Oswaldo Cruz, Fundação Oswaldo Cruz, Rio de Janeiro, Rio de Janeiro, Brazil |
| Felipe Gomes Naveca | Laboratório de Ecologia de Doenças Transmissíveis na Amazônia, Instituto Leônidas e Maria Deane, Fundação Oswaldo Cruz, Manaus, Amazonas, Brazil |
| Alessandra Lima Silva | Instituto de Ciências Biológicas, Universidade Federal de Minas Gerais, Belo Horizonte, Minas Gerais, Brazil |
| Anne Cybelle Pinto | Instituto de Ciências Biológicas, Universidade Federal de Minas Gerais, Belo Horizonte, Minas Gerais, Brazil |
| Arun Kumar Jaiswal | Instituto de Ciências Biológicas, Universidade Federal de Minas Gerais, Belo Horizonte, Minas Gerais, Brazil |
| Élisson Nogueira Lopes | Instituto de Ciências Biológicas, Universidade Federal de Minas Gerais, Belo Horizonte, Minas Gerais, Brazil |
| Francielly Morais Rodrigues da Costa | Instituto de Ciências Biológicas, Universidade Federal de Minas Gerais, Belo Horizonte, Minas Gerais, Brazil |
| Gabriel Quintanilha-Peixoto | Instituto de Ciências Biológicas, Universidade Federal de Minas Gerais, Belo Horizonte, Minas Gerais, Brazil |
| Gilson Carlos Soares | Instituto de Ciências Biológicas, Universidade Federal de Minas Gerais, Belo Horizonte, Minas Gerais, Brazil |
| Paula Luize Camargos Fonseca | Instituto de Ciências Biológicas, Universidade Federal de Minas Gerais, Belo Horizonte, Minas Gerais, Brazil |
| Renan Pedra de Souza | Instituto de Ciências Biológicas, Universidade Federal de Minas Gerais, Belo Horizonte, Minas Gerais, Brazil |
| Rodrigo Bentes Kato | Instituto de Ciências Biológicas, Universidade Federal de Minas Gerais, Belo Horizonte, Minas Gerais, Brazil |
| Rodrigo Profeta Silveira Santos | Instituto de Ciências Biológicas, Universidade Federal de Minas Gerais, Belo Horizonte, Minas Gerais, Brazil |
| Sandeep Tiwari | Instituto de Ciências Biológicas, Universidade Federal de Minas Gerais, Belo Horizonte, Minas Gerais, Brazil |
| Wylerson Guimarães Nogueira | Instituto de Ciências Biológicas, Universidade Federal de Minas Gerais, Belo Horizonte, Minas Gerais, Brazil |
| Beatriz Senra Álvares da Silva Santos | Faculdade de Medicina Veterinária, Universidade Federal de Minas Gerais, Belo Horizonte, Minas Gerais, Brazil |
| Bruna Lopes Bueno | Faculdade de Medicina Veterinária, Universidade Federal de Minas Gerais, Belo Horizonte, Minas Gerais, Brazil |
| Isadora Cristina de Siqueira | Instituto Gonçalo Moniz, Fundação Oswaldo Cruz, Salvador, Bahia, Brazil |
| Lourdes Farre Vallve | Instituto Gonçalo Moniz, Fundação Oswaldo Cruz, Salvador, Bahia, Brazil |
| Melina Mosquera Navarro Borba | Instituto Gonçalo Moniz, Fundação Oswaldo Cruz, Salvador, Bahia, Brazil |
| Alix Sandra Mazzetto | Laboratório Central de Saúde Pública do Estado do Paraná, Curitiba, Paraná, Brazil |
| Francisco de Assis Araújo Aguiar | Laboratório Central de Saúde Pública do Estado de Rondônia, Porto Velho, Rondônia, Brazil |
| Irenio da Silva Gomes | Laboratório Central de Saúde Pública do Estado do Amazonas, Manaus, Amazonas, Brazil |
| Jayra Juliana Paiva Alves Abrantes | Laboratório Central de Saúde Pública do Estado do Rio Grande do Norte, Natal, Rio Grande do Norte, Brazil |
| Luiz Takao Watanabe | Laboratório Central de Saúde Pública do Estado de Mato Grosso, Cuiabá, Mato Grosso, Brazil |
| Marta Ferreira da Silva Rego | Laboratório Central de Saúde Pública Professor Gonçalo Muniz, Salvador, Bahia, Brazil |
| Vanessa Brandão Nardy | Laboratório Central de Saúde Pública Professor Gonçalo Muniz, Salvador, Bahia, Brazil |
| Shirlei Ferreira de Aguiar | Laboratório Central de Saúde Pública Noel Nutels, Rio de Janeiro, Rio de Janeiro, Brazil |
| Fabiana Cristina Pereira dos Santos | Instituto Adolfo Lutz, São Paulo, São Paulo, Brazil |
| Alice Louize Nunes Queiroz | Instituto Evandro Chagas, Belém, Pará, Brazil |
| Bruno Tardelli Diniz Nunes | Instituto Evandro Chagas, Belém, Pará, Brazil |
| Lívia Carício Martins | Instituto Evandro Chagas, Belém, Pará, Brazil |
| Márcio Roberto Teixeira Nunes | Instituto Evandro Chagas, Belém, Pará, Brazil |
| Flávia Cristina da Silva Salles | Instituto de Medicina Tropical, Universidade de São Paulo, São Paulo, São Paulo, Brazil |
| Ingra Morales Claro | Instituto de Medicina Tropical, Universidade de São Paulo, São Paulo, São Paulo, Brazil |
| Jaqueline Goes de Jesus | Instituto de Medicina Tropical, Universidade de São Paulo, São Paulo, São Paulo, Brazil |
| Darlan da Silva Cândido | Department of Zoology, Peter Medawar Building, University of Oxford, Oxford, United Kingdom |
| Cintia Marcela Fabbri | Instituto Nacional de Enfermedades Virales Humanas Dr. Julio Maiztegui, Pergamino, Argentina |
| Claudia González | Gorgas Memorial Institute for Health Studies, Panama, Panama |
| Lisseth Saéz | Gorgas Memorial Institute for Health Studies, Panama, Panama |
| María Chen-Germán | Gorgas Memorial Institute for Health Studies, Panama, Panama |
| Jaime Lagos Barrera | Instituto de Salud Pública de Chile, Santiago, Chile |
| José Ernesto Ramírez-González | Instituto de Diagnóstico y Referencia Epidemiológicos Dr. Manuel Martínez Báez, Ciudad de México, México |
| Josefina Campos | Instituto Nacional de Enfermedades Infecciosas Dr Carlos G Malbrán, Buenos Aires, Argentina |
| Noelia Morel Faller | Ministerio de Salud Pública de Uruguay, Montevideo, Uruguay |
| Marta Eugenia Víquez Villalobos | Instituto Costarricense de Investigación y Enseñanza em Nutrición y Salud, Tres Ríos, Costa Rica |
| Roberto Kaslin | Instituto Nacional de Investigacion en Salud Publica Dr Leopoldo Izquieta Pérez, Guayaquil, Ecuador |
| Silvia Paola Salgado Cisneros | Instituto Nacional de Investigacion en Salud Publica Dr Leopoldo Izquieta Pérez, Guayaquil, Ecuador |
| Flávia Figueira Aburjaile | Universidade Federal de Pernambuco, Recife, Pernambuco, Brazil |
| Carolina Dourado Amaral | Secretaria de Saúde do Estado de Minas Gerais, Belo Horizonte, Minas Gerais, Brazil |
| Danielle Bandeira Costa de Sousa Freire | Secretaria de Vigilância em Saúde/Ministério da Saúde, Brasília, Distrito Federal, Brazil |
| Laura Nogueira Cruz | Secretaria de Vigilância em Saúde/Ministério da Saúde, Brasília, Distrito Federal, Brazil |
| Daniel Mattos | Universidade Federal do Rio de Janeiro, Rio de Janeiro, Rio de Janeiro, Brazil |
| Leandro Ferreira Lopes Landeira | Universidade Federal do Rio de Janeiro, Rio de Janeiro, Rio de Janeiro, Brazil |
| Mariane Talon de Menezes | Universidade Federal do Rio de Janeiro, Rio de Janeiro, Rio de Janeiro, Brazil |
| Ieda Maria Orioli | Universidade Federal do Rio de Janeiro, Rio de Janeiro, Rio de Janeiro, Brazil |
| Ariane Coelho Ferraz | Universidade Federal de Ouro Preto, Ouro Preto, Minas Gerais, Brazil |
| Daiane Teixeira de Oliveira | Universidade Federal de Ouro Preto, Ouro Preto, Minas Gerais, Brazil |
| Alexandre Barbosa Reis | Universidade Federal de Ouro Preto, Ouro Preto, Minas Gerais, Brazil |
| Renata Guerra de Sá Cota | Universidade Federal de Ouro Preto, Ouro Preto, Minas Gerais, Brazil |
| Rafael dos Santos Bezerra | Fundação Hemocentro de Ribeirão Preto, Ribeirão Preto, São Paulo, Brazil |
| Melissa Barreto Falcão | Secretaria de Saúde de Feira de Santana, Feira de Santana, Bahia, Brazil |
| Rodrigo Dias de Oliveira Carvalho | Sechenov First Moscow State Medical University, Moscou, Russia |

**Supplementary Text File 1**

**Course *“Nanopore-based genome sequencing technology for temporal investigation and epidemiology of dengue outbreak: training, research, surveillance, and scientific dissemination”***

Recent progress in the sequencing of Ebola, Zika, and yellow fever viruses using Oxford Nanopore sequencing technology have shown that high quality complete genome sequences can be generated in real time during viral outbreaks and epidemics.^1–3^ Portability and rapid production of data allows generation of genomes at the source of outbreaks, which in turn facilitate rapid intervention. Considering this and the strengthening of surveillance actions in Latin America, the Pan American Health Organization/World Health Organization (PAHO/WHO), together with the Brazilian Ministry of Health (BrMoH), planned the course entitled “Nanopore-based genome sequencing technology for temporal investigation and epidemiology of dengue outbreak: training, research, surveillance, and scientific dissemination”, under the coordination of Prof. Dr. Luiz Alcantara, researcher at Fundação Oswaldo Cruz (https://portal.fiocruz.br/).

The course took place in the city of Belo Horizonte, Minas Gerais state, from August 19 to 30, 2019 and it aimed at carrying out activities central to arbovirus genomic surveillance, focusing on the training of participants, dissemination of knowledge, and scientific communication. The course was taught by experienced researchers from national and international institutions, such as Oxford University (United Kingdom), University of KwaZulu-Natal (South Africa), Universidade Nova de Lisboa (Portugal), Sechenov First Moscow State Medical University (Russia), Oswaldo Cruz Foundation (Brazil), Federal University of Minas Gerais (Brazil), Federal University of Rio de Janeiro (Brazil), Federal University of Pernambuco (Brazil), University of São Paulo (Brazil), University of Brasilia (Brazil), State University of Feira de Santana (Brazil), and University of Salvador (Brazil). The course had 62 students from 34 national and international institutions. In addition to post-graduate students, course participants included laboratory technicians and health practitioners in universities and laboratories from several institutions responsible for laboratory-based surveillance of emerging and reemerging diseases, such as the Central Public Health Laboratories of the Brazilian states from the BrMoH's public laboratories network and public health laboratories from Paraguay, Argentina, Panama, Chile, Mexico, Uruguay, Costa Rica, and Ecuador.

The course lasted two weeks and comprised theoretical and practical classes focused on genomic surveillance of dengue (DENV1 and DENV2 serotypes), which was responsible for a large epidemic in 2019, accounting for 1,544,987 cases reported in Brazil.^4^ The first week of training addressed the genomic sequencing protocol using Nanopore technology and analysis of clinical and epidemiological data from selected samples collected in three Brazilian macro-regions (Midwest, Southeast, and Northeast), which historically has reported the largest incidences of dengue.^5^ In that stage, participants learned how to prepare DNA library and sequencing using the MinION portable sequencer (Oxford Nanopore Technologies). They were also trained on the generation of consensus sequences and genotyping using the Genome Detective tool (https: //www.genomedetective.com/), on basic topics of epidemiological modeling, and epidemiological maps using R and QGIS software. The second week of the course focused on evolutionary analysis using the genome sequences of DENV1 and DENV2 generated in the previous week. Participants were trained in genomic data preparation, construction of genome dataset from public databases (such as Genbank and ViPR), genome alignment, alignment editing, phylogenetic reconstructions by Maximum Likelihood (ML) approach, in addition to temporal and phylogeographic inference using Bayesian approaches. Participants carried out preliminary epidemiological and evolutionary analyzes for the preparation of this manuscript.

**Date:** August 19-30, 2019

**City:** Belo Horizonte, Minas Gerais state, Brazil

**Course hours:** 96 hours (40h of theoretical classes and 56h of practical classes)

**FUNDING**

Pan American World Health Organization (PAHO/WHO) and Secretaria de Vigilância em Saúde (SVS)/Brazilian Ministry of Health.

**ORGANIZERS**

- Luiz Carlos Junior Alcantara, Laboratório de Flavivírus, Instituto Oswaldo Cruz, Fundação Oswaldo Cruz, Rio de Janeiro, Rio de Janeiro Brazil;
- Carlos Frederico Campelo de Albuquerque, Organização Pan-Americana da Saúde/Organização Mundial da Saúde, Brasília, Distrito Federal, Brazil;
- André Luiz de Abreu, Coordenação Geral dos Laboratórios de Saúde Pública, Secretaria de Vigilância em Saúde, Ministério da Saúde, Brasília, Distrito Federal, Brazil;
- Rodrigo Fabiano do Carmo Said, Coordenação Geral das Arboviroses, Secretaria de Vigilância em Saúde/Ministério da Saúde, Brasília, Distrito Federal, Brazil;
- Marluce Aparecida Assunção Oliveira, Laboratório Central de Saúde Pública do Estado de Minas Gerais, Fundação Ezequiel Dias, Belo Horizonte, Minas Gerais, Brazil;
- Mauricio Abreu Santos, Laboratório Central de Saúde Pública do Estado de Minas Gerais, Fundação Ezequiel Dias, Belo Horizonte, Minas Gerais, Brazil;
- Maricélia Maia de Lima, Secretaria de Saúde de Feira de Santana, Feira de Santana, Bahia, Brazil;
- Gabriel Muricy Cunha, Secretaria de Saúde do Estado da Bahia, Salvador, Bahia, Brazil;
- Vinicius Lemes da Silva, Laboratório Central de Saúde Pública Dr. Giovanni Cysneiros, Goiânia, Goiás, Brazil;
- Ana Maria Bispo de Fellipis, Laboratório de Flavivírus, Instituto Oswaldo Cruz, Fundação Oswaldo Cruz, Rio de Janeiro, Rio de Janeiro, Brazil;
- Vasco Azevedo, Instituto de Ciências Biológicas, Universidade Federal de Minas Gerais, Belo Horizonte, Minas Gerais, Brazil;
- Marta Giovanetti, Laboratório de Flavivírus, Instituto Oswaldo Cruz, Fundação Oswaldo Cruz, Rio de Janeiro, Rio de Janeiro, Brazil.

**LECTURERS**

**International**

- José Lourenço, Department of Zoology, Peter Medawar Building, University of Oxford, Oxford, United Kingdom;
- Tulio de Oliveira, KwaZulu-Natal Research Innovation and Sequencing Platform (KRISP), College of Health Sciences, University of KwaZulu-Natal, Durban, South Africa.

**Brazilian**

- Luiz Carlos Junior Alcantara, Laboratório de Flavivírus, Instituto Oswaldo Cruz, Fundação Oswaldo Cruz, Rio de Janeiro, Rio de Janeiro, Brazil;
- Rivaldo Venâncio, Fundação Oswaldo Cruz, Bio-Manguinhos, Rio de Janeiro, Rio de Janeiro, Brazil;
- Ana Maria Bispo de Fellipis, Laboratório de Flavivírus, Instituto Oswaldo Cruz, Fundação Oswaldo Cruz, Rio de Janeiro, Rio de Janeiro, Brazil;
- Rita Maria Ribeiro Nogueira, Laboratório de Flavivírus, Instituto Oswaldo Cruz, Fundação Oswaldo Cruz, Rio de Janeiro, Rio de Janeiro, Brazil;
- Erenilde Marques de Cerqueira, Universidade Estadual de Feira de Santana, Salvador, Bahia, Brazil;
- Wildo Navegantes de Araújo, Universidade de Brasília, Brasília, Distrito Federal, Brazil;
- Melissa Barreto Falcão, Secretaria de Saúde de Feira de Santana, Feira de Santana, Bahia, Brazil;
- Renato Barbosa Reis, Universidade Salvador, Salvador, Bahia, Brazil;
- Tiago Graf, Instituto Gonçalo Moniz, Fundação Oswaldo Cruz, Salvador, Bahia, Brazil;
- Marta Giovanetti, Laboratório de Flavivírus, Instituto Oswaldo Cruz, Fundação Oswaldo Cruz, Rio de Janeiro, Rio de Janeiro, Brazil;
- Ingra Morales Claro, Instituto de Medicina Tropical, Universidade de São Paulo, São Paulo, São Paulo, Brazil;
- Jaqueline de Jesus, Instituto de Medicina Tropical, Universidade de São Paulo, São Paulo, São Paulo, Brazil;
- Isadora Cristina de Siqueira, Instituto Gonçalo Moniz, Fundação Oswaldo Cruz, Salvador, Bahia, Brazil;
- Joilson Xavier, Laboratório de Flavivírus, Instituto Oswaldo Cruz, Fundação Oswaldo Cruz, Rio de Janeiro, Rio de Janeiro, Brazil;
- Mariane Talon Menezes, Universidade Federal do Rio de Janeiro, Rio de Janeiro, Rio de Janeiro, Brazil;
- Flavia Cristina da Silva Sales, Instituto de Medicina Tropical, Universidade de São Paulo, São Paulo, São Paulo, Brazil;
- Fernanda de Bruycker Nogueira, Laboratório de Flavivírus, Instituto Oswaldo Cruz, Fundação Oswaldo Cruz, Rio de Janeiro, Rio de Janeiro, Brazil;
- Felipe Campos de Melo Iani, Laboratório Central de Saúde Pública do Estado de Minas Gerais, Fundação Ezequiel Dias, Belo Horizonte, Minas Gerias, Brazil;
- Talita Émile Ribeiro Adelino, Laboratório Central de Saúde Pública do Estado de Minas Gerais, Fundação Ezequiel Dias, Belo Horizonte, Minas Gerais, Brazil;
- Valdinete Alves do Nascimento, Laboratório de Ecologia de Doenças Transmissíveis na Amazônia, Instituto Leônidas e Maria Deane, Fiocruz, Manaus, Amazonas, Brazil;
- Allison de Araújo Fabri, Laboratório de Flavivírus, Instituto Oswaldo Cruz, Fundação Oswaldo Cruz, Rio de Janeiro, Rio de Janeiro, Brazil;
- Flávia Löwen Levy Chalhoub, Laboratório de Flavivírus, Instituto Oswaldo Cruz, Fundação Oswaldo Cruz, Rio de Janeiro, Rio de Janeiro, Brazil;
- Álvaro Salgado de Abreu, Laboratório de Flavivírus, Instituto Oswaldo Cruz, Fundação Oswaldo Cruz, Rio de Janeiro, Rio de Janeiro, Brazil;
- Vagner Fonseca, Laboratório de Flavivírus, Instituto Oswaldo Cruz, Fundação Oswaldo Cruz, Rio de Janeiro, Rio de Janeiro, Brazil;
- Francielly Rodrigues da Costa, Instituto de Ciências Biológicas, Universidade Federal de Minas Gerais, Belo Horizonte, Minas Gerais, Brazil;
- Jorlan Fernandes de Jesus, Laboratório de Hantaviroses e Rickettsioses, Instituto Oswaldo Cruz, Fundação Oswaldo Cruz, Rio de Janeiro, Rio de Janeiro, Brazil;
- Maricélia Maia de Lima, Secretaria de Saúde de Feira de Santana, Feira de Santana, Bahia, Brazil;
- Victor Pimentel, Instituto de Higiene e Medicina Tropical, Universidade Nova de Lisboa, Lisboa, Portugal;
- Darlan da Silva Cândido, Department of Zoology, Peter Medawar Building, University of Oxford, Oxford, United Kingdom;
- Flávia Figueira Aburjaile, Universidade Federal de Pernambuco, Recife, Pernambuco, Brazil;
- Rodrigo Días de Oliveira Carvalho, Sechenov First Moscow State Medical University, Moscou, Russia.

**STUDENTS**

| **Name** | **Affiliation** | **Country** |
| --- | --- | --- |
| Clara Guerra Duarte | Fundação Ezequiel Dias, Belo Horizonte, Minas Gerais, Brazil | Brazil |
| Maira Alves Pereira | Fundação Ezequiel Dias, Belo Horizonte, Minas Gerais, Brazil | Brazil |
| Paulo Eduardo de Souza da Silva | Fundação Ezequiel Dias, Belo Horizonte, Minas Gerais, Brazil | Brazil |
| Raoni Almeida de Souza | Fundação Ezequiel Dias, Belo Horizonte, Minas Gerais, Brazil | Brazil |
| Anne Aline Pereira de Paiva | Laboratório de Flavivírus, Instituto Oswaldo Cruz, Fundação Oswaldo Cruz, Rio de Janeiro, Rio de Janeiro, Brazil | Brazil |
| Hegger Machado Fritsch | Laboratório de Flavivírus, Instituto Oswaldo Cruz, Fundação Oswaldo Cruz, Rio de Janeiro, Rio de Janeiro, Brazil | Brazil |
| Maria Angélica Mares-Guia | Laboratório de Flavivírus, Instituto Oswaldo Cruz, Fundação Oswaldo Cruz, Rio de Janeiro, Rio de Janeiro, Brazil | Brazil |
| Maurício Teixeira Lima | Laboratório de Flavivírus, Instituto Oswaldo Cruz, Fundação Oswaldo Cruz, Rio de Janeiro, Rio de Janeiro, Brazil | Brazil |
| Stephane Fraga de Oliveira Tosta | Laboratório de Flavivírus, Instituto Oswaldo Cruz, Fundação Oswaldo Cruz, Rio de Janeiro, Rio de Janeiro, Brazil | Brazil |
| Ronaldo de Jesus | Coordenação Geral dos Laboratórios de Saúde Pública/Secretaria de Vigilância em Saúde, Ministério da Saúde, Brasília, Distrito Federal, Brazil | Brazil |
| Emerson Luiz Lima de Araújo | Coordenação Geral dos Laboratórios de Saúde Pública/Secretaria de Vigilância em Saúde, Ministério da Saúde, Brasília, Distrito Federal, Brazil | Brazil |
| Carlos Frederico Campelo de Albuquerque | Organização Pan-Americana da Saúde/Organização Mundial da Saúde, Brasília, Distrito Federal, Brazil | Brazil |
| Alessandra Lima Silva | Instituto de Ciências Biológicas, Universidade Federal de Minas Gerais, Belo Horizonte, Minas Gerais, Brazil | Brazil |
| Anne Cybelle Pinto | Instituto de Ciências Biológicas, Universidade Federal de Minas Gerais, Belo Horizonte, Minas Gerais, Brazil | Brazil |
| Arun Kumar Jaiswal | Instituto de Ciências Biológicas, Universidade Federal de Minas Gerais, Belo Horizonte, Minas Gerais, Brazil | Brazil |
| Élisson Nogueira Lopes | Instituto de Ciências Biológicas, Universidade Federal de Minas Gerais, Belo Horizonte, Minas Gerais, Brazil | Brazil |
| Gabriel Quintanilha-Peixoto | Instituto de Ciências Biológicas, Universidade Federal de Minas Gerais, Belo Horizonte, Minas Gerais, Brazil | Brazil |
| Paula Luize Camargos Fonseca | Instituto de Ciências Biológicas, Universidade Federal de Minas Gerais, Belo Horizonte, Minas Gerais, Brazil | Brazil |
| Renan Pedra de Souza | Instituto de Ciências Biológicas, Universidade Federal de Minas Gerais, Belo Horizonte, Minas Gerais, Brazil | Brazil |
| Sandeep Tiwari | Instituto de Ciências Biológicas, Universidade Federal de Minas Gerais, Belo Horizonte, Minas Gerais, Brazil | Brazil |
| Wylerson Guimarães Nogueira | Instituto de Ciências Biológicas, Universidade Federal de Minas Gerais, Belo Horizonte, Minas Gerais, Brazil | Brazil |
| Beatriz Senra Álvares da Silva Santos | Faculdade de Medicina Veterinária, Universidade Federal de Minas Gerais, Belo Horizonte, Minas Gerais, Brazil | Brazil |
| Bruna Lopes Bueno | Faculdade de Medicina Veterinária, Universidade Federal de Minas Gerais, Belo Horizonte, Minas Gerais, Brazil | Brazil |
| Melina Mosquera Navarro Borba | Instituto Gonçalo Moniz, Fundação Oswaldo Cruz, Salvador, Bahia, Brazil | Brazil |
| Agenor de Castro Moreira dos Santos Júnior | Laboratório Central de Saúde Pública do Distrito Federal, Brasília, Distrito Federal, Brazil | Brazil |
| Alix Sandra Mazzetto | Laboratório Central de Saúde Pública do Estado do Paraná, Curitiba, Paraná, Brazil | Brazil |
| Francisco de Assis Araújo Aguiar | Laboratório Central de Saúde Pública do Estado de Rondônia, Porto Velho, Rondônia, Brazil | Brazil |
| Gislene Garcia de Castro Lichs | Laboratório Central de Saúde Pública do Estado de Mato Grosso do Sul, Campo Grande, Mato Grosso do Sul, Brazil | Brazil |
| Marina Castilhos Souza Umaki Zardin | Laboratório Central de Saúde Pública do Estado de Mato Grosso do Sul, Campo Grande, Mato Grosso do Sul, Brazil | Brazil |
| Irenio da Silva Gomes | Laboratório Central de Saúde Pública do Estado do Amazonas, Manaus, Amazonas, Brazil | Brazil |
| Jayra Juliana Paiva Alves Abrantes | Laboratório Central de Saúde Pública do Estado do Rio Grande do Norte, Natal, Rio Grande do Norte, Brazil | Brazil |
| Jurandy Júnior Ferraz de Magalhães | Laboratório Central de Saúde Pública Dr. Milton Bezerra Sobral, Recife, Pernambuco, Brazil | Brazil |
| Ana Flávia Mendonça | Laboratório Central de Saúde Pública Dr. Giovanni Cysneiros, Goiânia, Goiás, Brazil | Brazil |
| Luiz Augusto Pereira | Laboratório Central de Saúde Pública Dr. Giovanni Cysneiros, Goiânia, Goiás, Brazil | Brazil |
| Luiz Takao Watanabe | Laboratório Central de Saúde Pública do Estado de Mato Grosso, Cuiabá, Mato Grosso, Brazil | Brazil |
| Marta Ferreira da Silva Rego | Laboratório Central de Saúde Pública Professor Gonçalo Muniz, Salvador, Bahia, Brazil | Brazil |
| Vanessa Brandão Nardy | Laboratório Central de Saúde Pública Professor Gonçalo Muniz, Salvador, Bahia, Brazil | Brazil |
| Shirlei Ferreira de Aguiar | Laboratório Central de Saúde Pública Noel Nutels, Rio de Janeiro, Rio de Janeiro, Brazil | Brazil |
| Fabiana Cristina Pereira dos Santos | Instituto Adolfo Lutz, São Paulo, São Paulo, Brazil | Brazil |
| Alice Louize Nunes Queiroz | Instituto Evandro Chagas, Ananindeua, Pará, Brazil | Brazil |
| Bruno Tardelli Diniz Nunes | Instituto Evandro Chagas, Ananindeua, Pará, Brazil | Brazil |
| Cynthia Carolina Vazquez | Laboratorio Central de Salud Pública, Asunción, Paraguay | Paraguay |
| Cintia Marcela Fabbri | Instituto Nacional de Enfermedades Virales Humanas Dr. Julio Maiztegui, Pergamino, Argentina | Argentina |
| Alexander Martinez | Gorgas Memorial Institute for Health Studies, Panama, Panama | Panama |
| Claudia González | Gorgas Memorial Institute for Health Studies, Panama, Panama | Panama |
| Lisseth Saéz | Gorgas Memorial Institute for Health Studies, Panama, Panama | Panama |
| María Chen-Germán | Gorgas Memorial Institute for Health Studies, Panama, Panama | Panama |
| Jaime Lagos Barrera | Instituto de Salud Pública de Chile, Santiago, Chile | Chule |
| José Ernesto Ramírez-González | Instituto de Diagnóstico y Referencia Epidemiológicos Dr. Manuel Martínez Báez, Ciudad de México, Mexico | Mexico |
| Josefina Campos | Instituto Nacional de Enfermedades Infecciosas Dr Carlos G Malbrán, Buenos Aires, Argentina | Argentina |
| Noelia Morel Faller | Ministerio de Salud Pública de Uruguay, Montevideo, Uruguay | Uruguay |
| Marta Eugenia Víquez Villalobos | Instituto Costarricense de Investigación y Enseñanza em Nutrición y Salud, Tres Ríos, Costa Rica | Costa Rica |
| Roberto Kaslin | Instituto Nacional de Investigacion en Salud Publica Dr Leopoldo Izquieta Pérez, Guayaquil, Ecuador | Ecuador |
| Silvia Paola Salgado Cisneros | Instituto Nacional de Investigacion en Salud Publica Dr Leopoldo Izquieta Pérez, Guayaquil, Ecuador | Ecuador |
| Carolina Dourado Amaral | Secretaria de Saúde do Estado de Minas Gerais, Belo Horizonte, Minas Gerais, Brazil | Brazil |
| Danielle Bandeira Costa de Sousa Freire | Secretaria de Vigilância em Saúde/Ministério da Saúde, Brasília, Distrito Federal, Brazil | Brazil |
| Laura Nogueira Cruz | Secretaria de Vigilância em Saúde/Ministério da Saúde, Brasília, Distrito Federal, Brazil | Brazil |
| Daniel Mattos | Universidade Federal do Rio de Janeiro, Rio de Janeiro, Rio de Janeiro, Brazil | Brazil |
| Leandro Ferreira Lopes Landeira | Universidade Federal do Rio de Janeiro, Rio de Janeiro, Rio de Janeiro, Brazil | Brazil |
| Ariane Coelho Ferraz | Universidade Federal de Ouro Preto, Ouro Preto, Minas Gerais, Brazil | Brazil |
| Daiane Teixeira de Oliveira | Universidade Federal de Ouro Preto, Ouro Preto, Minas Gerais, Brazil | Brazil |
| Rafael dos Santos Bezerra | Fundação Hemocentro de Ribeirão Preto, Ribeirão Preto, São Paulo, Brazil | Brazil |

**COURSE SCHEDULE**

**Day 1 – Monday – August 19, 2019 – Wet laboratories from Fundação Ezequiel Dias (FUNED)**

**(Coordinator: Ingra Morales)**

**08:00-09:00:** Introduction to Nanopore-based sequencing technology. From sample handling to sequencing PCR. Lecturers: Ingra Morales e Jaqueline de Jesus (theory). Participants splitting into 3 classes of 12 students/class (A, B e C).

**09:00-12:00:** Library preparation:

**Group A:** DENV Library preparation from selected samples for group/class A. Lecturers: Jaqueline de Jesus, Flávia Sales, Valdinete Nascimento, and Flávia Chalhoub. (Theory and practice)

**Group B:** DENV Library preparation from selected samples for group/class B. Lecturers: Ingra Morales, Talita Adelino, Fernanda Nogueira, and Allison Fabri. (Theory and practice)

**Group C:** DENV Library preparation from selected samples for group/class C**.** Lecturers: Joilson Xavier, Mariane Menezes, Darlan Candido, and Felipe Iani. (Theory and practice)

**12:00-13:00:** Lunch time

**13:00-16:00:** DNA library preparation and sequencing on MinION. Wet lab at FUNED:

**Group A:** DNA library preparation and sequencing on MinION of samples from Group A. Lecturers: Jaqueline de Jesus, Flávia Sales, Valdinete Nascimento, and Flávia Chalhoub. (Theory and practice)

**Group B:** DNA library preparation and sequencing on MinION of samples from Group B. Lecturers: Ingra Morales, Talita Adelino, Fernanda Nogueira, and Allison Fabri. (Theory and practice)

**Turma C:** DNA library preparation and sequencing on MinION of samples from Group C**.** Lecturers: Joilson Xavier, Mariane Menezes, Darlan Candido, and Felipe Iani. (Theory and practice)

**16:00-16:30:** Coffee Break

**16:30-17:00:** **Amphitheater at FUNED:** Course presentation (Prof. Luiz Alcantara) and guests (Andre Abreu, Julio Croda, Wanderson Oliveira, Rodrigo Said, Carlos Eduardo Amaral Pereira da Silva, Dario Brock Ramalho, Maurício Santos, Marluce Oliveira, Gabriel Muricy, and Vinicius Silva)

**17:00-17:30:** **Amphitheater at FUNED:** Presentation of the coordination of the dengue surveillance group/DEVIT/SVS-MS and PAHO/WHO in Brazil. "Panorama and surveillance of dengue in the current outbreak in Brazil". Lecturer: Rodrigo Said

**17:30-18:00:** **Amphitheater at FUNED:** Historical overview of the dengue epidemic in Brazil. Lecturers: Rita Nogueira (speaker) and Ana de Filippis (moderator)

**18:00-18:30:** **Amphitheater at FUNED:** Genomic surveillance of arboviruses in Brazil and Paraguay. Lecturer: Luiz Alcantara.

**Day 2 – Tuesday - August 20, 2019 – Computer room**

**(Coordinator: Wildo Navegantes)**

**08:00-12:00:** Basic concepts of “R” applied to studies of epidemiological modeling. Lecturers: Álvaro Salgado, Vagner Fonseca, Francielly Rodrigues, and Rodrigo Kato. (Theory)

**12:00-13:30:** Lunch time.

**13:30-15:10:** Basic concepts of “R” applied to studies of epidemiological modeling. Lecturers: Álvaro Salgado, Vagner Fonseca, Francielly Rodrigues, and Rodrigo Kato. (Theory and practice)

**15:10-15:30:** Coffee Break

**15:30-17:00:** Basic concepts of “R” applied to studies of epidemiological modeling. Lecturers: Álvaro Salgado, Vagner Fonseca, Francielly Rodrigues, and Rodrigo Kato. (Practice)

**Day 3 – Wednesday - August 21, 2019 – Computer room**

**(Coordinator: Renato Reis)**

**08:00-12:00:** Basic concepts of “R” applied to studies of epidemiological modeling. Lecturers: Álvaro Salgado, Vagner Fonseca, Francielly Rodrigues, and Rodrigo Kato. (Theory)

**12:00-13:30:** Lunch time.

**13:30-15:10:** Basic concepts of “R” applied to studies of epidemiological modeling. Lecturers: Álvaro Salgado, Vagner Fonseca, Francielly Rodrigues, and Rodrigo Kato. (Theory and practice)

**15:10-15:30:** Coffee Break

**15:30-17:00:** Basic concepts of “R” applied to studies of epidemiological modeling. Lecturers: Álvaro Salgado, Vagner Fonseca, Francielly Rodrigues, and Rodrigo Kato. (Practice)

**Day 4 – Thursday - August 22, 2019 – Computer room**

**(Coordinator: José Lourenço)**

**08:00-12:00:** Epidemiological modeling to predict outbreaks of arboviral diseases and estimation of epidemiological parameters of outbreaks. Lecturers: José Lourenço. (Theory)

**12:00-13:30:** Lunch time.

**13:30-15:10:** Epidemiological modeling to predict outbreaks of arboviral diseases and estimation of epidemiological parameters of outbreaks. Lecturers: José Lourenço, Erenilde Cerqueira, and Wildo Navegantes. (Theory and practice)

**15:10-15:30:** Coffee Break

**15:30-17:00:** Epidemiological modeling to predict outbreaks of arboviral diseases and estimation of epidemiological parameters of outbreaks. Lecturers: José Lourenço, Erenilde Cerqueira, and Wildo Navegantes. (Practice)

**Day 5 – Friday - August 23, 2019 – Computer room**

**(Coordinators: Wildo Navegantes and Renato Reis**)

**08:00-12:00:** Implementation of epidemiological maps from raw data, using "R" and "QGIS", and calculation of spatial and temporal determinants in arboviral infections. Lecturers: Renato Reis and Wildo Navegantes. (Theory)

**12:00-13:30:** Lunch time.

**13:30-15:10:** Implementation of epidemiological maps from raw data, using "R" and "QGIS", and calculation of spatial and temporal determinants in arboviral infections. Lecturers: Renato Reis, Wildo Navegantes, Isadora de Siqueira, and Maricélia Lima. (Theory and practice)

**15:10-15:30:** Coffee Break

**15:30-17:00:** Implementation of epidemiological maps from raw data, using "R" and "QGIS", and calculation of spatial and temporal determinants in arboviral infections. Lecturers: Wildo Navegantes, Renato Reis, Isadora de Siqueira, and Maricélia Lima. (Practice)

**Days 6 and 7 – Saturday and Sunday – August 24-25, 2019 – Computer room**

**(Coordinator: Luiz Alcantara)**

Generation of consensus sequences from MinION raw data. Lecturers**:** Vagner Fonseca, Álvaro Salgado, Marta Giovanetti, Ingra Morales, Flávia Aburjaile, and Rodrigo Dias de Oliveira Carvalho. (Practice)

**Day 8 – Monday – August 26, 2019 – Computer room**

**08:00-12:00:** Organization and alignment of genomes to reference sequences, and phylogenetic reconstruction: NJ and ML. Lecturers: Marta Giovanetti, Fernanda Nogueira, Luiz Alcantara, Flávia Aburjaile, and Rodrigo Dias de Oliveira Carvalho. (Theory and practice)

**12:00-13:30:** Lunch time.

**13:30-15:10:** Temporal evolutionary analyzes. Lecturers: Tiago Graf, Marta Giovanetti, Darlan Candido, and Jorlan de Jesus. (Theory and practice)

**15:10-15:30:** Coffee Break

**15:30-16:30:** Temporal evolutionary analyzes. Lecturers: Tiago Graf, Marta Giovanetti, Victor Pimentel, Jorlan de Jesus, and Flávia Aburjaile. (Practice)

**16:30-17:30:** “Genome Detective”, Viral Metagenomics and Online Viral Genotyping Tools. Lecturers: Vagner Fonseca, Ingra Morales, Marta Giovanetti, Flávia Aburjaile, and Rodrigo Dias de Oliveira Carvalho. (Theory)

**Day 9 – Tuesday – August 27, 2019 – Computer room**

**08:00-12:00:** Phylogeographic analysis. Lecturers: Tiago Graf, Marta Giovanetti, and Fernanda Nogueira. (Theory and practice)

**12:00-13:30:** Lunch time.

**13:30-15:10:** Phylogeographic analysis. Lecturers: Tiago Graf, Marta Giovanetti, and Fernanda Nogueira. (Theory and practice)

**15:10-15:30:** Coffee Break

**15:30-16:10:** “Genome Detective”, Viral Metagenomics and Online Viral Genotyping Tools. Lecturers: Vagner Fonseca, Ingra Morales, Marta Giovanetti, Flávia Aburjaile, and Rodrigo Dias de Oliveira Carvalho. (Theory)

**16:10-18:00:** Phylogeographic analysis. Lecturers: Tiago Graf, Marta Giovanetti, Darlan Cândido, and Fernanda Nogueira. (Practice)

**Day 10 – Wednesday – August 28, 2019 – Amphitheater**

**08:00-12:00:** Preliminary epidemiological and evolutionary analyzes from the data generated during the course.

**12:00-13:30:** Lunch time.

**13:30-15:10:** Preliminary epidemiological and evolutionary analyzes from the data generated during the course.

**15:10-15:30:** Coffee Break

**15:30-17:00:** Preliminary epidemiological and evolutionary analyzes from the data generated during the course.

**17:00-18:00:** Temporal clinical surveillance of dengue in Bahia and Brazil. Lecturers: Melissa Falcão and Rivaldo Venâncio.

**Day 11 – Thursday – August 29, 2019 – Amphitheater**

**08:00-10:00:** Surveillance of arboviral outbreaks through epidemiological and evolutionary temporal studies: putting the results in the format for a publication. Lecturers: Tulio de Oliveira, Tiago Graf, and Marta Giovanetti. (Theory and practice)

**10:00-12:00:** Surveillance of arboviral outbreaks through epidemiological and evolutionary temporal studies: putting the results in the format for a publication. Lecturers: Tulio de Oliveira, Tiago Graf, and Marta Giovanetti. (Theory and practice)

**12:00-13:30:** Lunch time.

**13:30-15:10:** Preliminary epidemiological and evolutionary analyzes from the data generated during the course.

**15:10-15:30:** Coffee Break

**15:30-18:00:** Preliminary epidemiological and evolutionary analyzes from the data generated during the course.

**Day 12 – Friday – August 30, 2019 – Amphitheater**

**08:00-12:00:** Preliminary epidemiological and evolutionary analyzes from the data generated during the course.

**12:00-13:30:** Lunch time.

**13:30-15:10:** Preliminary epidemiological and evolutionary analyzes from the data generated during the course.

**15:10-15:30:** Coffee Break

**15:30-18:00:** Preliminary epidemiological and evolutionary analyzes from the data generated during the course.

**REFERENCES**

1 Quick, J. *et al*. Real-time, portable genome sequencing for Ebola surveillance. *Nature* **530,** 228–232 (2016).

2 Quick, J. *et al*. Multiplex PCR method for MinION and Illumina sequencing of Zika and other virus genomes directly from clinical samples. *Nat. Protoc.* **12,** 1261–1276 (2017).

3 Faria, N.R. *et al*. Genomic and epidemiological monitoring of yellow fever virus transmission potential. *Science* **361,** 894–899 (2018).

4 Brasil. Ministério da Saúde. Monitoramento dos casos de arboviroses urbanas transmitidas pelo *Aedes* (dengue, chikungunya e Zika), Semanas Epidemiológicas 01 a 52. 2020. <https://www.saude.gov.br/images/pdf/2020/janeiro/20/Boletim-epidemiologico-SVS-02-1-.pdf> (accessed May 20, 2020).

5 Brasil. Ministério da Saúde. Vigilância em saúde no Brasil 2003|2019: da criação da Secretaria de Vigilância em Saúde aos dias atuais. 2019. <https://www.saude.gov.br/images/pdf/2020/janeiro/20/Boletim-epidemiologico-SVS-02-1-.pdf> (accessed May 20, 2020).
