# Supplementary material for: Field and classroom initiatives for portable sequence-based monitoring of dengue virus in Brazil
Source: Nat Commun. 2021 Apr 16;12:2296. doi: 10.1038/s41467-021-22607-0 (PMC8052316; doi:10.1038/s41467-021-22607-0)
Supplement: Supplementary file 2 — Reporting Summary [file 41467_2021_22607_MOESM2_ESM.pdf]

nature research

Corresponding author(s): Marta Giovanetti, José Lourenço, Luiz Carlos Junior Alcantara

Last updated by author(s): Mar 2, 2021

Reporting Summary

Nature Research wishes to improve the reproducibility of the work that we publish. This form provides structure for consistency and transparency in reporting. For further information on Nature Research policies, see our Editorial Policies and the Editorial Policy Checklist.

Statistics

For all statistical analyses, confirm that the following items are present in the figure legend, table legend, main text, or Methods section.

n/a	Confirmed	
☐	☒	The exact sample size (*n*) for each experimental group/condition, given as a discrete number and unit of measurement
☒	☐	A statement on whether measurements were taken from distinct samples or whether the same sample was measured repeatedly
☐	☒	The statistical test(s) used AND whether they are one- or two-sided<br>*Only common tests should be described solely by name; describe more complex techniques in the Methods section.*
☒	☐	A description of all covariates tested
☒	☐	A description of any assumptions or corrections, such as tests of normality and adjustment for multiple comparisons
☐	☒	A full description of the statistical parameters including central tendency (e.g. means) or other basic estimates (e.g. regression coefficient) AND variation (e.g. standard deviation) or associated estimates of uncertainty (e.g. confidence intervals)
☐	☒	For null hypothesis testing, the test statistic (e.g. *F*, *t*, *r*) with confidence intervals, effect sizes, degrees of freedom and *P* value noted<br>*Give P values as exact values whenever suitable.*
☐	☒	For Bayesian analysis, information on the choice of priors and Markov chain Monte Carlo settings
☒	☐	For hierarchical and complex designs, identification of the appropriate level for tests and full reporting of outcomes
☒	☐	Estimates of effect sizes (e.g. Cohen's *d*, Pearson's *r*), indicating how they were calculated

Our web collection on statistics for biologists contains articles on many of the points above.

Software and code

Policy information about availability of computer code

Data collection	Those informations can be find into the Methods section of the main document
Data analysis	Software used: Guppy (v3.4.5), qcat (v1.1.0), Genome Detective online (https://www.genomedetective.com/), Dengue Virus Typing Tool online (https://www.genomedetective.com/app/typingtool/dengue/), MAFFT (v7.427), Aliview (v1.26), IQ-TREE (which in turn include the ModelFinder package)(v1.6.11), TempEst (v1.5.3), BEAST (v1.10.4), BEAST2 (v2.4), Tree Annotator (v1.10.4), FigTree (v1.4.4), LogCombiner (v1.10.4).

For manuscripts utilizing custom algorithms or software that are central to the research but not yet described in published literature, software must be made available to editors and reviewers. We strongly encourage code deposition in a community repository (e.g. GitHub). See the Nature Research guidelines for submitting code & software for further information.

Data

Policy information about availability of data

All manuscripts must include a data availability statement. This statement should provide the following information, where applicable:
- Accession codes, unique identifiers, or web links for publicly available datasets
- A list of figures that have associated raw data
- A description of any restrictions on data availability

All sequence data are available in GenBank, with accession numbers MT929528-MT929754 (a full list can be found at Supplementary Table S3). All data including alignments, tree files as well as epidemiological data can be found at: https://github.com/genomicsurveillance/Arbovirus-genomic-surveillance

Field-specific reporting

Please select the one below that is the best fit for your research. If you are not sure, read the appropriate sections before making your selection.

☐ Life sciences ☐ Behavioural & social sciences ☒ Ecological, evolutionary & environmental sciences

For a reference copy of the document with all sections, see nature.com/documents/nr-reporting-summary-flat.pdf

Ecological, evolutionary & environmental sciences study design

All studies must disclose on these points even when the disclosure is negative.

Study description	We combined field and classroom initiatives to provide insight on the recent transmission history of DENV in Brazil. Using portable whole genome sequencing, were generated 227 complete genomes sequences from DENV1 genotype V and DENV2 genotype III. To investigate the diversity and the origins of the DENV1 and DENV2 outbreaks in Brazil, we performed epidemiological and phylogenetic analyses, using all complete genome sequences available in GenBank from these two genotypes.
Research sample	Anonymous clinical samples from 227 patients with DENV infection (DENV1=57 and DENV2=170) obtained for routine diagnostic purposes at local health services in different Brazilian municipalities or samples from volunteer blood donors who reported adverse effects up to 14 days after donation were elegible for this study. One hundred and one (44.5%) were male, and the median age was 34 (range: 4-90) years. Samples evaluated in this study were representative of the three Brazilian regions (Southeast, Midwest and Northeast) that have historically recorded the highest incidences of dengue. Additionally, we including in our dataset all complete genome sequences of the DENV1 genotype V (DENV1-V=444) and DENV2 genotype III (DENV2-III=450) available in GenBank (except those described in the Data exclusions section).
Sampling strategy	Available samples were selected accordance with the availability of epidemiological metadata, such as date of symptom onset, date of sample collection, sex, age, municipality of residence, symptoms, disease classification, as well as the Ct value (≤35) obtained by RT-qPCR and sufficient DNA concentration (≥2ng/µL) to proceed to library preparation. All available samples that met these criteria were included in this study and subjected to the complete genome sequencing of DENV1 and DENV2.
Data collection	The viral genomes obtained in this study were obtained from available samples selected accordingly with the availability of epidemiological metadata, such as date of symptom onset, date of sample collection, sex, age, municipality of residence, symptoms, disease classification, as well as the Ct value (≤35) obtained by RT-qPCR and sufficient DNA concentration (≥2ng/µL) to proceed to library preparation. Reference sequences were selected based on the availability of collection date, location as well as sequences covering more than 50% of the viral genome.
Timing and spatial scale	DENV1 and DENV2 dataset, including the sequences generated in this study, comprised isolates collected between 1956-2019 and 1986-2019, respectively, and from different countries in America, Asia and Africa to maximise geographic and temporal distribution.
Data exclusions	The pre-established exclusion criteria were based on the unavailability of metadata, such as date and sampling location, as well as sequences covering less than 50% of the viral genome.
Reproducibility	The generation of the DENV complete genomes was not carried out in replicates, however, all the 227 sequences generated in this study were obtained using a methodoly widely used in the literature (refs: 18, 22, 23, 34, 37 and 39 of the manuscript). In each sequencing run we used negative controls to prevent and check for possible contamination with less than 2% mean coverage. For phylogeographic analysis, MCMC runs were performed in duplicate for 100 million iterations to ensure stationarity and an adequate effective sample size.
Randomization	Randomization was not necessary, since we did not performed a case-control study. Furthermore, all the clinical samples available that met the inclusion criteria for this study (Sampling strategy section) were subjected to sequencing and we included all the DENV1 genotype V and DENV2 genotype III complete sequences available at Genbank (except those described in the Data exclusions section) for evolutionary analysis.
Blinding	This study does not include experimental groups, so blinding was not relevant.

Did the study involve field work? ☐ Yes ☒ No

Reporting for specific materials, systems and methods

We require information from authors about some types of materials, experimental systems and methods used in many studies. Here, indicate whether each material, system or method listed is relevant to your study. If you are not sure if a list item applies to your research, read the appropriate section before selecting a response.

Materials & experimental systems

n/a	Involved in the study
☒	☐ Antibodies
☒	☐ Eukaryotic cell lines
☒	☐ Palaeontology and archaeology
☒	☐ Animals and other organisms
☐	☒ Human research participants
☒	☐ Clinical data
☒	☐ Dual use research of concern

Methods

n/a	Involved in the study
☒	☐ ChIP-seq
☒	☐ Flow cytometry
☒	☐ MRI-based neuroimaging

Human research participants

Policy information about studies involving human research participants

Population characteristics	The study population consisted of 227 clinical samples from patients with DENV infection (DENV1=57 and DENV2=170) obtained for routine diagnostic purposes at local health services in different Brazilian municipalities or from volunteer blood donors who reported adverse effects up to 14 days after donation. The median age of patients was 28 years (range: 4 to 78 years of age) for DENV1 and 37 years (range: 4 to 90 years of age) for DENV2 cases. With respect to the clinical outcome, 77% of DENV1 (44/57) and 81% of DENV2 (138/170) samples were obtained from patients without alarming clinical signs, 44% of DENV1 (2/57) and 2% of DENV2 (4/170) corresponded to patients with severe dengue. Finally, 19% of DENV1 (11/57) and 17% of DENV2 (28/170) were obtained from fatal cases. Epidemiological details of the samples are provided in Table S2.
Recruitment	The recruitment was based on the availability of epidemiological metadata, such as date of symptom onset, date of sample collection, sex, age, municipality of residence, symptoms, disease classification, as well as the Ct value (≤35) obtained by RT-qPCR and sufficient DNA concentration (≥2ng/µL) to proceed to library preparation. Of the 248 samples initially selected, 227 samples met the criteria mentioned above and were submited to the complete genome sequencing of DENV1 (n=57) and DENV2 (n=170).
Ethics oversight	This project was reviewed and approved by the Pan American Health Organization Ethics Review Committee (PAHOERC) (Ref. No. PAHO-2016-08-0029) and the Oswaldo Cruz Foundation Ethics Committee (CAAE: 90249218.6.1001.5248). The availability of these samples for research purposes during outbreaks of national concern is allowed to the terms of the 510/2016 Resolution of the National Ethical Committee for Research – Brazilian Ministry of Health (CONEP - Comissão Nacional de Ética em Pesquisa, Ministério da Saúde), that authorize, without the necessity of an informed consent, the use of clinical samples collected in the Brazilian Central Public Health Laboratories to accelerate knowledge building and contribute to surveillance and outbreak response. The samples processed in this study were obtained anonymously from material exceeding the routine diagnosis of arboviruses in Brazilian public health laboratories that belong to the public network within BrMoH.

Note that full information on the approval of the study protocol must also be provided in the manuscript.
